# Controlled aqueous nanoassembly-driven fully automated Fmoc solid-phase peptide synthesis (SPPS) with in-water coupling

**DOI:** 10.1039/d6ra06336e

**Published:** 2026-09-10

**Authors:** Keiko Hojo, Mutsumi Kishimoto, Ayana Nagai, Cédric Rentier, Amit Mehrotra, Kazuhito Hioki, Munetaka Kunishima

**Affiliations:** a Faculty of Pharmaceutical Sciences, Kobe Gakuin University Kobe 650-8586 Japan hojo@pharm.kobegakuin.ac.jp kunisima@pharm.kobegakuin.ac.jp; b Cooperative Research Center of Life Sciences, Kobe Gakuin University Kobe 650-8586 Japan; c Biotage Japan Ltd Tokyo 136-0071 Japan; d Biotage Sweden AB Uppsala 753-18 Sweden

## Abstract

Fmoc-based solid-phase peptide synthesis (SPPS) is a cornerstone of modern peptide manufacturing; however, its reliance on dimethylformamide (DMF)—a toxic and environmentally regulated solvent—poses significant sustainability challenges. Herein, we report a controlled aqueous nanoassembly-based strategy that enables fully automated Fmoc-SPPS under water-based coupling conditions. Aqueous nanoassemblies of Fmoc-protected amino acids, generated through a simple two-step preparation protocol, provide a stable and reactive coupling environment compatible with automated synthesis. The methodology was implemented on a commercially available peptide synthesizer and applied to peptides of varying lengths and sequence complexities, affording products of acceptable purity. Importantly, the platform was applied to the automated synthesis of long-chain peptides exceeding 30 residues, including GLP-1 (1–37), demonstrating its applicability to challenging sequences. At the time of writing, this represents, to the best of our knowledge, one of the earliest fully automated Fmoc-SPPS platforms employing water-based coupling cycles without the use of DMF. By eliminating DMF and employing environmentally benign solvents such as water and ethyl acetate, the present methodology provides a practical and sustainable alternative to conventional SPPS, advancing green practices in automated peptide synthesis.

## Abstract 2

A controlled two-step nanoassembly method was integrated with fully automated Fmoc-SPPS using in-water coupling, providing reactive interfacial microenvironments for synthesis of peptides including long-chain sequences exceeding 30 residues.

## Graphical abstract

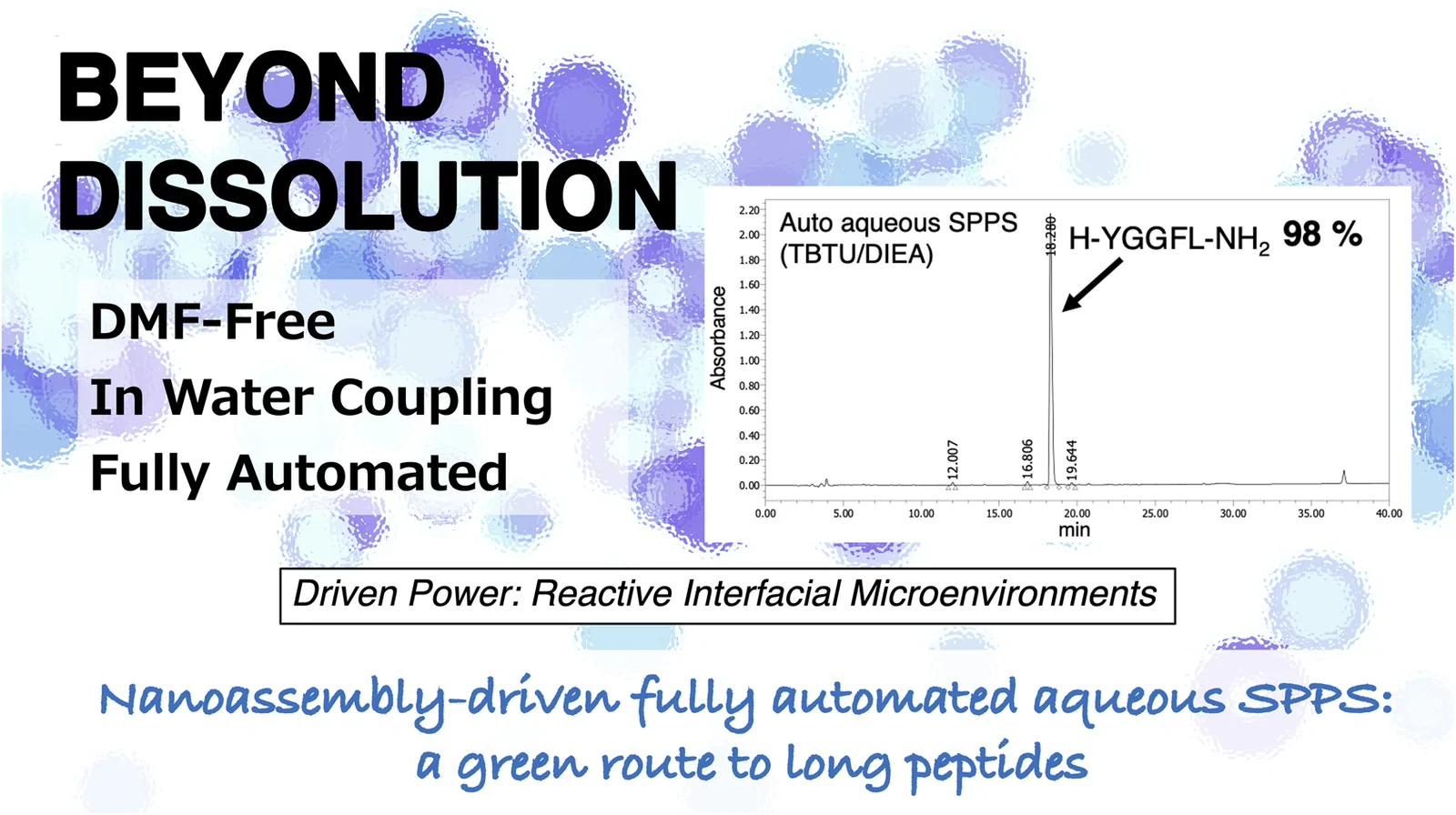

## 1. Introduction

Growing environmental awareness and increasingly stringent regulations have intensified efforts to replace hazardous organic solvents with safer and more sustainable alternatives.^1,2^ Peptide synthesis, which plays a central role in pharmaceutical research and development,^3–5^ remains highly dependent on organic solvents despite major advances in synthetic methodology. Among these solvents, *N*,*N*-dimethylformamide (DMF) is extensively used throughout peptide synthesis processes and has attracted particular concern because of its environmental and toxicological impacts. Following its classification as a hazardous substance under the European Union REACH regulations, increasingly strict restrictions have been imposed on its handling and use.^6^

These developments have stimulated considerable interest in the development of environmentally benign peptide synthesis technologies that minimize or eliminate the use of DMF. Notable efforts in this direction include the replacement and recycling of DMF using alternative organic solvents such as γ-valerolactone,^7^ as well as resin engineering strategies compatible with recyclable organic solvent systems.^8^ In particular, water has emerged as an attractive alternative reaction medium from the perspective of green chemistry. Although numerous water-based peptide synthesis methods have been reported, their practical implementation has remained limited owing to challenges associated with reaction efficiency and compatibility with commonly used amino acid building blocks.

Among the various strategies available for chemical peptide synthesis, solid-phase peptide synthesis (SPPS)^9^ remains the dominant approach because of its operational simplicity, suitability for automation, and broad sequence applicability.^10,11^ Motivated by the need for more sustainable peptide manufacturing, we have been interested in developing aqueous SPPS methodologies that can serve as viable alternatives to conventional DMF-based processes. A major obstacle in aqueous SPPS arises from the limited aqueous solubility of standard Fmoc-protected amino acids.^12^ Although a variety of water-soluble amino acid derivatives have been introduced to address this issue,^13–20^ these reagents often display reduced stability and slower coupling kinetics, resulting in restricted substrate scope and lower crude peptide quality.

Although the poor aqueous solubility of Fmoc-protected amino acids has long been regarded as a major obstacle to water-based peptide synthesis, recent studies have demonstrated that these building blocks can be utilized under aqueous conditions. In most reported approaches, Fmoc-amino acids are rendered compatible with water through the use of organic co-solvents, surfactants, or other solubilization strategies, as exemplified by the work of Albericio and co-workers.^21^ More recently, Wellings, Wade, and co-workers demonstrated that Fmoc-amino acids can be rendered water-soluble at high concentration through salt formation with organic bases such as *N*-methylmorpholine or triethanolamine, further expanding the scope of aqueous Fmoc-SPPS.^22^ While this approach significantly broadens the substrate scope of water-based peptide synthesis, it fundamentally relies on the molecular-level dissolution of the amino acid building blocks in a homogeneous aqueous solution, and, like other solubilization-based strategies, often requires auxiliary additives and can exhibit sequence-dependent coupling efficiency.

To address these limitations, we pursued an alternative concept that exploits, rather than overcomes, the intrinsic water-insolubility of Fmoc-protected amino acids. In our previous studies, we developed an aqueous SPPS methodology based on water-dispersible nanoparticles composed of Fmoc-amino acids.^23–27^ This strategy enabled efficient peptide bond formation under aqueous conditions while maintaining compatibility with the widely used Fmoc chemistry. Interestingly, peptide coupling proceeded more efficiently when the amino acids were employed as dispersed nanoparticles than when soluble derivatives were used. Furthermore, racemization during coupling was found to be suppressed under these conditions. Despite these advantages, the nanoparticle-based system exhibited several practical limitations. Preparation of the nanoparticles required wet milling using a planetary ball mill, and the resulting particles gradually aggregated in water owing to their limited colloidal stability. These characteristics hindered implementation in automated synthesis platforms and reduced the scalability of the method.

Extending this concept while overcoming its colloidal limitations, we subsequently demonstrated that Fmoc-protected amino acids can form stable aqueous nanoassemblies that promote efficient peptide bond formation under water-based conditions.^28^ Rather than achieving aqueous compatibility through molecular dissolution, these nanoassemblies deliberately retain a non-molecularly dissolved, heterogeneous state, providing reactive interfacial microenvironments that enable pronounced reaction acceleration.^29,30^ This heterogeneous, interfacially driven mechanism is mechanistically distinct from homogeneous, solubilization-based strategies such as salt-mediated dissolution, and highlights the potential of nanoassembly-based systems as a complementary route for aqueous peptide synthesis.

Building on this conceptual framework, we herein report a controlled aqueous nanoassembly-based strategy that enables fully automated Fmoc-SPPS under water-based coupling conditions. At the time of this writing, this represents, to the best of our knowledge, one of the earliest examples of fully automated Fmoc-SPPS achieved through aqueous coupling cycles without the use of DMF. By controlling the nanoassembled reaction environment rather than relying on conventional solubilization approaches, this method provides a practical and sustainable route toward next-generation peptide synthesis.

## 2. Results and discussion

Recently, we discovered that blending three components—Fmoc-protected amino acids, water-soluble condensing agents with salt structures, and organic bases—in specific ratios results in the spontaneous formation of aqueous nanoassemblies with an average diameter of approximately 25 nm.^28^ These nanoassemblies are smaller and more stable than the previously reported water-dispersible nanoparticles. Their preparation is remarkably simple, requiring only mixing in water, and the method is broadly applicable to all common Fmoc-amino acids, including non-natural derivatives. In aqueous reactions, these nanoassemblies exhibit the same rate-accelerating effects as earlier nanoparticle systems. Crucially for automated synthesis applications, they remain optically transparent and show minimal aggregation, properties that make them highly attractive for fully automated solid-phase peptide synthesis.

Despite these advantages, we observed that some three-component nanoassemblies gradually became cloudy or formed gel-like aggregates over time, which represents a serious obstacle for fully automated synthesis processes that require prolonged operation and reliable fluid handling. To address this limitation, we developed a two-step nanoassembly preparation strategy. In the first step, stable two-component nanoassemblies are formed from Fmoc-protected amino acids and an organic base in water. In the second step, an aqueous solution of a coupling reagent is added immediately before the coupling reaction, generating reactive three-component nanoassemblies *in situ* within the reaction vessel (Fig. 1). This approach was designed to preserve the high reactivity of the nanoassemblies while improving their temporal stability and compatibility with automated synthesis systems. In this context, the term “controlled nanoassembly” refers to the intentionally designed and reproducible formation of nanoassemblies through this two-step, multi-component assembly process, rather than to strict particle monodispersity.

**Fig. 1:**
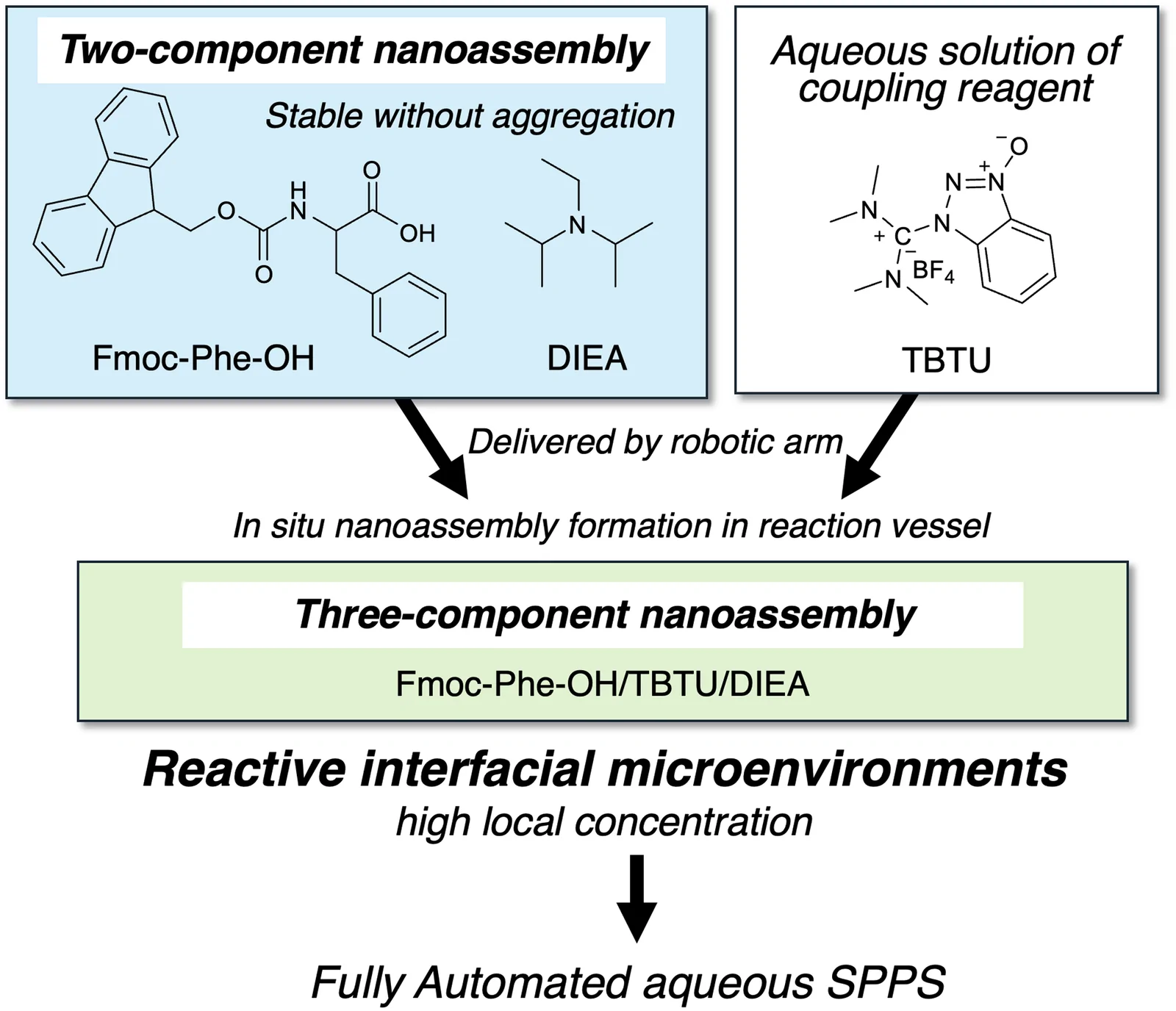
Conceptual illustration of nanoassembly-based reaction field formation for automated aqueous SPPS.

First, we prepared a two-component nanoassembly composed of Fmoc-Phe-OH and *N*,*N*-diisopropylethylamine (DIEA), and then examined the formation of a corresponding three-component assembly upon addition of a third component, 2-(1*H*-benzotriazole-1-yl)-1,1,3,3-tetramethylaminium tetrafluoroborate (TBTU).^31^ The two-component nanoassembly exhibited a clear Tyndall effect (Fig. 2A), suggesting the presence of a dispersed nanoscale species. The size distribution of these assemblies was characterized by intensity-weighted dynamic light scattering (DLS). The DLS measurements show that the two-component system exhibits a heterogeneous size distribution, with a minor population at approximately 2.5 nm alongside larger assemblies centered at ∼73 nm and ∼226 nm (Fig. 2B). The presence of species in the single-digit nanometer range may reflect partially dissolved or loosely associated building blocks coexisting with the predominant assembled states. In contrast, upon addition of TBTU as the third component, this heterogeneous distribution reorganizes into a single, more uniform population centered at ∼67 nm, with no detectable species in the single-digit nanometer range (Fig. 2C), consistent with complete reorganization of the building blocks into assembled states. Although the presence of single-nanoscale species in the two-component system depended on the amino acid and conditions, this did not interfere with the formation of reactive three-component assemblies. Instead, the addition of a third component consistently reorganized the system into assembled states, irrespective of the initial size distribution (Tables S2 and S3), thereby generating reactive interfacial microenvironments that can provide a robust reaction field for efficient peptide coupling.

**Fig. 2:**
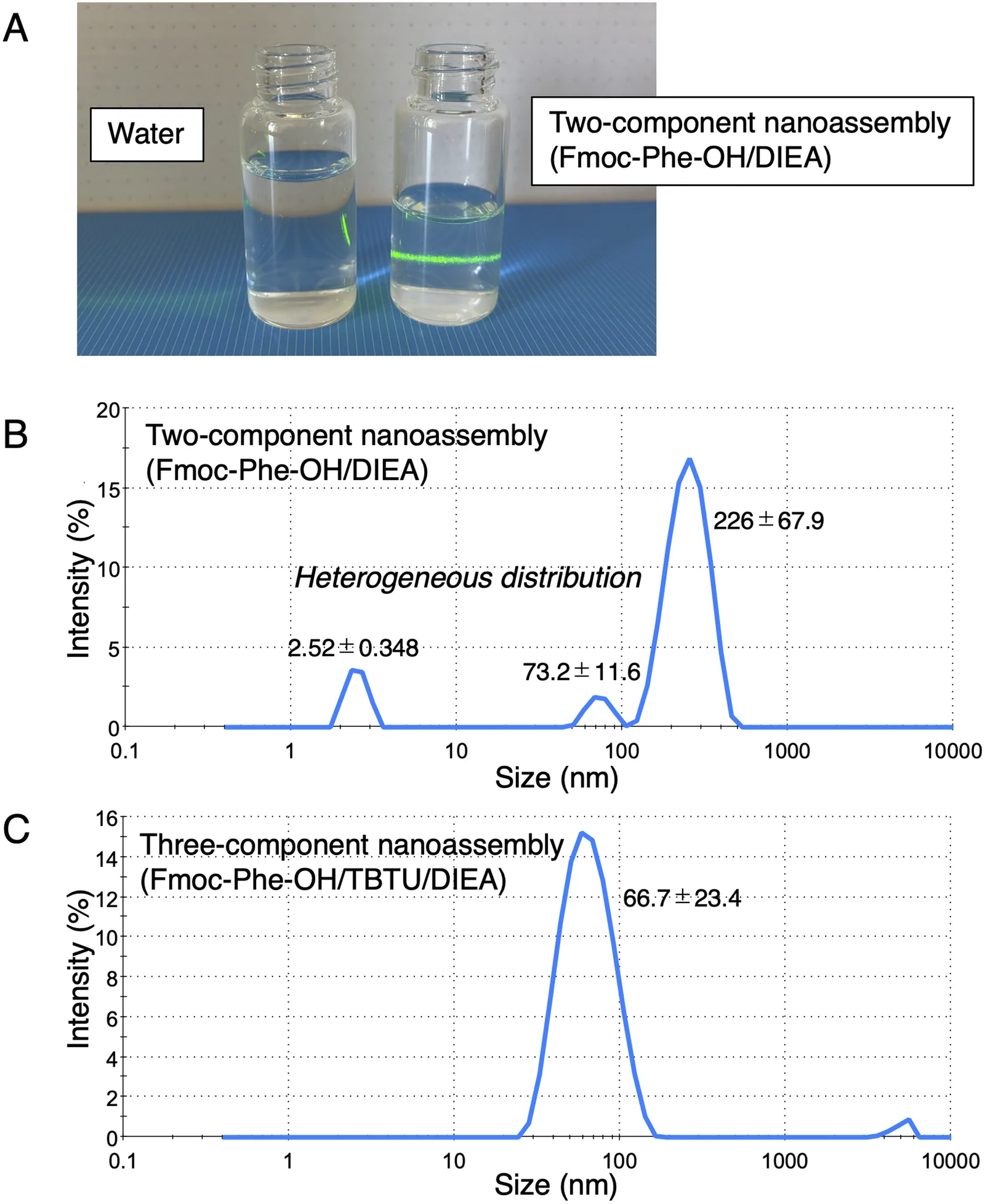
Formation of two- and three-component nanoassemblies in water. (A) Photographs of water and the two-component nanoassembly (Fmoc-Phe-OH/DIEA); (B) particle size distribution of the two-component nanoassembly (Fmoc-Phe-OH/DIEA) obtained by dynamic light scattering; (C) particle size distribution of the three-component nanoassembly (Fmoc-Phe-OH/TBTU/DIEA) obtained by dynamic light scattering.

To evaluate the nanoassembly strategy under practically relevant conditions, we investigated the aqueous solid-phase coupling of Fmoc-Phe-OH with H-Gly-Rink amide TentaGel resin^32^ using an automated microwave peptide synthesizer (Biotage® Initiator+ Alstra™). TentaGel resin was selected for its PEG-grafted polystyrene core, which swells well in water and provides sufficient mechanical robustness. In previous studies, fully PEG-based resins were also evaluated under related aqueous conditions; however, resin degradation limited successful chain elongation.^33^ TentaGel was therefore selected as a practical compromise between aqueous swelling ability and mechanical robustness. Two-component nanoassemblies composed of Fmoc-Phe-OH and DIEA were prepared in water and placed in the amino acid rack of the Initiator+ Alstra system. An aqueous solution of the coupling reagent TBTU was loaded into the designated reagent position of the synthesizer, and a microwave-assisted coupling program was initiated. During the automated operation, the resin was placed in the reaction vessel, followed by sequential robotic injection of the two-component nanoassembly solution and the coupling reagent solution. The coupling reaction was conducted under microwave irradiation at 70 °C with automated temperature control. In addition to TBTU, other water-soluble coupling reagents,^31^ including 4-(4,6-dimethoxy-1,3,5-triazin-2-yl)-4-methylmorpholinium chloride (DMT-MM),^34,35^ 1-[bis(dimethylamino)methylene]-1*H*-1,2,3-triazolo[4,5-*b*]pyridinium 3-oxide tetrafluoroborate (TATU),^31^ and 1-[bis(dimethylamino)methylen]-5-chlorobenzotriazolium 3-oxide tetrafluoroborate (TCTU), were also evaluated under identical conditions. 1-Ethyl-3-(3-dimethylaminopropyl)carbodiimide hydrochloride (EDCI) and 1-hydroxybenzotriazole (HOBt) based activation was not evaluated in the present study, as carbodiimide-based systems have previously shown limited performance under aqueous conditions in our earlier work, and the present platform was designed to minimize the number of components. The results of these coupling experiments are summarized in Table 1. In all cases, the aqueous solid-phase couplings proceeded smoothly and reached completion within 5 min. These results demonstrate that the rate acceleration previously observed for pre-formed three-component nanoassemblies is fully retained in the two-step preparation protocol, regardless of the preparation procedure, indicating reproducible nanoassembly formation. Furthermore, the two-component nanoassembly solutions exhibited excellent stability at room temperature over extended periods while maintaining the Tyndall effect, indicating persistent nanoscale organization. This stability is particularly advantageous for the automated synthesis of long-chain peptides, which requires extended synthesis times. Collectively, these findings support the reliability of the nanoassembly-based reaction environment under automated conditions.

**Table 1:** Aqueous solid-phase coupling study using two-component nanoassembly and aqueous coupling reagent solution^a^

Entry	Coupling reagent	Time (min)	Ninhydrin test
1	TBTU	5	Negative
2	TBTU	1	Slightly positive
3	TATU	5	Negative
4	TCTU	5	Negative
5	DMT-MM	5	Negative

a Coupling reactions were performed using two-component Fmoc-Phe-OH nanoassemblies and aqueous coupling reagent solutions under microwave irradiation at 70 °C. All coupling studies were performed on H-Gly-Rink amide TentaGel resin.

Having established that the two-step nanoassembly strategy enables rapid and stable aqueous coupling under fully automated conditions, we evaluated the automated SPPS of a short-chain model peptide, Leu-enkephalin amide (H-YGGFL-NH_2_), using nanoassemblies. The automated synthesis was carried out according to the two-step protocol illustrated in Scheme 1. Briefly, each coupling cycle involved sequential robotic delivery of the two-component nanoassembly solution followed by the coupling reagent solution, with microwave irradiation at 70 °C for 5 min. Fmoc deprotection was performed using 20% 4-methylpiperidine in EtOAc at 40 °C for 5 min. 4-Methylpiperidine was selected as an alternative deprotection reagent to piperidine because, unlike piperidine, it is not subject to legal control as a precursor chemical for illegal psychotropic drug synthesis, facilitating its procurement for scalable synthesis processes.^36^ EtOAc is widely recognized as a green solvent owing to its favorable environmental profile.^37^ Consequently, this protocol completely avoids the use of DMF, relying solely on water and green solvents, thereby enhancing its environmental compatibility. An overview of the fully automated, DMF-free aqueous Fmoc-SPPS cycle is shown in Fig. 3. Each Fmoc-amino acid was introduced as a nanoassembly in the order corresponding to the target sequence. After completion of the SPPS cycles, the peptide was cleaved from the resin using TFA and appropriate scavengers (Section S3.1 in the SI). HPLC analysis of the crude H-YGGFL-NH_2_ product revealed a single sharp peak with no detectable deletion sequences or side products (Fig. 4A), affording a crude purity of 98% with TBTU/DIEA, which is comparable to or higher than that obtained by conventional automated SPPS in DMF (96%, Fig. 4C). Among the coupling reagents evaluated, TBTU gave the highest crude purity under the present conditions. No additional chromatographic peaks attributable to diastereomer formation were observed in the analytical HPLC chromatogram, consistent with the absence of significant racemization. In addition, no signals corresponding to the characteristic +98 Da mass shift associated with tetramethylguanidinylation were observed in the mass spectrum (Fig. S22B). Although DMT-MM/NMM afforded somewhat lower crude purity (91%, Fig. 4B) under the conditions examined here, this reagent combination remains a promising candidate, as the assembly composition and reaction conditions have not yet been fully optimized.

**Scheme 1:**
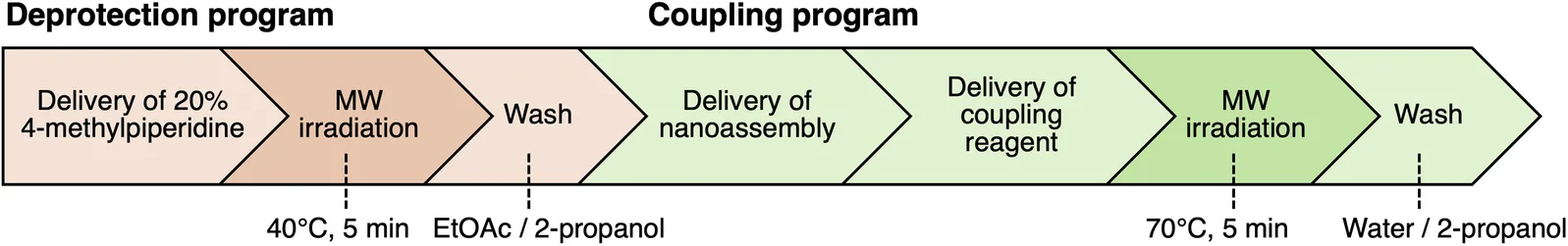
Tentative programmed workflow for automated aqueous SPPS *via in situ* nanoassembly formation using 20% 4-methylpiperidine. Initial programmed workflow employed for automated aqueous SPPS using microwave-assisted deprotection and coupling steps. The process consisted of Fmoc deprotection using 20% 4-methylpiperidine, followed by coupling *via in situ* formation of amino acid nanoassemblies and subsequent addition of an aqueous coupling reagent solution.

**Fig. 3:**
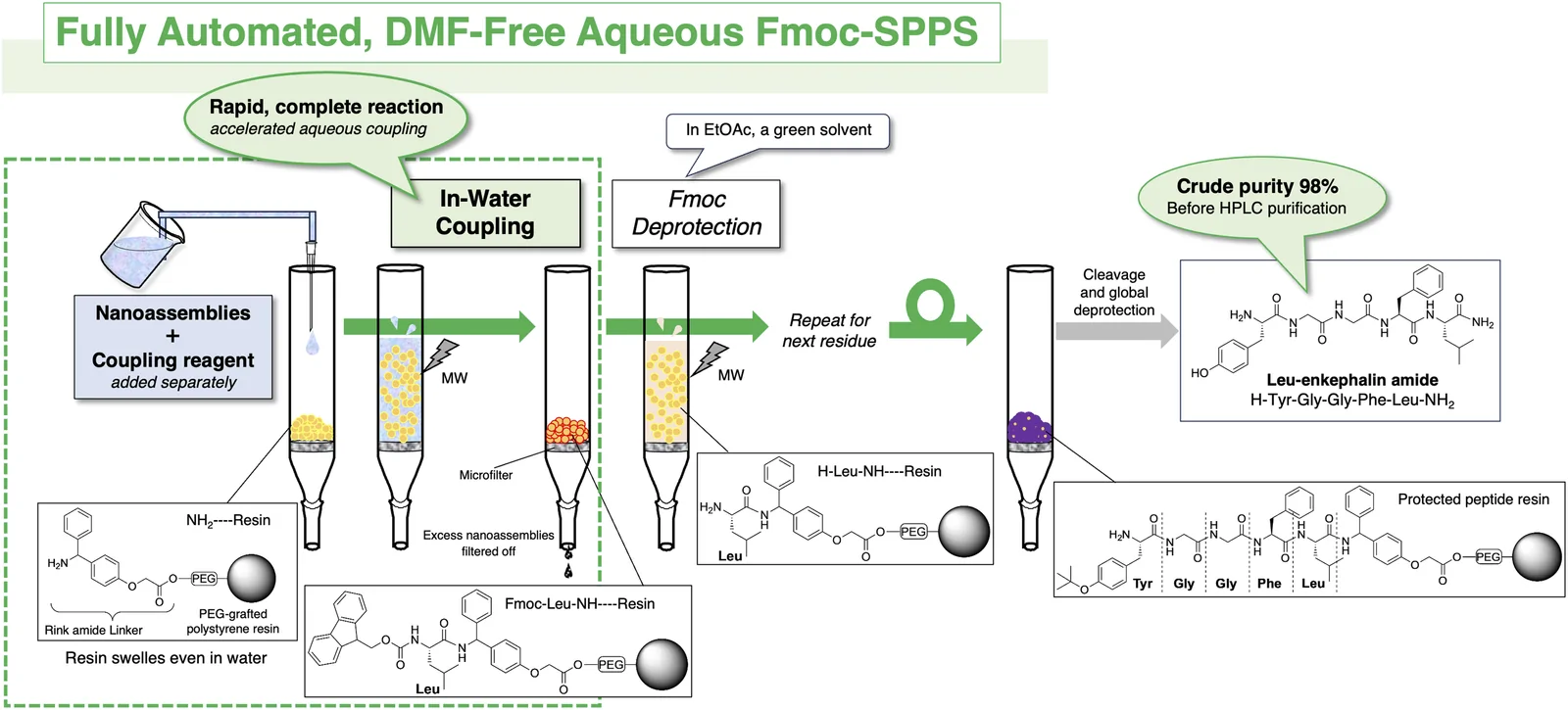
Overview of the fully automated, DMF-free aqueous Fmoc-SPPS cycle.

**Fig. 4:**
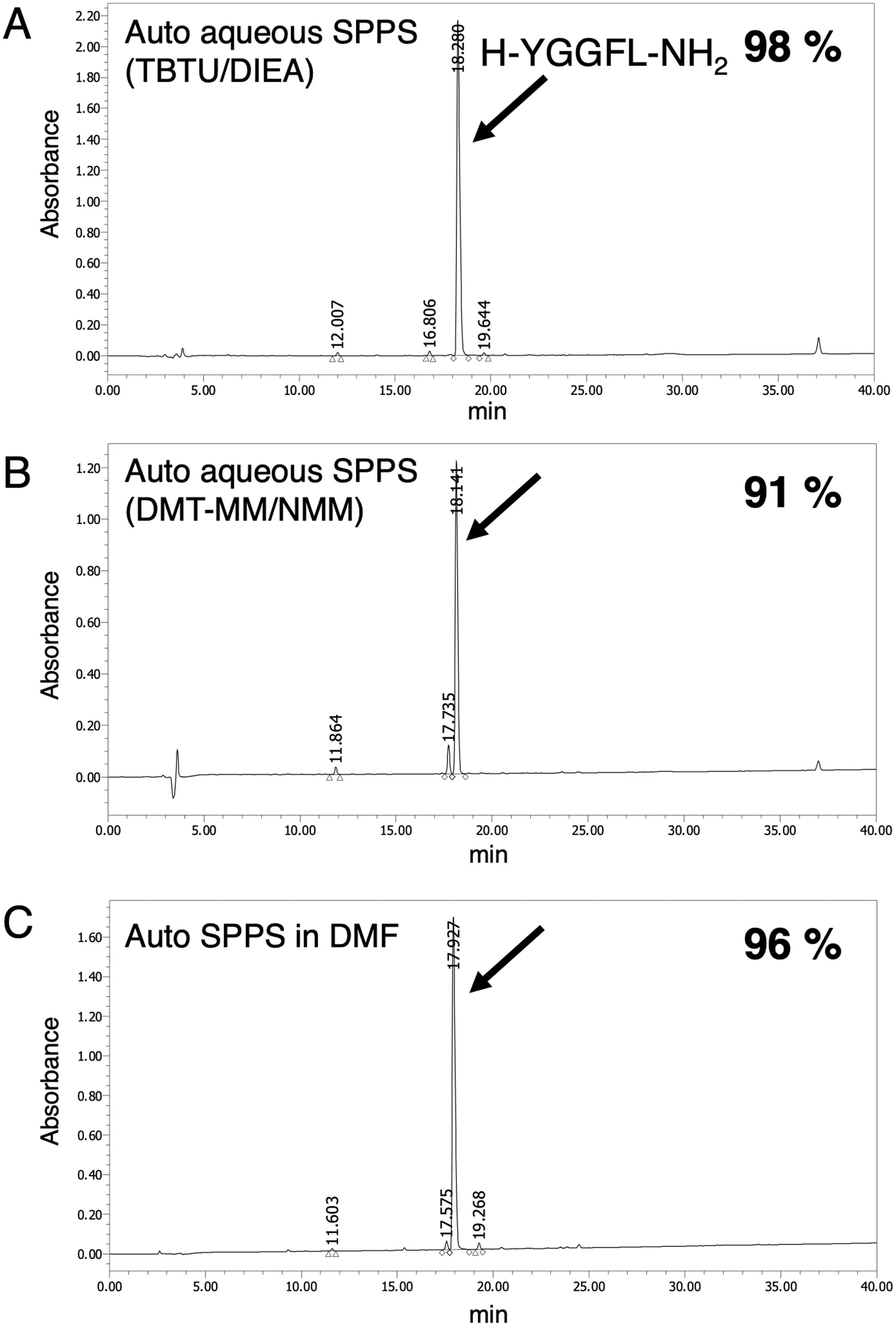
Comparison of automated SPPS of Leu-enkephalin amide under aqueous and conventional conditions. (A) Automated aqueous SPPS using TBTU/DIEA; (B) automated aqueous SPPS using DMT-MM/NMM; (C) conventional automated SPPS in DMF. Numbers% indicate estimated crude purity determined by HPLC. Elution was carried out over 40 min at a flow rate of 1 mL min^−1^ with a linear gradient from 90 : 10 to 50 : 50 mixture of 0.1% aqueous TFA and 0.1% TFA in acetonitrile.

The entire synthesis was completed efficiently, demonstrating the practicality of this approach for rapid, automated peptide synthesis under environmentally benign conditions. To quantitatively assess the environmental impact of the aqueous coupling strategy, PMI values were calculated for the synthesis of H-YGGFL-NH_2_ and compared with those of a conventional DMF-based SPPS protocol (Section S5 in the SI). Although the PMI values were comparable under the conditions examined, the present protocol completely avoided the use of DMF.

To further evaluate the robustness of this method, we investigated the automated synthesis of a longer, more demanding peptide sequence, Substance P (H-RPKPQQFFGLM-NH_2_). This peptide contains Pro-X-Pro motifs prone to aggregation as well as consecutive Gln residues bearing bulky trityl protecting groups.^38^ Initial attempts using the automated SPPS programmed workflow (Scheme 1) with a coupling time of 10 min were unsuccessful due to inefficient Fmoc deprotection with 20% 4-methylpiperidine in EtOAc (Fig. S25). This was attributed to prolonged storage of 4-methylpiperidine in EtOAc within the Initiator+ Alstra system, which may have led to partial degradation and a consequent decrease in the nucleophilicity of the base.^39^ To address this issue, the deprotection protocol was modified such that 4-methylpiperidine and EtOAc were mixed directly in the reaction vessel immediately prior to use (Scheme 2). The synthesis was then carried out using double coupling for the *C*-terminal Met residue, single coupling for the subsequent four residues, and double coupling for the six *N*-terminal residues (Scheme 2 and Table S4). Following cleavage with appropriate TFA and scavenger mixture, HPLC analysis of the crude product (Fig. 5A) showed that the target peptide, Substance P, was obtained as the major product with an estimated purity of 81%. Minor by-products were also detected, including Met-oxidized Substance P and a deletion product lacking one Gln residue (Fig. 5B). A comparable Met-oxidized by-product was also observed for Substance P synthesized using a conventional automated SPPS protocol in DMF (Fig. 5C). In contrast, the Gln deletion product was not observed under the conventional conditions, suggesting that consecutive coupling of Gln may be more challenging in aqueous environments. Notably, several minor by-products detected in the DMF-based synthesis were not observed in the aqueous system. Although trace levels of racemization cannot be completely excluded, no experimental observations suggesting substantial racemization were obtained under the mild aqueous coupling conditions employed in this study.^25,26^ Taken together, these results indicate that the aqueous automated synthesis method developed in this study affords product quality comparable to that achieved by conventional DMF-based SPPS.

**Scheme 2:**
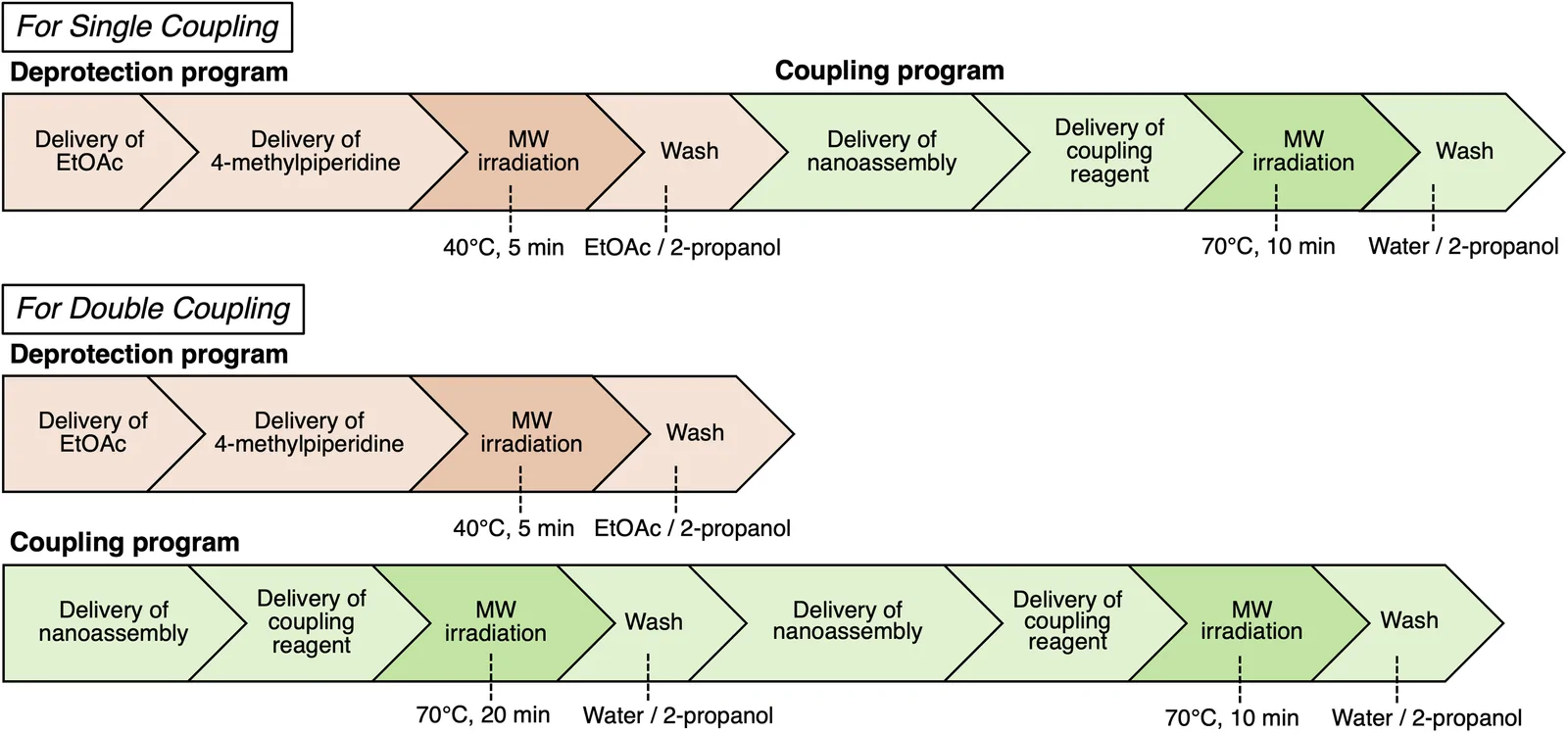
Optimized programmed workflow for coupling and deprotection steps in automated aqueous SPPS *via in situ* nanoassembly formation. Optimized automated aqueous SPPS workflow incorporating modified deprotection and coupling sequences for single- and double-coupling protocols. Coupling reactions were performed through sequential delivery of the two-component nanoassembly solution and aqueous coupling reagent solution under microwave irradiation with temperature control.

**Fig. 5:**
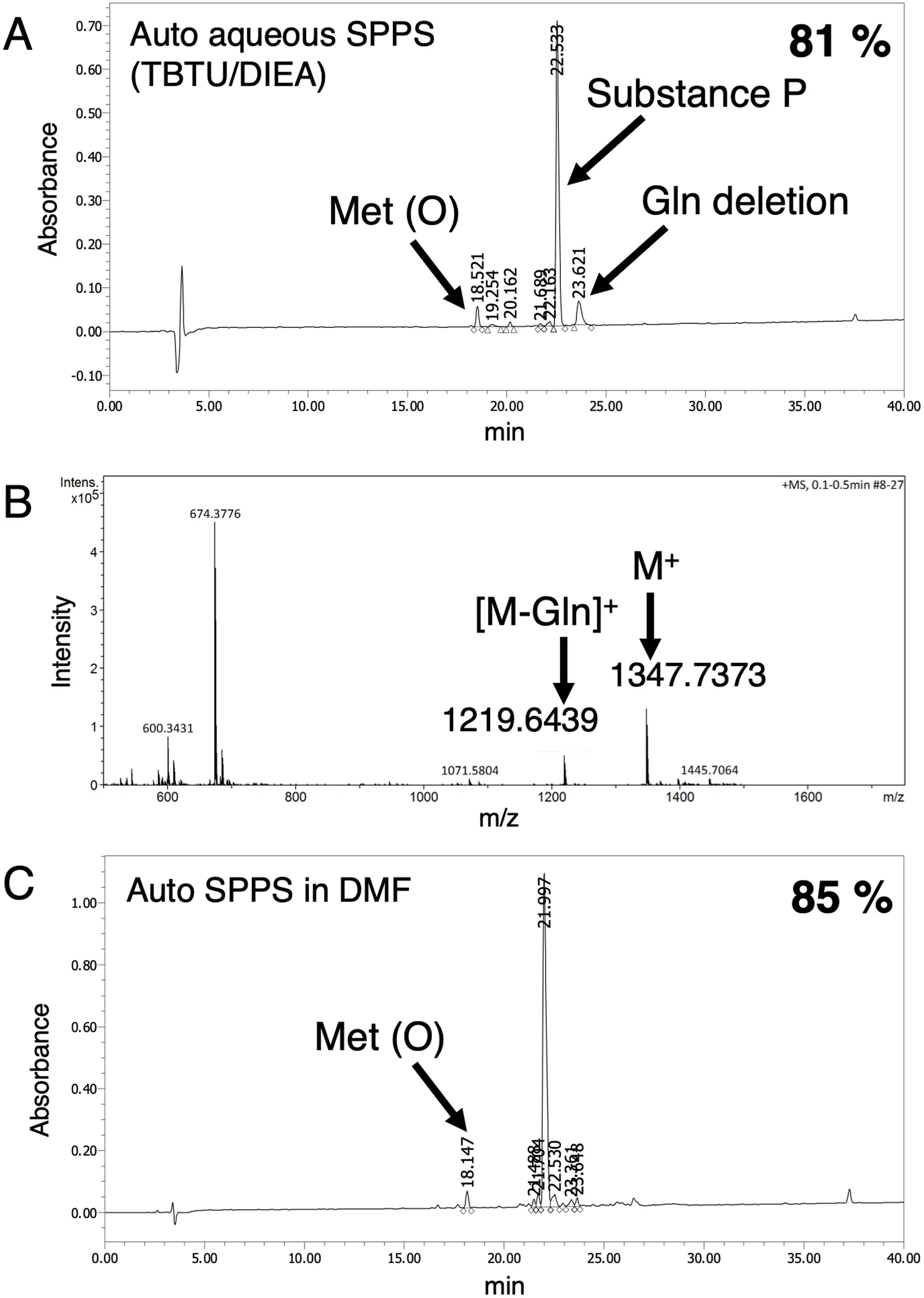
Product analysis of Substance P synthesized under aqueous and conventional automated SPPS. (A) HPLC of the crude product obtained by automated aqueous SPPS using TBTU/DIEA; (B) ESI-TOF mass spectrum of the crude product, identifying the Gln-deletion product and the intact peptide; (C) HPLC of the crude product obtained by conventional automated SPPS in DMF. Number% indicate estimated crude purity. Elution was carried out over 40 min at a flow rate of 1 mL min^−1^ with a linear gradient from 90 : 10 to 50 : 50 mixture of 0.1% aqueous TFA and 0.1% TFA in acetonitrile.

Finally, we attempted the automated synthesis of the long-chain 37-residue peptide GLP-1 (1–37) (H-HDEFERHAEGTFTSDVSSYLEGQAAKEFIAWLVKGRG-NH_2_) using the same automated program (Scheme 2). For positions anticipated to be sterically hindered and prone to inefficient coupling, a double-coupling protocol was employed. Detailed coupling cycle schedules and coupling guidelines are provided in the SI (Table S5 and Section S2.1 in the SI). Initially, all coupling reactions were carried out using TBTU. The HPLC profile was highly complex, showing multiple deletion and side-product peaks. Mass spectrometric analysis suggested the presence of species consistent with the loss of histidine residues, although the assignments remain tentative (Fig. S28). Given the tendency of His to undergo racemization, DMT-MM was therefore employed selectively as the condensing agent at the positions corresponding to the two His residues.^26^ GLP-1 (1–37) is a 37-residue peptide that presents significant synthetic challenges due to its length, hydrophobic regions, and aggregation-prone segments, and achieving high crude purity in its synthesis remains difficult even under optimized conventional conditions. HPLC analysis of the crude product revealed GLP-1 (1–37) as the major product with an estimated crude purity of 16% (Fig. 6A), comparable to that obtained under conventional DMF-based automated SPPS (18%, Fig. 6B), indicating that the aqueous nanoassembly approach achieves similar overall performance for this demanding sequence. The deletion by-products observed under aqueous and DMF-based conditions differed in their composition: under conventional DMF-based conditions, a His deletion product and a fragment lacking residues 1–8 (GLP-1 (9–37)) were the major by-products (Fig. 6B), whereas under aqueous conditions, peptides lacking residues 1–6 were predominantly observed (Fig. 6A). This difference in deletion pattern may reflect distinct aggregation or coupling kinetics in aqueous *versus* organic solvent environments, and represents an informative observation for understanding the mechanistic characteristics of aqueous SPPS. In addition, signals that may originate from resin-derived species were detected by mass spectrometric analysis (Fig. S31), suggesting that partial resin degradation may occur under the prolonged reaction conditions required for long-chain peptide synthesis; this warrants further investigation. Although the crude purity remains to be improved through systematic optimization of coupling conditions, protecting group strategies, and resin selection, these results provide proof-of-concept that fully automated aqueous SPPS is applicable to peptides exceeding 30 residues. To the best of our knowledge, at the time of this writing, this represents one of the earliest demonstrations of fully automated aqueous SPPS enabling the synthesis of such long-chain peptides.

**Fig. 6:**
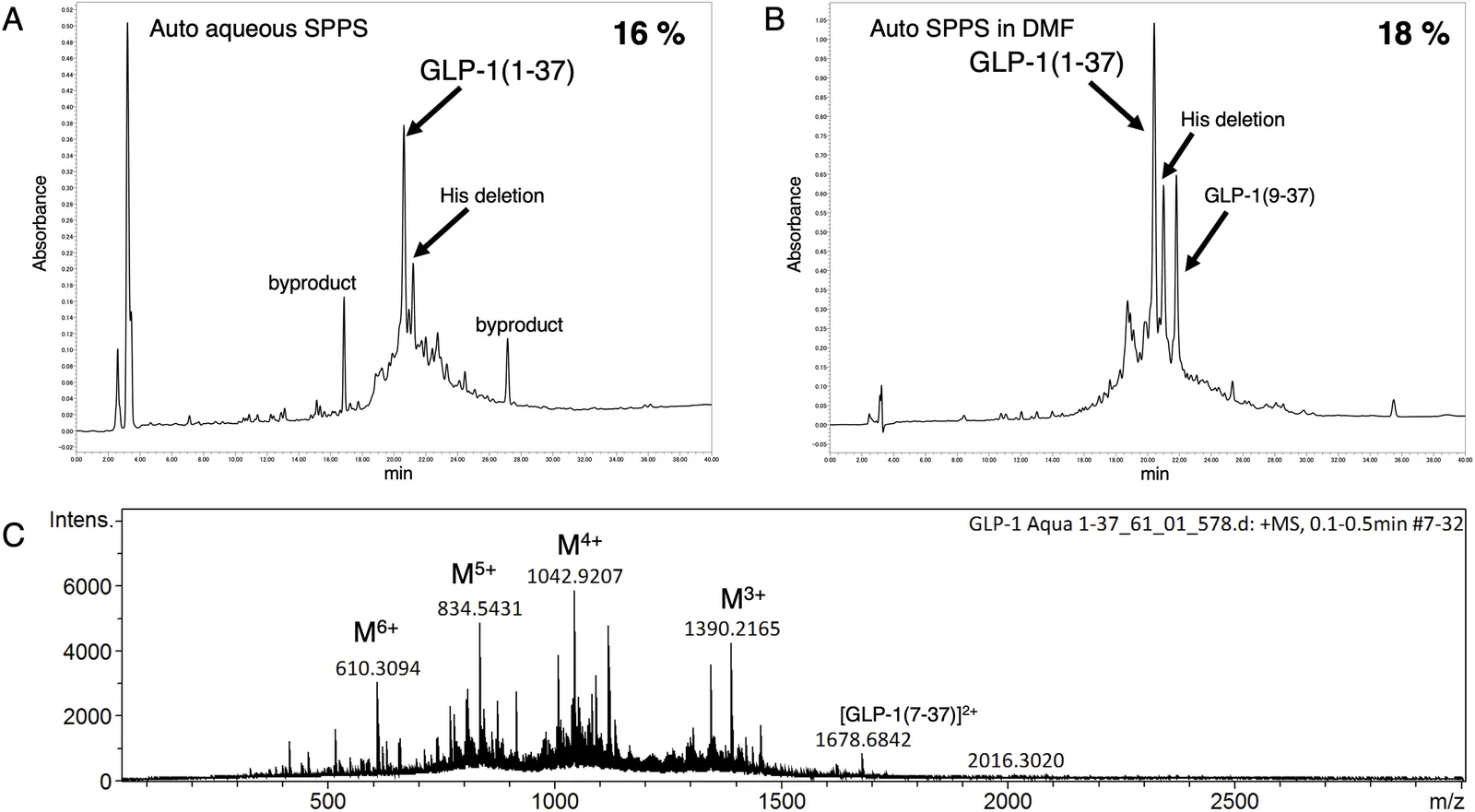
Product analysis of GLP-1 (1–37) synthesized under automated aqueous and conventional SPPS. (A) HPLC of the crude product obtained by automated aqueous SPPS; (B) HPLC of the crude product obtained by conventional automated SPPS in DMF; (C) ESI-TOF mass spectrum of the major product, showing signals consistent with intact GLP-1 (1–37). Numbers indicate estimated crude purity. Elution was carried out over 40 min at a flow rate of 1 mL min^−1^ with a linear gradient from 80 : 20 to 50 : 50 mixture of 0.1% aqueous TFA and 0.1% TFA in acetonitrile.

Collectively, these results demonstrate that the nanoassembly-based automated approach using the Initiator+ Alstra system is effective for the synthesis of long-chain peptides. Furthermore, these findings establish the nanoassembly-based automated SPPS platform as a robust and versatile method, enabling rapid synthesis of short peptides, reliable optimization for demanding sequences, and automated preparation of long-chain peptides under fully aqueous and DMF-free conditions.

## 3. Conclusion

In this study, we have developed a fully automated Fmoc-based SPPS method that eliminates the use of DMF and is instead constructed entirely from water and other environmentally benign solvents such as ethyl acetate, representing a step towards more sustainable SPPS. Central to this platform is the use of aqueous nanoassemblies, which enable efficient peptide bond formation through a simple and robust two-step preparation protocol while maintaining high reactivity under fully automated conditions. The methodology was successfully applied to the synthesis of peptides of varying length and complexity, affording products of acceptable purity, with by-product profiles that varied depending on peptide length and sequence complexity. To the best of our knowledge, this work represents an important step toward enabling fully automated SPPS using aqueous coupling chemistry for long-chain peptides. While the present study establishes the practical feasibility of this automated aqueous SPPS approach, further investigation is warranted to systematically address potential racemization at His/Cys residues and aspartimide-derived Asp/isoAsp isomerization at susceptible sequence motifs (*e.g.*, Asp–Gly), particularly in longer and more compositionally complex sequences, as well as aggregation of protected peptide chains under aqueous conditions–factors that will need to be carefully considered for broader implementation. Nevertheless, the platform presented here provides a solid foundation for the development of scalable and sustainable peptide manufacturing processes.

The operational stability and reactivity of the nanoassemblies, together with their seamless compatibility with automated peptide synthesizers, underscore the robustness, scalability, and generality of this approach. Collectively, these findings establish a viable DMF-free platform for automated peptide synthesis and open new avenues toward sustainable peptide manufacturing. Furthermore, this work highlights nanoassembly-based reaction environments as a general strategy for enabling efficient transformations under aqueous and heterogeneous conditions. We anticipate that this conceptual framework will provide a foundation for the future development of environmentally conscious and automated synthetic methodologies in peptide science and beyond.

## 4. Experimental

### 4.1 Materials and methods

Fmoc-amino acids were obtained from Watanabe Chemical Industries, Ltd (Hiroshima, Japan). Reagents and solvents were obtained from Tokyo Chemical Industry Co., Ltd (Tokyo, Japan). Particle size was determined by dynamic light scattering (DLS) using a Zetasizer Nano ZSP (Malvern Panalytical Ltd, Malvern, U.K.). Microwave (MW) reactions were performed using a Biotage® Initiator+ Alstra™ peptide synthesizer (Biotage AB, Uppsala, Sweden). Reversed-phase HPLC was carried out on a Waters Alliance e2695 system (Waters Corp., Milford, MA, U.S.A.) equipped with a COSMOSIL 5C18-AR-II column (Nacalai Tesque Inc., Kyoto, Japan) using a gradient of acetonitrile/water containing 0.1% TFA. Mass spectra were recorded on an electrospray ionization quadrupole time-of-flight mass spectrometer (ESI-Q-TOF-MS), micrOTOF-Q (Bruker Daltonik GmbH, Bremen, Germany). Detailed HPLC analysis, integration criteria, and ESI-MS acquisition protocols are provided in the SI.

### 4.2 General procedure for preparation of nanoassemblies of Fmoc-amino acids containing bases

Nanoassemblies of Fmoc-Phe-OH prepared with DIEA (Example): Fmoc-Phe-OH (39 mg, 0.1 mmol) and DIEA (22 µl, 0.12 mmol) were mixed in a small amount of water using a vortex mixer. 5 mL of water was added and mixed vigorously using a vortex mixer to form aqueous nanoassemblies. Particle size (mode diameter): 225.9 ± 67.92 nm (intensity-weighted).

### 4.3 Aqueous coupling study using a two-step nanoassembly method in Initiator+ Alstra

Coupling test reactions were performed using H-Gly-Rink amide-TentaGel resin (0.25 mmol g^−1^, 100 mg, 0.025 mmol). The resin was swollen in water, and microwave-assisted coupling reactions were carried out on an Initiator+ Alstra automated peptide synthesis system using various aqueous nanoassemblies of Fmoc-Phe-OH (0.1 mmol) in combination with a base (DIEA or NMM) and an aqueous solution of a coupling reagent (DMT-MM, TBTU, TATU, or TCTU). For each reaction, the mixture was heated to 70 °C by microwave irradiation and maintained at this temperature for 5 min. After microwave treatment, the resin was washed sequentially with water (×3) and 2-propanol (×3). The extent of coupling was evaluated by the ninhydrin (Kaiser) test. The results of these coupling tests are summarized in Table 1.

### 4.4 General procedure for fully automated aqueous SPPS in Biotage® Initiator+ Alstra™

#### 4.4.1 Leu-enkephalin amide

Leu-enkephalin amide (H-YGGFL-NH_2_) was synthesized by fully automated aqueous SPPS using an Initiator+ Alstra microwave peptide synthesizer, following the workflow summarized in Scheme 1. The system was programmed to deliver aqueous nanoassemblies of Fmoc-protected amino acids and aqueous reagent solutions directly into the reaction vessel *via* robotic liquid handling, without manual intervention during the coupling cycles. Fmoc-Rink amide-TentaGel resin (0.25 mmol g^−1^, 100 mg, 0.025 mmol) was charged into the reaction vessel and swollen in water. Aqueous nanoassemblies (5.0 mL, corresponding to 16.7 mM with respect to the Fmoc-amino acid), pre-prepared from Fmoc-amino acid (0.1 mmol) and DIEA (0.12 mmol), and an aqueous solution of TBTU (1.0 mL, 0.1 mmol) were sequentially dispensed into the vessel under full robotic control. Coupling was conducted under microwave assistance at 70 °C for 5 min per cycle. Fmoc deprotection was carried out using 20% 4-methylpiperidine in AcOEt at 40 °C for 5 min per cycle, which was likewise delivered and removed by the automated system. All coupling, washing, and deprotection steps proceeded in a fully automated manner. Following cleavage and work-up were described in Section S3.1 of the SI, the crude peptide was analyzed by HPLC (220 nm), showing a dominant peak with a purity of 98%. ESI-MS (TOF) *m*/*z*: 555.2312 [M + H]^+^ (calcd. for C_28_H_39_N_6_O_6_, 555.2931).

#### 4.4.2 Substance P

Fully automated aqueous SPPS of Substance P (H-RPKPQQFFGLM-NH_2_) was carried out on an Initiator+ Alstra peptide synthesizer according to the programmed workflow in Scheme 2. The synthesis was designed to apply position-dependent coupling protocols to ensure robust elongation of the peptide chain. Double coupling cycle was used for the *C*-terminal Met residue, single coupling cycles for the subsequent four residues, and double coupling cycles for the six *N*-terminal residues (Table S3). All coupling steps were executed by automated robotic delivery of aqueous nanoassemblies of Fmoc-protected amino acids and aqueous reagent solutions into the reaction vessel. Fmoc deprotection was performed using EtOAc and 4-methylpiperidine, which were sequentially delivered to and removed from the reaction vessel by the automated system. Deprotection, washing, and reagent exchange steps proceeded in a fully unattended manner throughout the synthesis. Following cleavage and work-up were described in Section S3.1 of the SI, the crude peptide was analyzed by HPLC (220 nm), showing a dominant peak with a purity of 81%. ESI-MS (TOF) *m*/*z*: 1347.7373 [M + H]^+^ (calcd. for C_63_H_98_N_19_O_13_S, 1347.7288).

#### 4.4.3 GLP-1 (1–37)

Fully automated aqueous SPPS of GLP-1 (1–37) (H-HDEFERHAEGTFTSDVSSYLEGQAAKEFIAWLVKGRG-NH2) was synthesized on an Initiator+ Alstra system using the programmed workflow in Scheme 2. To address sequence-dependent steric demands during chain elongation, position-dependent coupling protocols were implemented. Sterically hindered residues were subjected to double coupling cycles, while all other residues were coupled using single cycles (Table S4 and Section S2.1 in the SI). For His residues, DMT-MM was employed as the coupling reagent. All coupling steps were executed by automated robotic delivery of aqueous nanoassemblies of Fmoc-protected amino acids and aqueous reagent solutions into the reaction vessel. Fmoc deprotection, washing, and reagent exchange steps were performed under full robotic control without manual intervention, and the entire synthesis proceeded in a fully automated manner under the programmed conditions. Following cleavage and work-up were described in Section S3.1 of the SI, the crude peptide was analyzed by HPLC (220 nm), showing a major peak at a retention time of 20.6 min with a calculated purity of 16%. Purification by preparative HPLC afforded under 1 mg of the pure peptide as the TFA salt. Due to partial co-elution with closely related impurities, repeated HPLC purification was required, resulting in a reduced isolated yield. ESI-MS (TOF): *m*/*z* 1390.2165 ([M+3H]^3+^) and *m*/*z* 1042.9207 ([M+4H]^4+^) which corresponds to C_186_H_276_N_52_O_58_ (calculated monoisotopic mass: 4166.0246).

## Author contributions

K. Hojo: conceptualization, investigation, writing – original draft, and funding acquisition. M. Kishimoto: investigation including optimization studies for automated aqueous SPPS and analytical measurements. A. Nagai: investigation including studies for automated aqueous SPPS and analytical measurements. C. Rentier: Automated peptide synthesis optimization, scientific and technical input, and review & editing. A. Mehrotra: Design of the automated synthesis platform, scientific and technical input, and review & editing. K. Hioki: DLS analysis, analytical measurements, and review & editing, and funding acquisition. M. Kunishima: supervision, project administration, review & editing, and funding acquisition.

## Conflicts of interest

C. Rentier is an employee of Biotage Japan Ltd A. Mehrotra is an employee of Biotage Sweden AB. The authors declare that related patent applications (WO2024-143052 and JP Application No. 2025-149977) exist concerning the aqueous nanoassembly methodology. K. Hojo is named as an inventor on these applications.

## Supplementary Material

RA-016-D6RA06336E-s001

## Data Availability

All data supporting the findings of this study are available within the article and its supplementary information (SI). Supplementary information: particle size of nanoassemblies (DLS analysis data) and peptide characterization (ESI-Q-TOF-MS spectra and analytical HPLC profiles) (PDF). See DOI: https://doi.org/10.1039/d6ra06336e.
