# Supplementary material for: Controlled aqueous nanoassembly-driven fully automated Fmoc solid-phase peptide synthesis (SPPS) with in-water coupling
Source: RSC Adv. 2026 Sep 10;16(43):53859–70. doi: 10.1039/d6ra06336e (PMC13559539; doi:10.1039/d6ra06336e)
Supplement: Supplementary file 1 [file RA-016-D6RA06336E-s001.pdf]

Supplementary Information (SI) for RSC Advances.
This journal is © The Royal Society of Chemistry 2026

Supplementary Information

Controlled Aqueous Nanoassembly-Driven Fully Automated Fmoc Solid-Phase Peptide Synthesis (SPPS) with In-Water Coupling

Keiko Hojo*[a,b], Mutsumi Kishimoto[a], Ayana Nagai[a], Cédric Rentier[c], Amit Mehrotra[d], Kazuhito Hioki[a,b], and Munetaka Kunishima*[a,b]

[a]*Faculty of Pharmaceutical Sciences, Kobe Gakuin University, Kobe 650-8586, Japan. E-mail: hojo@pharm.kobegakuin.ac.jp, kunisima@pharm.kobegakuin.ac.jp*

[b]*Cooperative Research Center of Life Sciences, Kobe Gakuin University, Kobe 650-8586, Japan.*

[c]*Biotage Japan Ltd., Tokyo 136-0071, Japan.*

[d]*Biotage Sweden AB, Uppsala 753 18, Sweden.*

Table of Contents

S1. DLS Analysis of Nanoassemblies

S1.1. DLS Measurement and Data Interpretation ---------- 4

Table S1. Glossary (Japanese–English) of DLS Output Terms ---------- 4

S1.2. Representative Examples ---------- 6

S1.2.1. Fmoc-Val-OH Nanoassemblies: A Relatively Narrow Size Distribution ---------- 6

Figure S1. DLS Analysis of Fmoc-Val-OH Nanoassemblies ---------- 6

S1.2.2. Fmoc-Phe-OH Nanoassemblies: Gradual Formation of Nanoassemblies---------- 7

Figure S2. DLS Analysis of Fmoc-Phe-OH Nanoassemblies ---------- 7

S1.3. DLS Analysis of Additional Fmoc-Amino Acid Nanoassemblies ---------- 8

Figure S3. DLS Analysis of Fmoc-Ala-OH Nanoassemblies ---------- 8

Figure S4. DLS Analysis of Fmoc-Arg(Pbf)-OH Nanoassemblies ---------- 9

Figure S5. DLS Analysis of Fmoc-Asn(Trt)-OH Nanoassemblies ----------10

Figure S6. DLS Analysis of Fmoc-Asp(OtBu)-OH Nanoassemblies ----------11

Figure S7. DLS Analysis of Fmoc-Cys(Acm)-OH Nanoassemblies ----------12

Figure S8. DLS Analysis of Fmoc-Cys(Trt)-OH Nanoassemblies ----------13

Figure S9. DLS Analysis of Fmoc-Gln(Trt)-OH Nanoassemblies ----------------------------------14

Figure S10. DLS Analysis of Fmoc-Glu(OtBu)-OH Nanoassemblies -------------------------------15

Figure S11. DLS Analysis of Fmoc-Gly-OH Nanoassemblies ---------------------------------------16

Figure S12. DLS Analysis of Fmoc-His(Boc)-OH Nanoassemblies ---------------------------------17

Figure S13. DLS Analysis of Fmoc-Ile-OH Nanoassemblies ---18

Figure S14. DLS Analysis of Fmoc-Leu-OH Nanoassemblies ---------------------------------------19

Figure S15. DLS Analysis of Fmoc-Lys(Boc)-OH Nanoassemblies ---------------------------------20

Figure S16. DLS Analysis of Fmoc-Met-OH Nanoassemblies ---------------------------------------21

Figure S17. DLS Analysis of Fmoc-Pro-OH Nanoassemblies ---------------------------------------22

Figure S18. DLS Analysis of Fmoc-Ser(tBu)-OH Nanoassemblies ---------------------------------23

Figure S19. DLS Analysis of Fmoc-Thr(tBu)-OH Nanoassemblies ---------------------------------24

Figure S20. DLS Analysis of Fmoc-Trp(Boc)-OH Nanoassemblies ---------------------------------25

Figure S21. DLS Analysis of Fmoc-Tyr(tBu)-OH Nanoassemblies ---------------------------------26

S1.4. Particle Size of Nanoassemblies ---27

Table S2. Mode Diameter (Intensity-Weighted) of Fmoc-Amino Acid Nanoassemblies ----------27

Table S3. Mode Diameter (Number-Weighted) of Fmoc-Amino Acid Nanoassemblies -----------28

S2. Coupling Cycle Design for Fully Automated Aqueous SPPS of Substance P and GLP-1 (1-37)

S2.1. Guidelines for Determining the Number of Coupling Cycles --------------------------------------29

S2.2. The Number of Coupling Cycles ---29

Table S4. Coupling Cycles Used for Fully Automated Aqueous SPPS of Substance P ------------29

Table S5. Coupling Cycles Used for Fully Automated Aqueous SPPS of GLP-1 (1-37) ----------29

S3. Method of Cleavage and Global Deprotection

S3.1. Cleavage and Global Deprotection of H-Protected Peptide-Rink Amide TentaGel Resin, Example Peptide: GLP-1 (1-37) --30

S4. Characterization of Peptides

S4.1. HPLC Analysis ---30

S4.2. Mass Analysis --30

S4.3. Leu-Enkephalin Amide ---31

Figure S22. Analytical HPLC Profile and ESI-MS Spectrum of Leu-Enkephalin Amide Synthesized by Fully Automated Aqueous SPPS Using TBTU--------------------------------------31

Figure S23. Analytical HPLC Profile of Leu-Enkephalin Amide Synthesized by Fully Automated Aqueous SPPS Using DMT-MM -- 32
Figure S24. Analytical HPLC Profile and ESI-MS Spectrum of Leu-Enkephalin Amide Synthesized by Conventional Fmoc-SPPS in DMF -- 33
S4.4. Substance P --- 34
Figure S25. Analytical HPLC Profile of Substance P Synthesized by Fully Automated Aqueous SPPS Using TBTU According to Scheme 1 --- 34
Figure S26. Analytical HPLC Profile and ESI-MS Spectrum of Substance P Synthesized by Fully Automated Aqueous SPPS Using TBTU According to Scheme 2 -------------------------------- 35
Figure S27. Analytical HPLC Profile and ESI-MS Spectrum of Substance P Synthesized by Conventional Fmoc-SPPS in DMF --- 36
S4.5. GLP-1 (1-37) -- 37
Figure S28. Analytical HPLC Profile and ESI-MS Spectrum of GLP-1 (1-37) Synthesized by Fully Automated Aqueous SPPS Using TBTU-- 37
Figure S29. Co-Injection Analysis for Identification of His-Deletion Products in Crude GLP-1(1-37). -- 38
Figure S30. Analytical HPLC Profile and ESI-MS Spectrum of GLP-1 (1-37) Synthesized by Fully Automated Aqueous SPPS Using TBTU with DMT-MM at His Position ------------------------ 39
Figure S31. Analytical HPLC Profile and ESI-MS Spectrum of GLP-1 (1–37) Synthesized by Conventional Fmoc-SPPS in DMF --- 40

S5. PMI

S5.1. Calculation of Process Mass Intensity (PMI) --- 41
S5.2. PMI Comparison Between Aqueous SPPS (ASPPS) and Conventional Fmoc-SPPS in DMF-42

S6. Coupling Reagents

S6.1. Structure of Coupling Reagents Used in This Study -- 43

S7. References -- 44

S1. Dynamic Light Scattering (DLS) Analysis[1, 2)]

S1.1. DLS Measurement and Data Interpretation

Dynamic light scattering (DLS) measurements were performed following previously reported procedures.[1-3] DLS analysis was used to evaluate the formation and relative evolution of nanoassembly states under different conditions. The hydrodynamic diameter distributions, Z-average diameter, and polydispersity index (PDI) were obtained from the autocorrelation function. Raw DLS output data presented in this section (Figures S1–S21) were exported from the instrument software configured in Japanese. A glossary of the Japanese terms appearing in the figures and their English equivalents is provided in Table S1 below.

Table S1. Glossary (Japanese–English) of DLS Output Terms

Japanese	English
結果	Results
Z 平均 (d.nm)	Z-average (d.nm)
PdI	PdI (Polydispersity Index)
インターセプト	Intercept
結果品質	Result quality
直径 (nm)	Diameter (nm)
幅 (nm)	Width (nm)
% 散乱強度	% Scattering intensity
% 個数	% Number
散乱強度別粒度分布	Particle size distribution by scattering intensity
個数別粒度分布	Particle size distribution by number
散乱強度 (Percent)	Scattering intensity (%)
個数 (Percent)	Number (Percent)
サイズ (d.nm)	Size (d.nm)
レコード	Record

Because the present systems are heterogeneous and may contain coexisting nanoassemblies, intermediate aggregates, and molecularly dispersed species, careful interpretation of the size distributions was required. In particular, large assemblies can disproportionately influence the scattering signal because scattering intensity strongly depends on particle diameter (intensity $\propto d^6$).[4] Therefore, both intensity- and number-weighted size distributions were evaluated as complementary information for interpretation of particle populations.

The Z-average diameter and PDI were calculated using the cumulant method. PDI values ≤ 0.3 were considered indicative of relatively narrow size distributions, whereas PDI values > 0.3 suggest increased heterogeneity and/or deviation from the assumptions of the cumulant approximation.[5,6] In highly polydisperse systems, the Z-average value was treated as a supplementary parameter rather than a representative particle diameter.

For interpretation of particle size distributions, the mode diameter obtained from the intensity-weighted distribution was primarily used to evaluate the overall scattering behavior and the formation of nanoassemblies (Table S2). In parallel, number-weighted distributions were analyzed as complementary information to evaluate the presence of smaller particle populations (Table S3).

In this study, the intensity-weighted distributions are shown in the main text to illustrate the collective behavior of nanoassemblies, whereas the number-weighted distributions are included to support interpretation of particle populations. These data were interpreted in a complementary manner to characterize both the overall assembly state and the coexistence of smaller species. The primary objective of this study is to demonstrate the formation and evolution of nanoassemblies rather than to quantitatively resolve all distribution-dependent size variations.

S1.2. Representative Examples

S1.2.1. Fmoc-Val-OH Nanoassemblies: A Relatively Narrow Size Distribution

A: Two-Component Nanoassemblies

(a) Intensity-weighted distribution

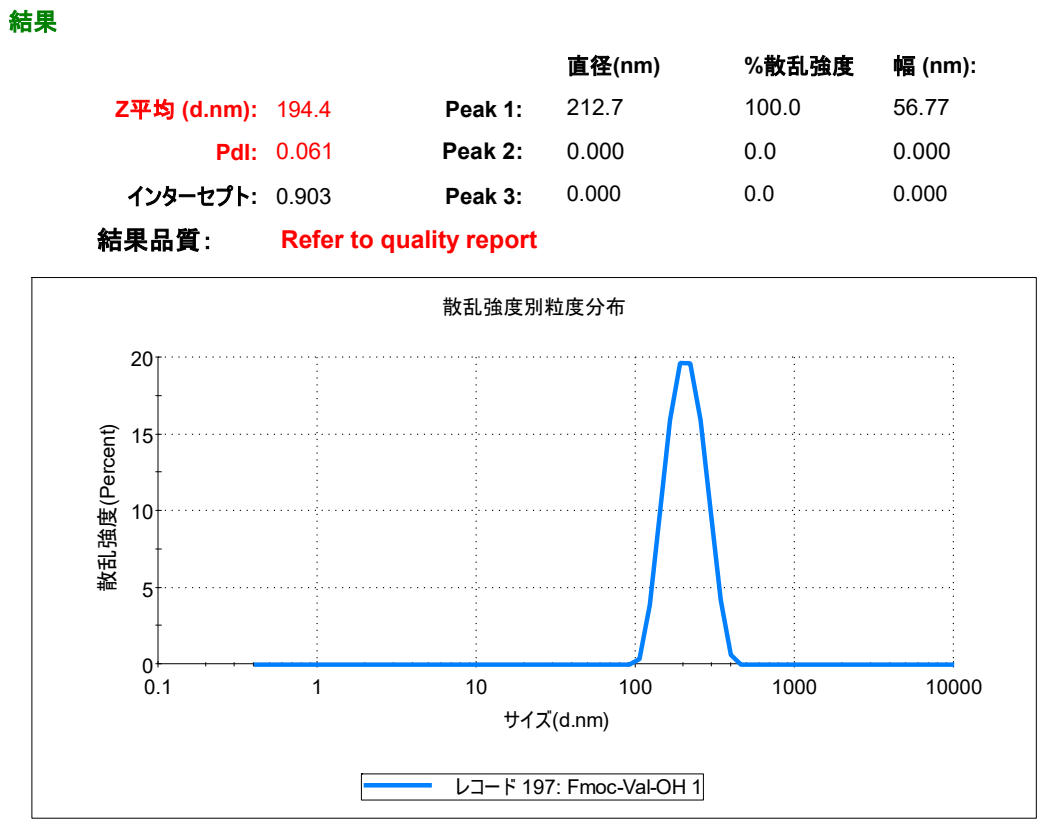

(b) Number-weighted distribution

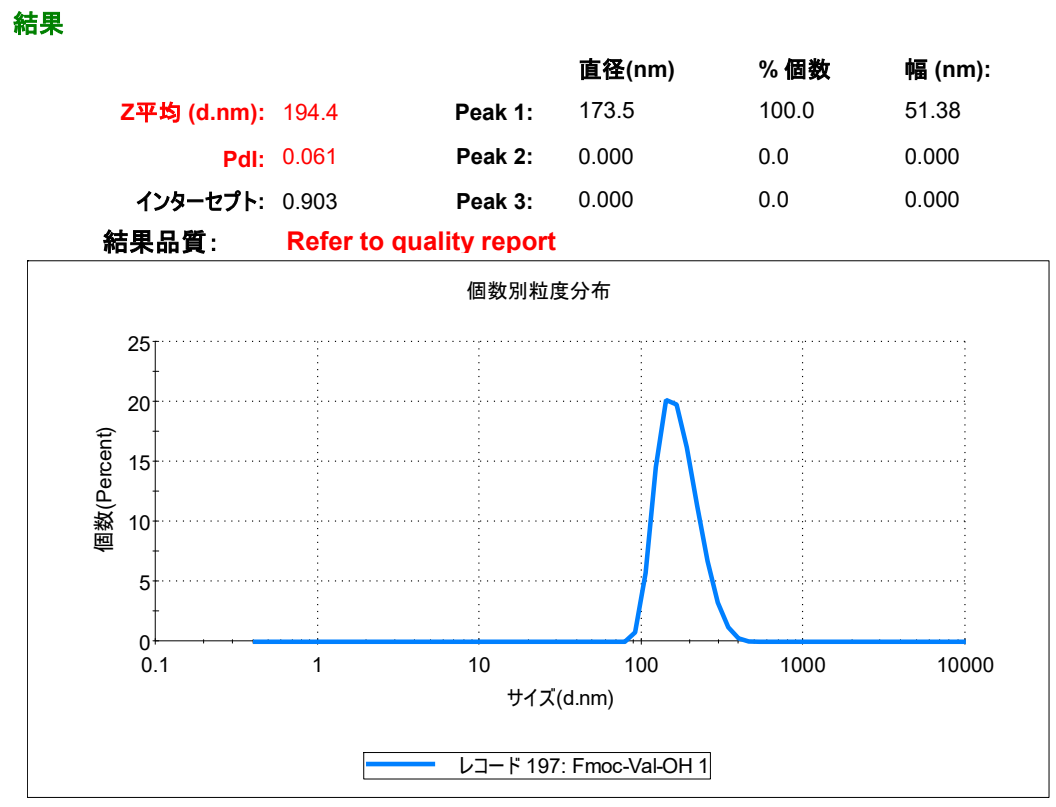

B: Three-Component Nanoassemblies

(a) Intensity-weighted distribution

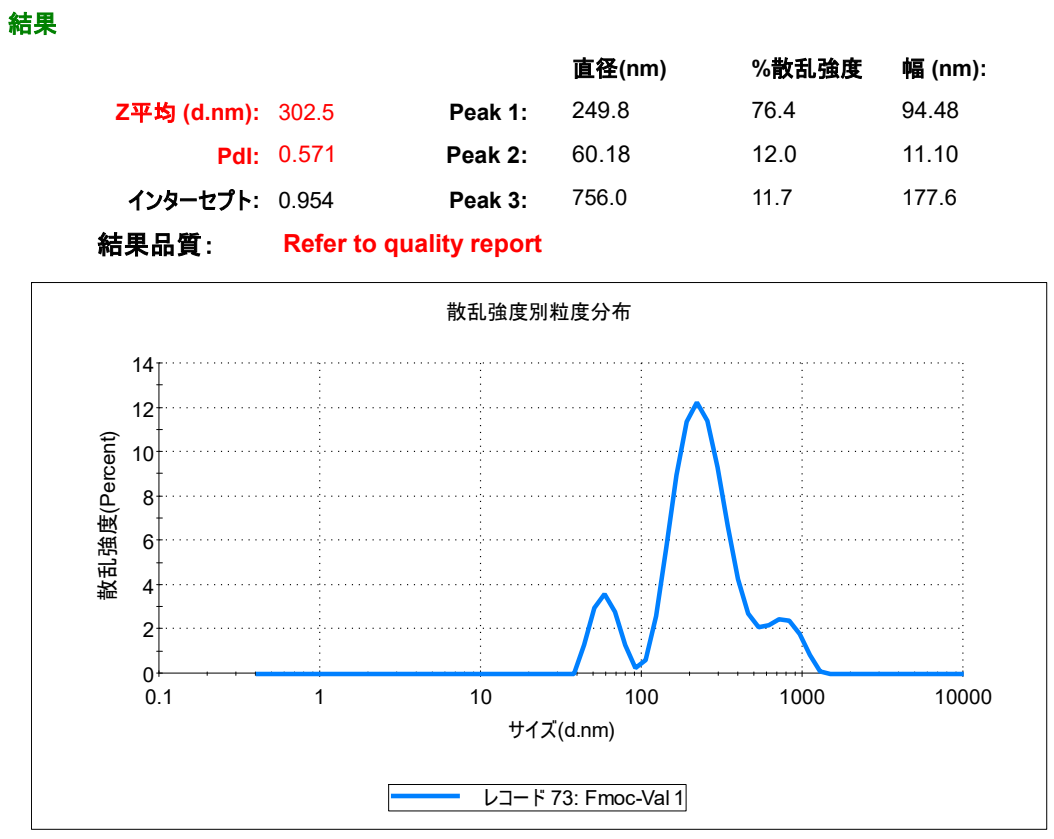

(b) Number-weighted distribution

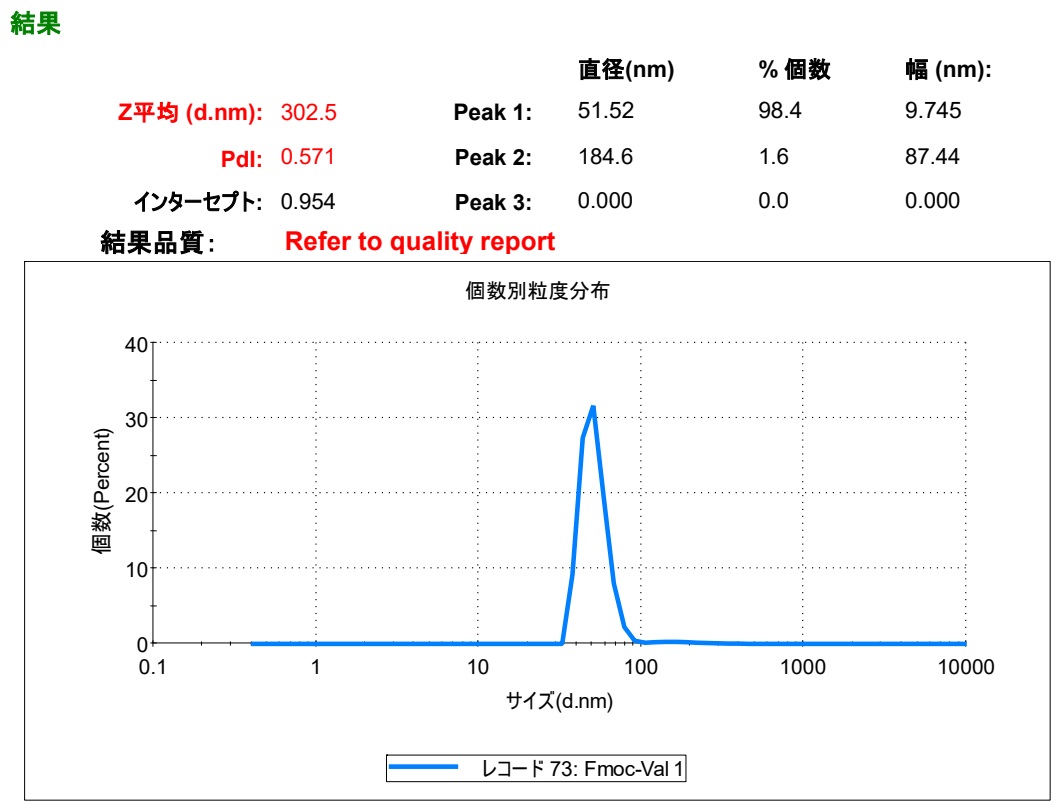

Figure S1. DLS Analysis of Fmoc-Val-OH Nanoassemblies.

(Japanese labels appearing in the DLS output figures are defined in Table S1.)

A: Two-Component Nanoassemblies (Fmoc-Val-OH and DIEA).

(a) The intensity-weighted distribution exhibited a relatively narrow size distribution with a dominant population centered at approximately 212 nm, consistent with nanoassembly formation.

(b) The corresponding number-weighted distribution showed a predominant particle population in a similar size range.

B: Three-Component Nanoassemblies (Fmoc-Val-OH, DIEA and TBTU).

(a) The intensity-weighted distribution displayed multiple particle populations, suggesting the coexistence of different assembly states.

(b) The number-weighted distribution revealed a predominant particle population together with minor smaller species.

S1.2.2. Fmoc-Phe-OH Nanoassemblies: Gradual Formation of Nanoassemblies

A: Two-Component Nanoassemblies

(a) Intensity-weighted distribution

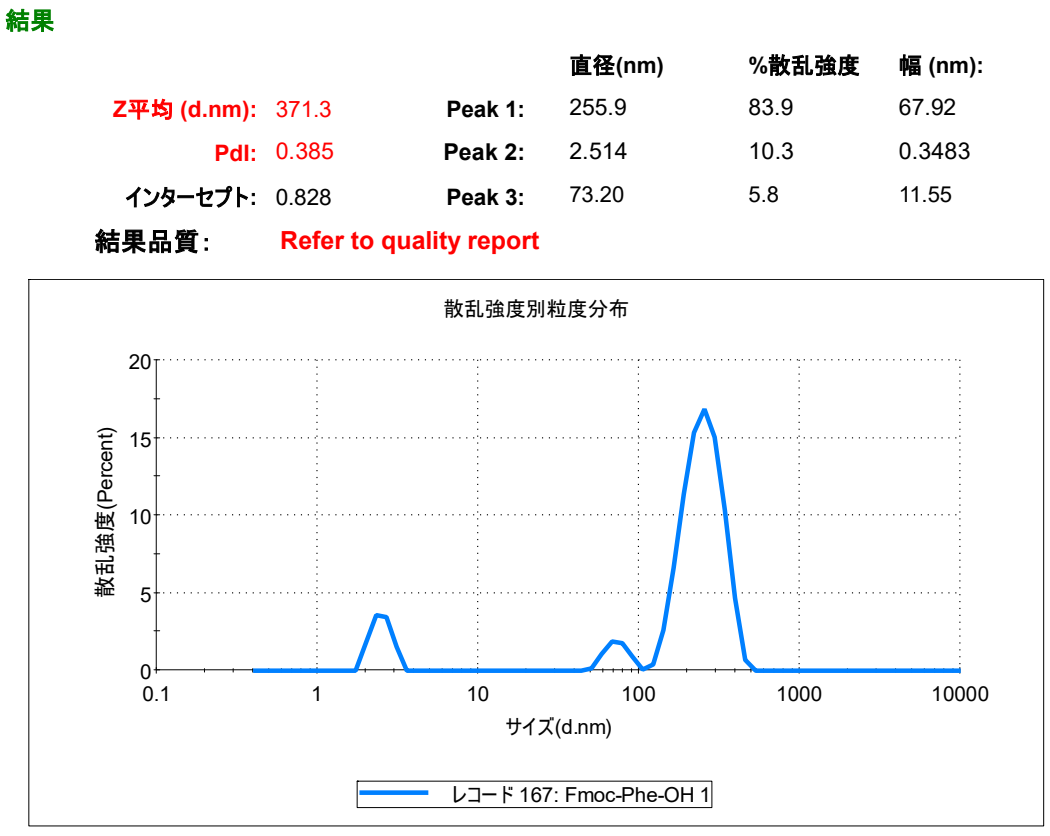

(b) Number-weighted distribution

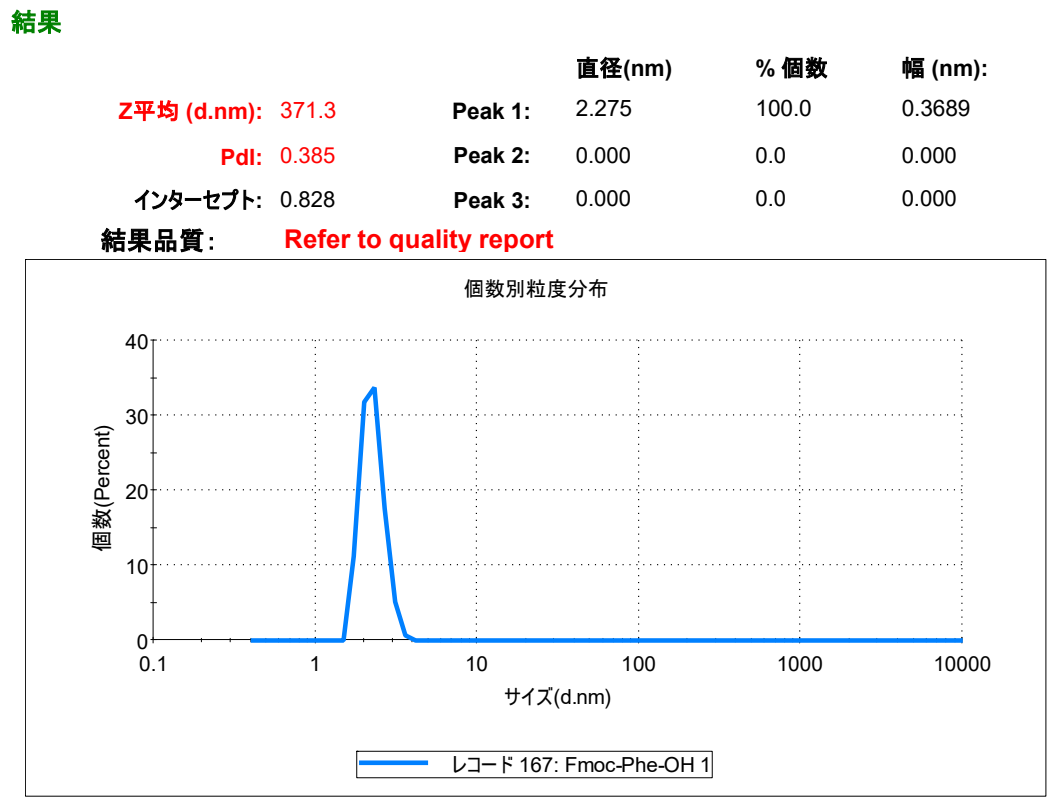

B: Three-Component Nanoassemblies

(a) Intensity-weighted distribution

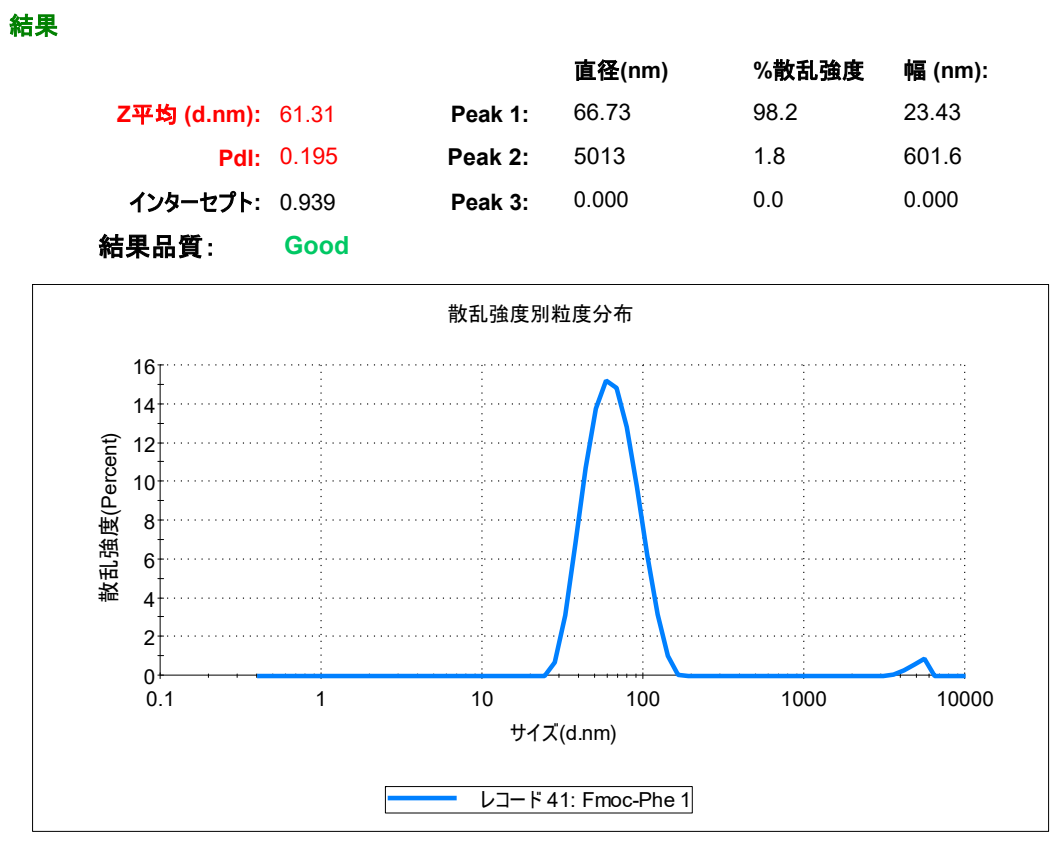

(b) Number-weighted distribution

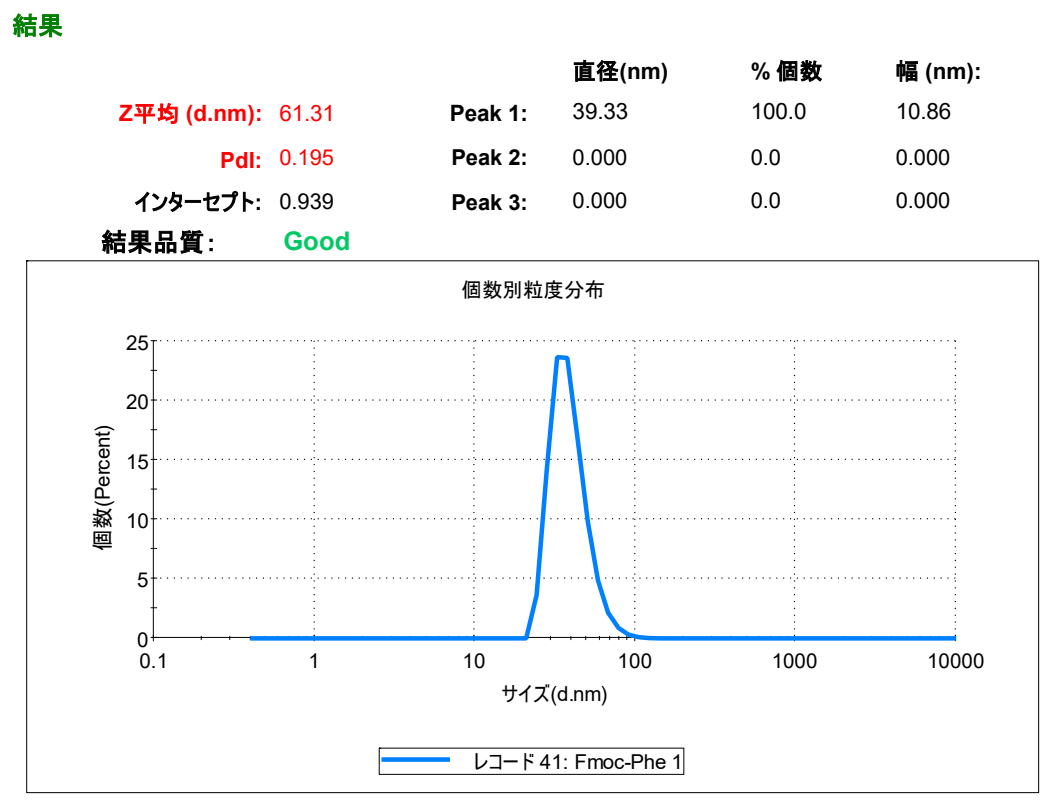

Figure S2. DLS Analysis of Fmoc-Phe-OH Nanoassemblies.

(Japanese labels appearing in the DLS output figures are defined in Table S1.)

A: Two-Component Nanoassemblies (Fmoc-Phe-OH and DIEA).

(a) The intensity-weighted distribution showed multiple particle populations, including a dominant population in the hundreds-of-nanometer range together with smaller species, indicating a heterogeneous assembly state.

(b) The corresponding number-weighted distribution revealed a predominant small-particle population, consistent with the coexistence of smaller dispersed species and larger nanoassemblies.

B: Three-Component Nanoassemblies (Fmoc-Phe-OH, DIEA, and TBTU).

(a) Upon introduction of the third component, the intensity-weighted distribution converged toward a dominant single peak, indicating formation of a more uniform nanoassembly population.

(b) The number-weighted distribution also showed a predominant particle population with reduced contribution from smaller species.

S1.3. DLS Analysis of Additional Fmoc-Amino Acid Nanoassemblies

A: Two-Component Nanoassemblies

(a) Intensity-weighted distribution

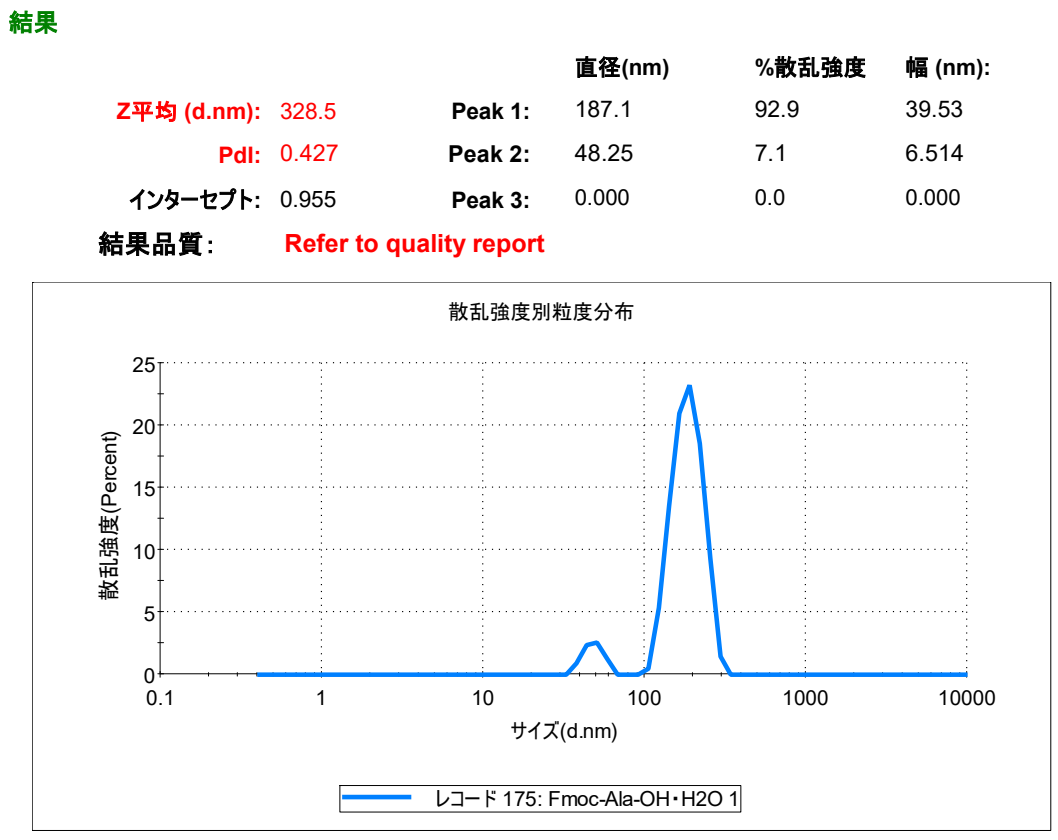

(b) Number-weighted distribution

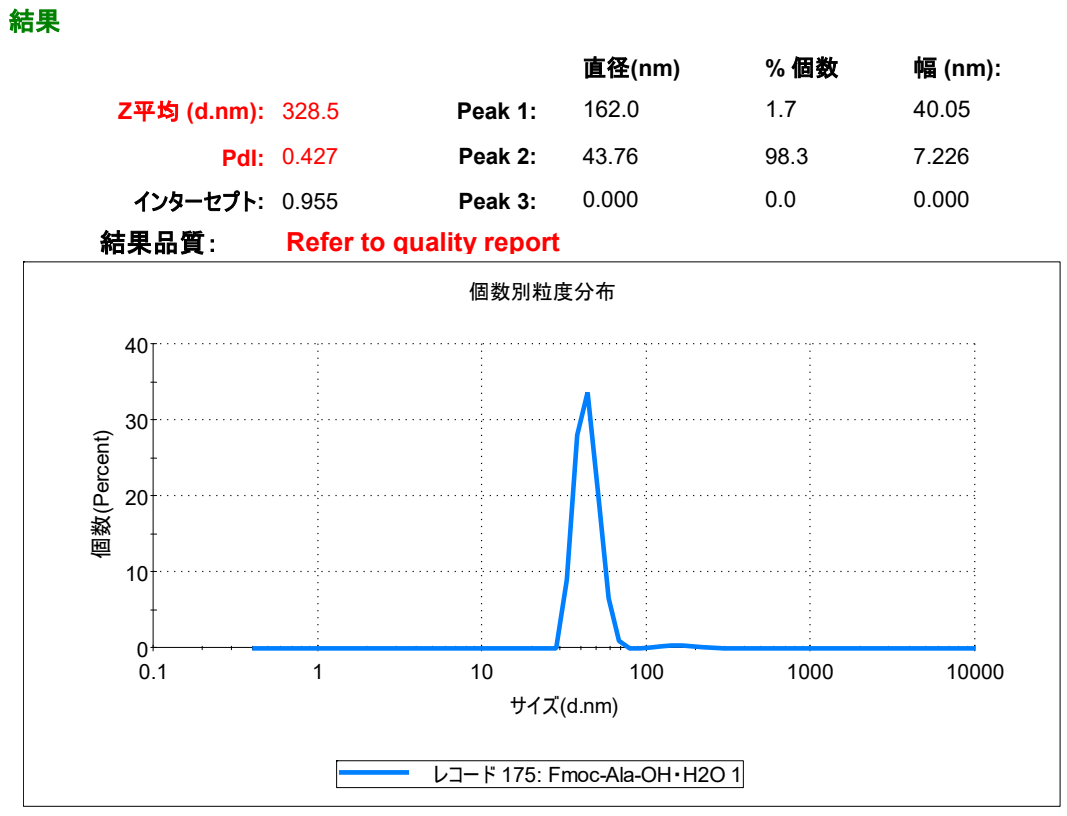

B: Three-Component Nanoassemblies

(a) Intensity-weighted distribution

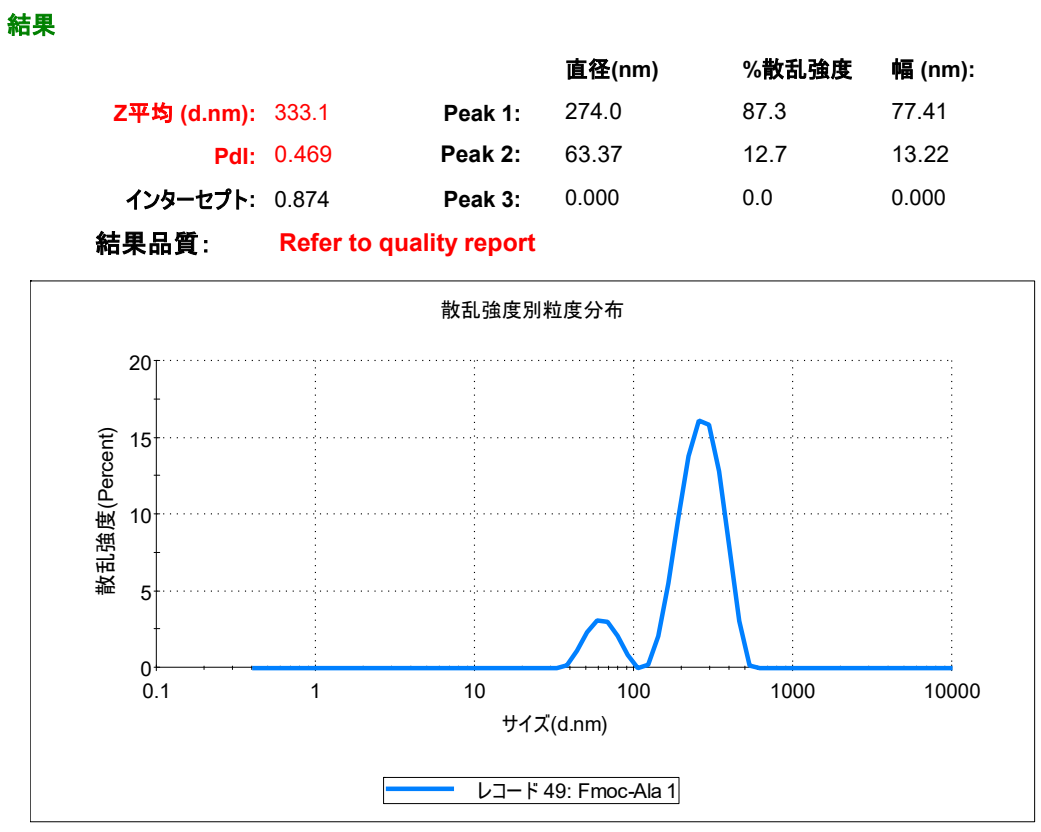

(b) Number-weighted distribution

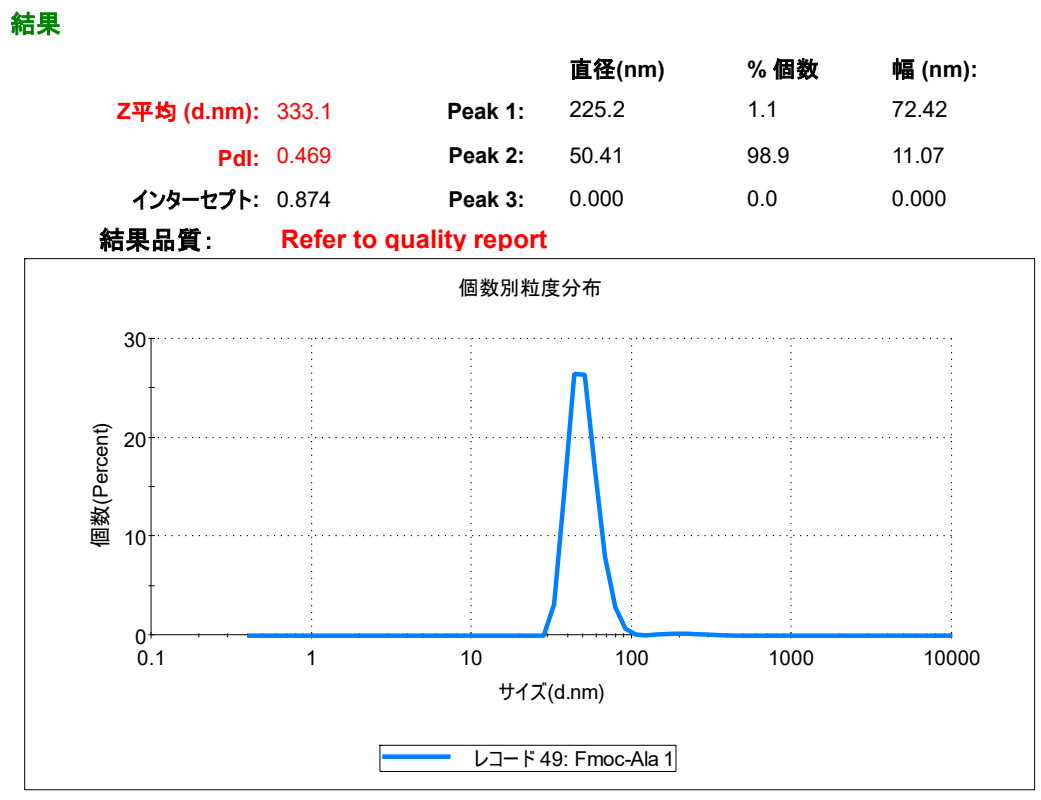

Figure S3. DLS Analysis of Fmoc-Ala-OH Nanoassemblies.

(Japanese labels appearing in the DLS output figures are defined in Table S1.)

A: Two-Component Nanoassemblies (Fmoc-Ala-OH and DIEA).

(a) The intensity-weighted distribution; (b) The corresponding number-weighted distribution.

B: Three-Component Nanoassemblies (Fmoc-Ala-OH, DIEA and TBTU).

(a) The intensity-weighted distribution; (b) The number-weighted distribution.

A: Two-Component Nanoassemblies

(a) Intensity-weighted distribution

結果

			直径(nm)	%散乱強度	幅 (nm):
Z平均 (d.nm):	344.8	Peak 1:	267.0	84.7	110.0
PdI:	0.365	Peak 2:	2.216	15.3	0.3045
インターセプト:	0.920	Peak 3:	0.000	0.0	0.000
結果品質 :	Refer to quality report				

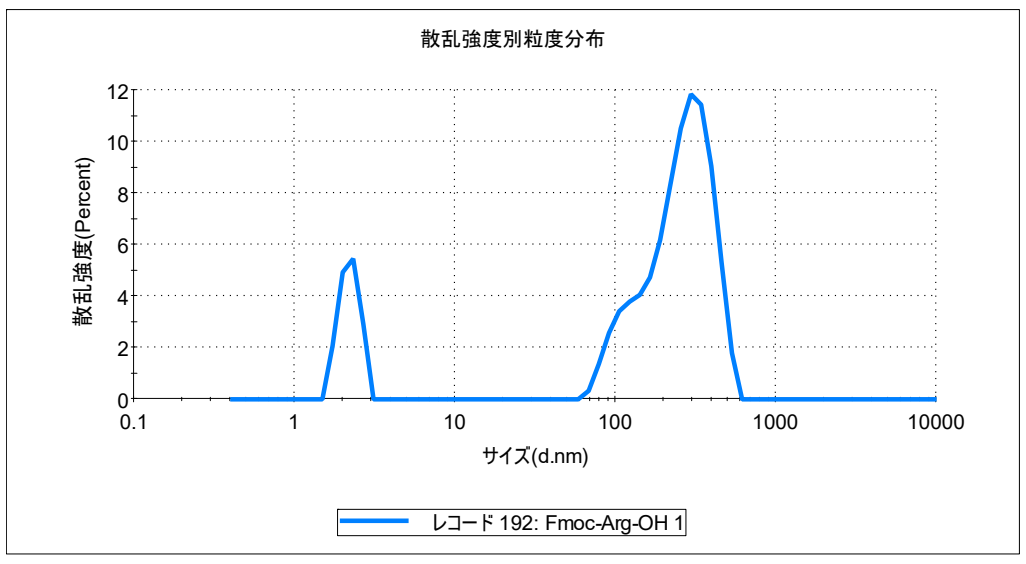

(b) Number-weighted distribution

結果

			直径(nm)	% 個数	幅 (nm):
Z平均 (d.nm):	344.8	Peak 1:	1.997	100.0	0.3315
PdI:	0.365	Peak 2:	0.000	0.0	0.000
インターセプト:	0.920	Peak 3:	0.000	0.0	0.000
結果品質 :	Refer to quality report				

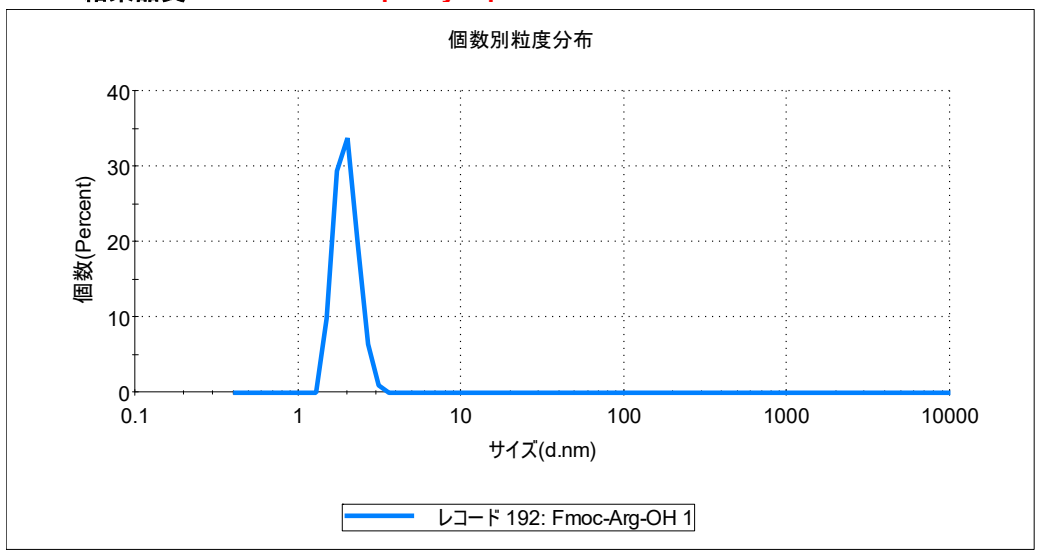

B: Three-Component Nanoassemblies

(a) Intensity-weighted distribution

結果

			直径(nm)	%散乱強度	幅 (nm):
Z平均 (d.nm):	202.0	Peak 1:	217.3	100.0	57.15
PdI:	0.060	Peak 2:	0.000	0.0	0.000
インターセプト:	0.928	Peak 3:	0.000	0.0	0.000
結果品質 :	Good				

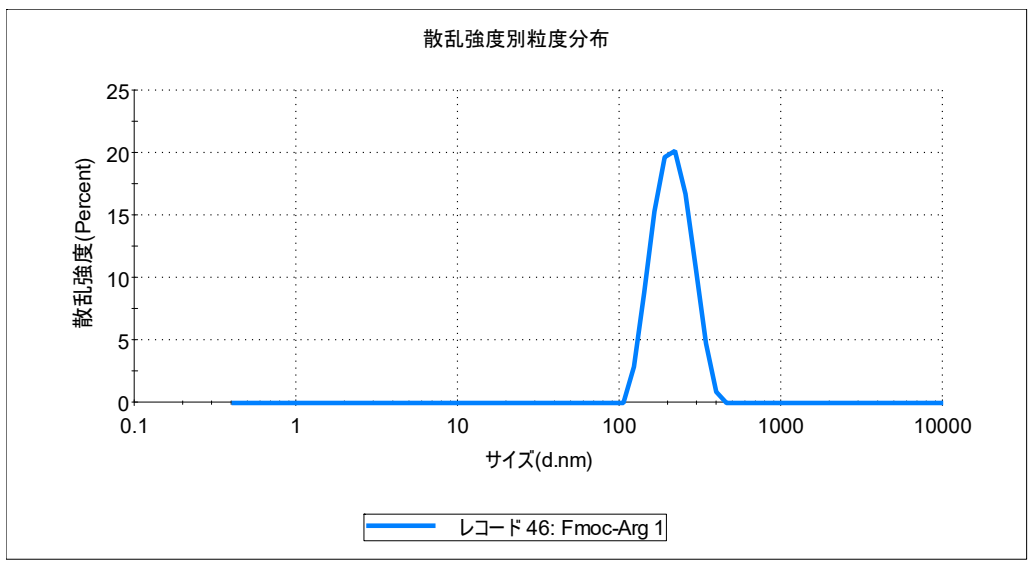

(b) Number-weighted distribution

結果

			直径(nm)	% 個数	幅 (nm):
Z平均 (d.nm):	202.0	Peak 1:	180.2	100.0	51.90
PdI:	0.060	Peak 2:	0.000	0.0	0.000
インターセプト:	0.928	Peak 3:	0.000	0.0	0.000
結果品質 :	Good				

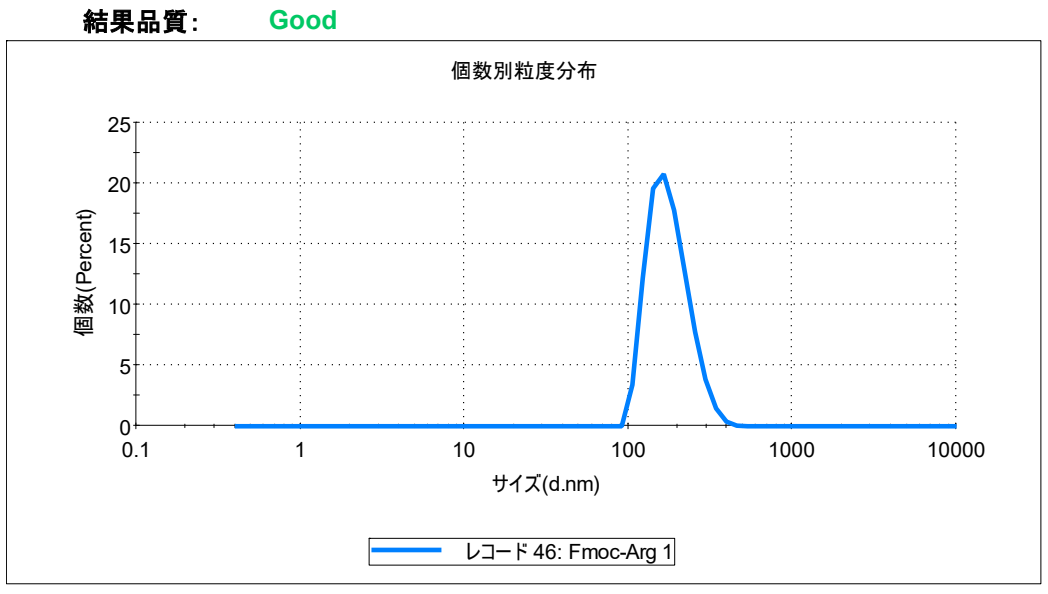

Figure S4. DLS Analysis of Fmoc-Arg(Pbf)-OH Nanoassemblies.

(Japanese labels appearing in the DLS output figures are defined in Table S1.)

A: Two-Component Nanoassemblies (Fmoc-Arg(Pbf)-OH and DIEA).

(a) The intensity-weighted distribution; (b) The corresponding number-weighted distribution.

B: Three-Component Nanoassemblies (Fmoc-Arg(Pbf)-OH, DIEA and TBTU).

(a) The intensity-weighted distribution; (b) The number-weighted distribution.

A: Two-Component Nanoassemblies

(a) Intensity-weighted distribution

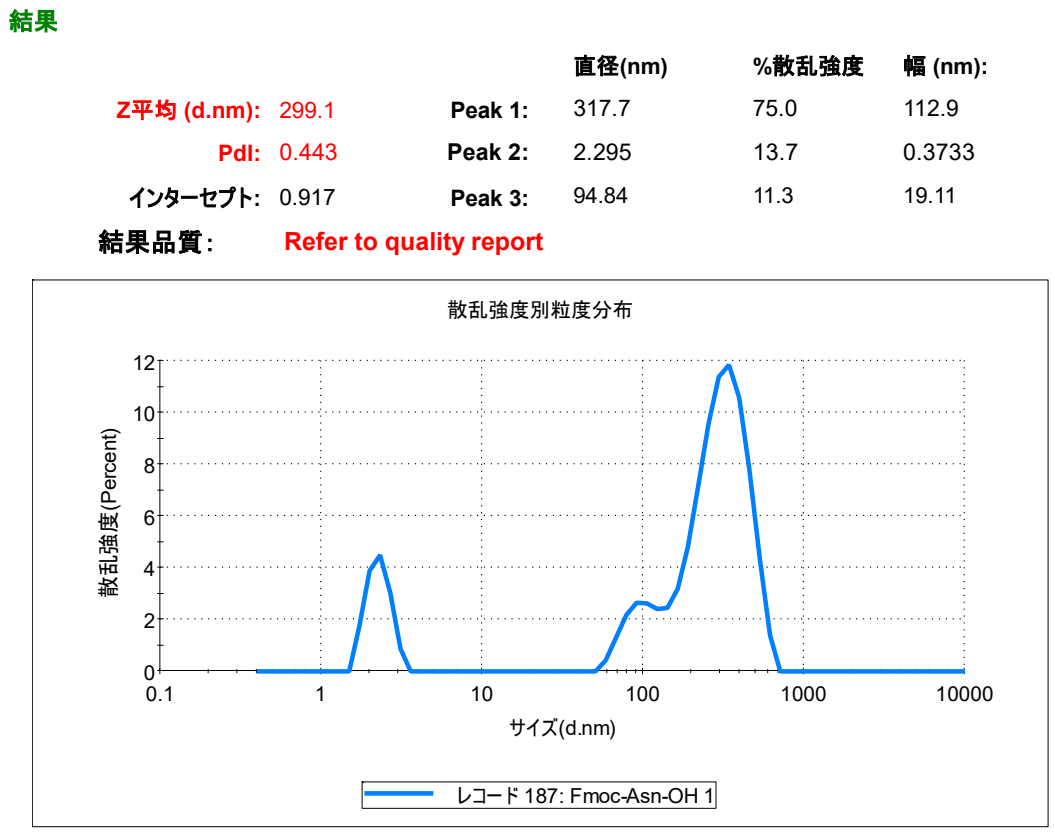

(b) Number-weighted distribution

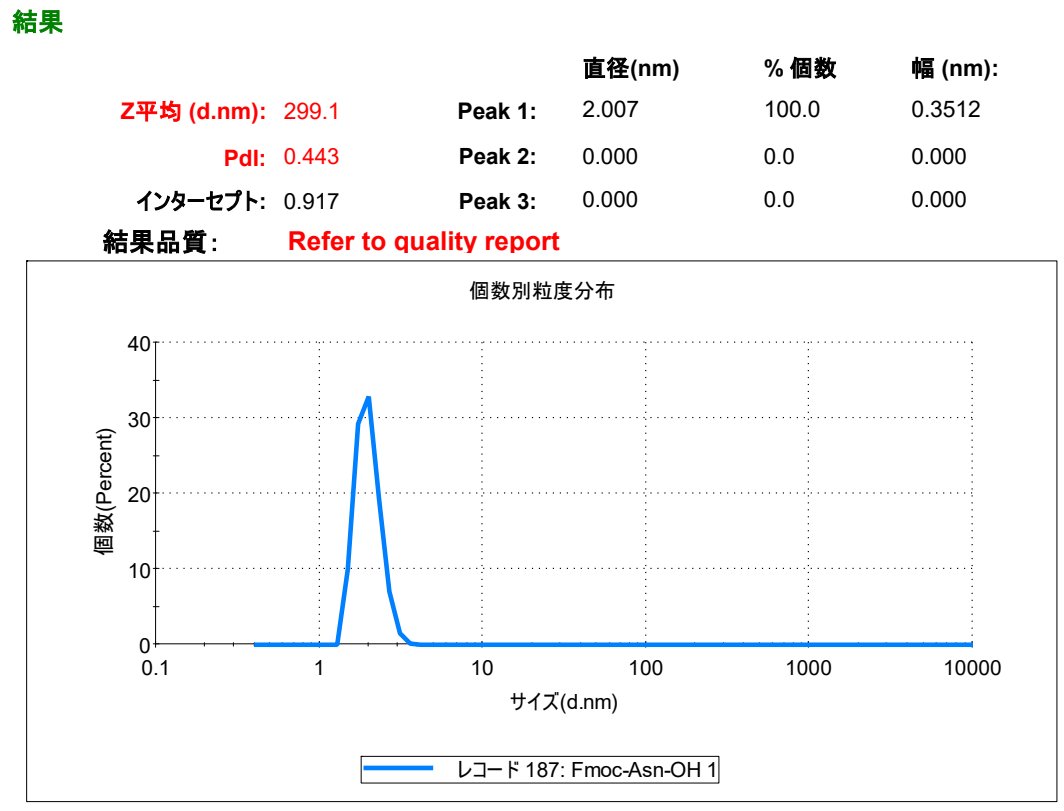

B: Three-Component Nanoassemblies

(a) Intensity-weighted distribution

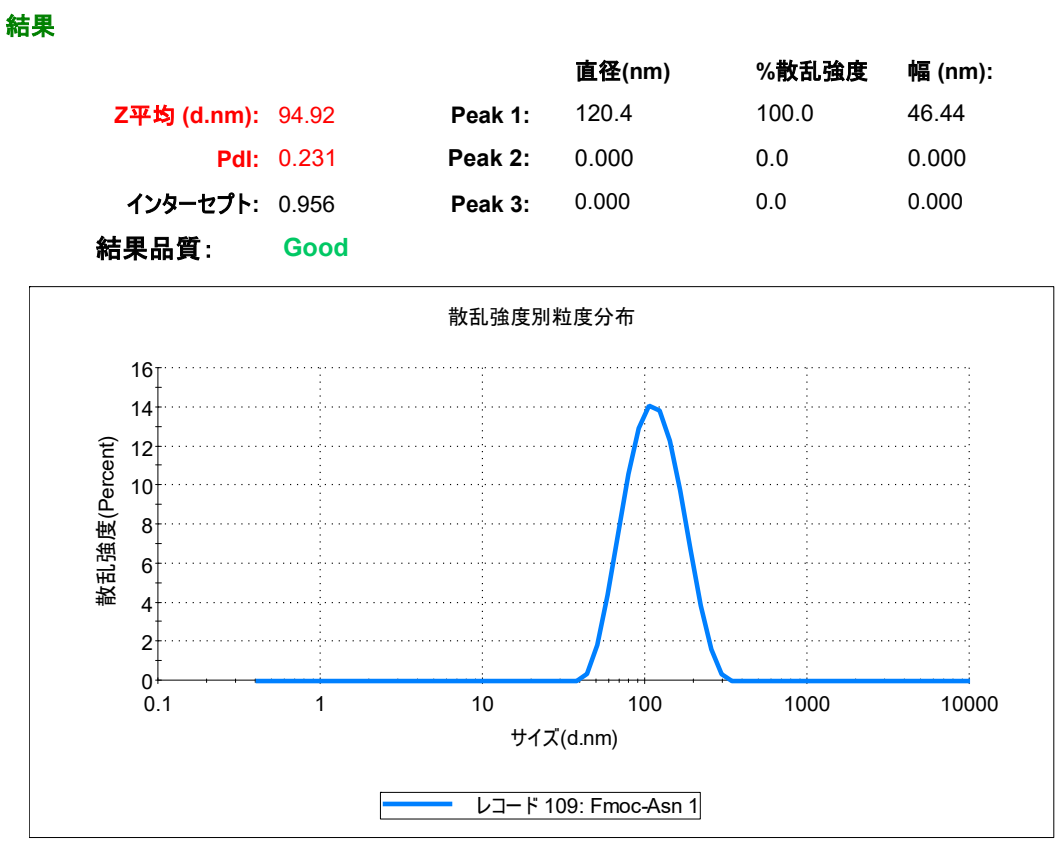

(b) Number-weighted distribution

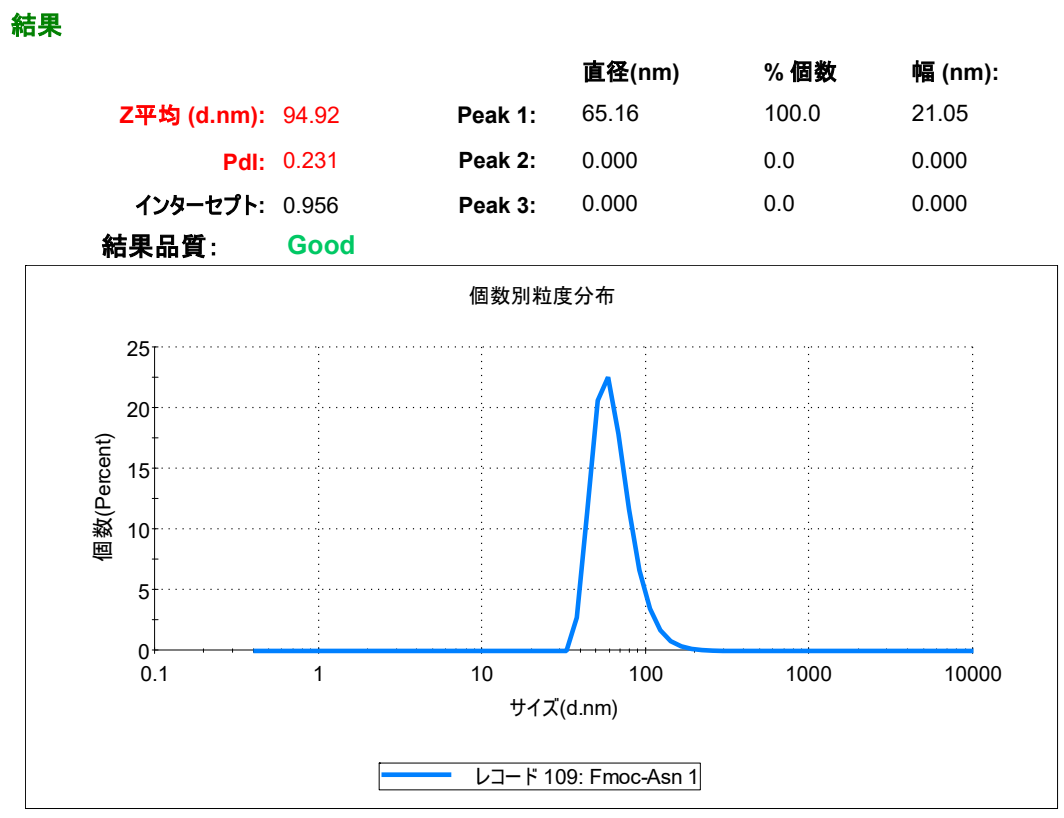

Figure S5. DLS Analysis of Fmoc-Asn(Trt)-OH Nanoassemblies.

(Japanese labels appearing in the DLS output figures are defined in Table S1.)

A: Two-Component Nanoassemblies (Fmoc-Asn(Trt)-OH and DIEA).

(a) The intensity-weighted distribution; (b) The corresponding number-weighted distribution.

B: Three-Component Nanoassemblies (Fmoc-Asn(Trt)-OH, DIEA and TBTU).

(a) The intensity-weighted distribution; (b) The number-weighted distribution.

A: Two-Component Nanoassemblies

(a) Intensity-weighted distribution

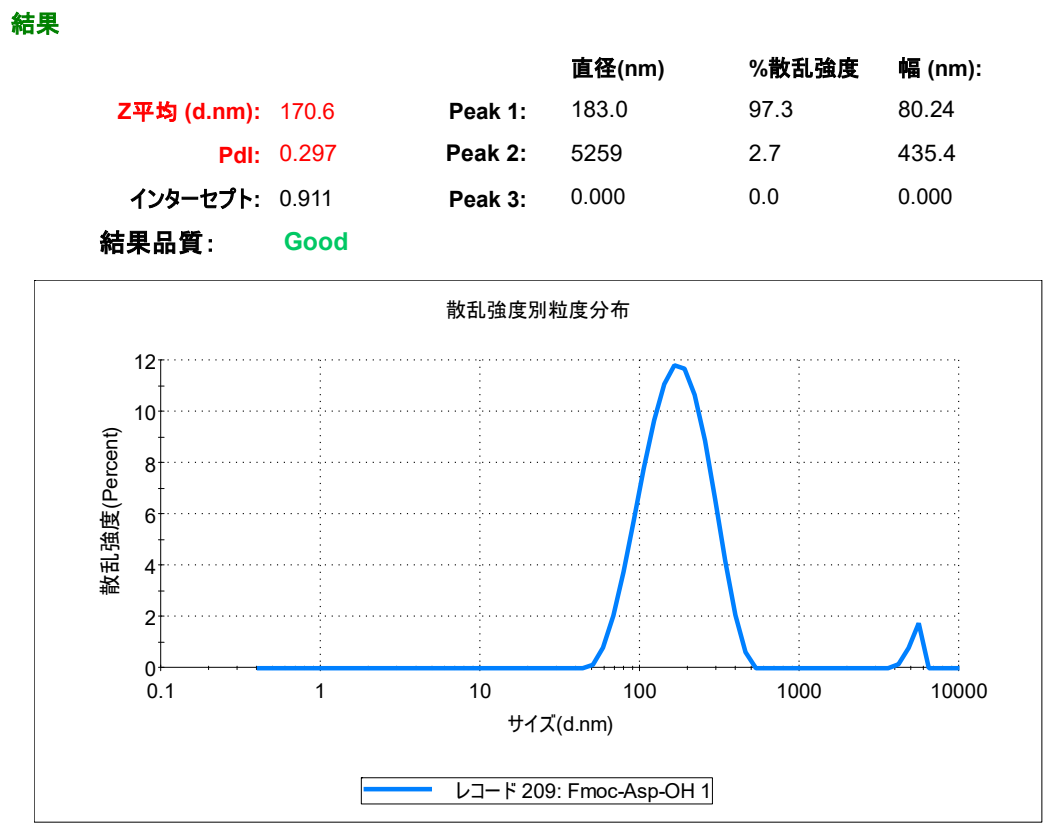

(b) Number-weighted distribution

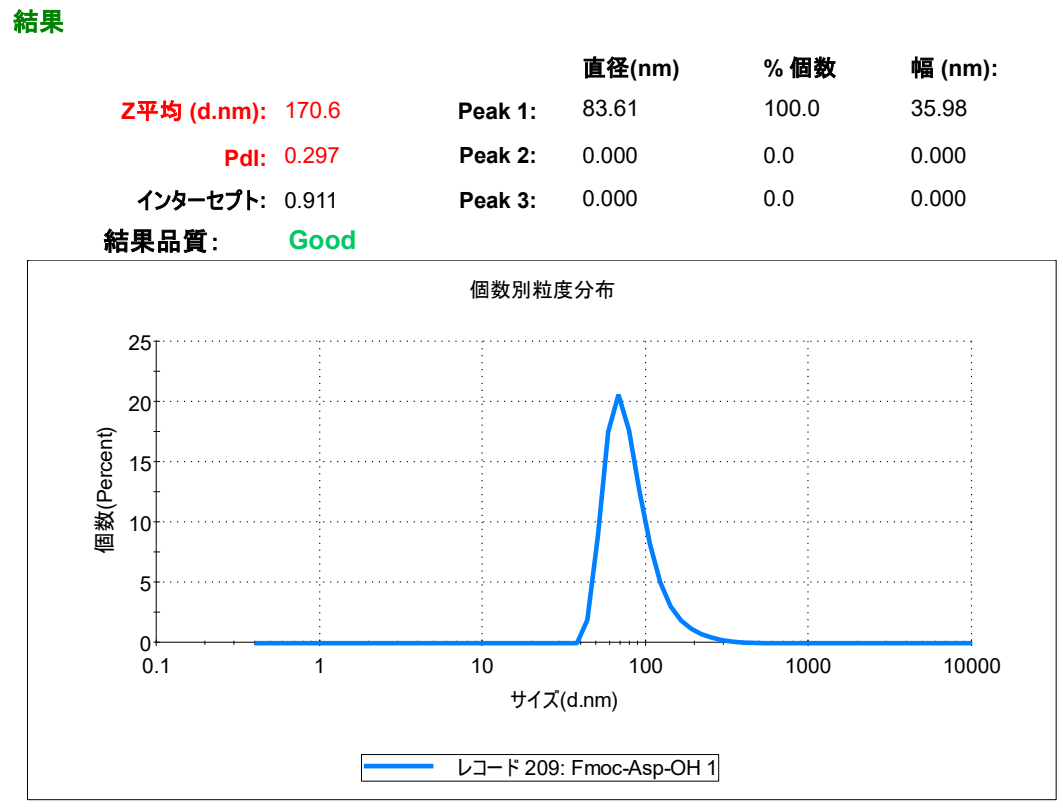

B: Three-Component Nanoassemblies

(a) Intensity-weighted distribution

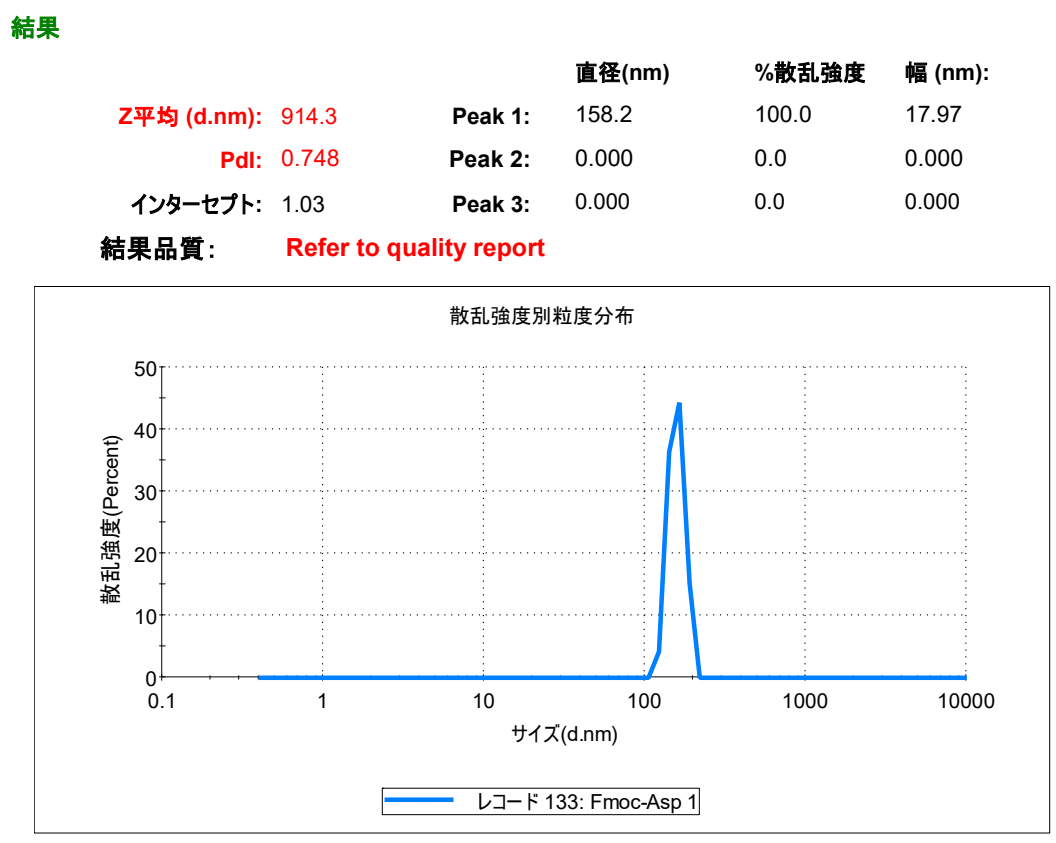

(b) Number-weighted distribution

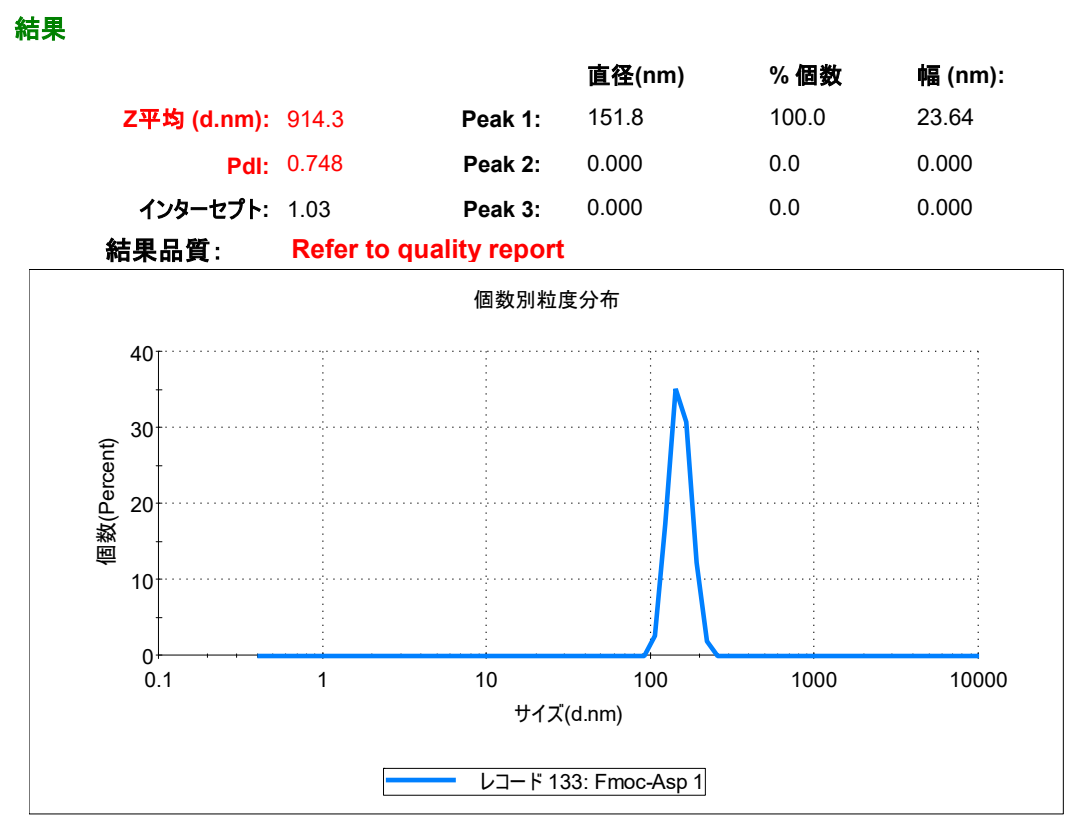

Figure S6. DLS Analysis of Fmoc-Asp(OtBu)-OH Nanoassemblies.

(Japanese labels appearing in the DLS output figures are defined in Table S1.)

A: Two-Component Nanoassemblies (Fmoc-Asp(OtBu)-OH and DIEA).

(a) The intensity-weighted distribution; (b) The corresponding number-weighted distribution

B: Three-Component Nanoassemblies (Fmoc-Asp(OtBu)-OH, DIEA and TBTU).

(a) The intensity-weighted distribution; (b) The number-weighted distribution.

A: Two-Component Nanoassemblies

(a) Intensity-weighted distribution

結果

			直径(nm)	%散乱強度	幅 (nm):
Z平均 (d.nm):	275.9	Peak 1:	235.7	87.3	61.10
PdI:	0.343	Peak 2:	66.20	12.7	11.08
インターセプト:	0.911	Peak 3:	0.000	0.0	0.000
結果品質：	Refer to quality report				

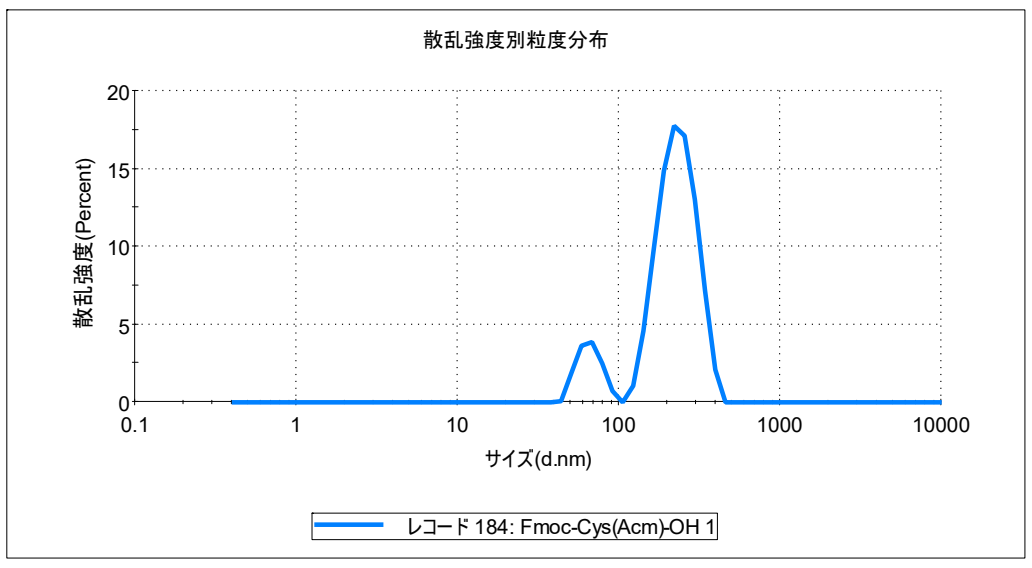

(b) Number-weighted distribution

結果

			直径(nm)	% 個数	幅 (nm):
Z平均 (d.nm):	275.9	Peak 1:	191.9	2.8	60.09
PdI:	0.343	Peak 2:	57.84	97.2	10.49
インターセプト:	0.911	Peak 3:	0.000	0.0	0.000
結果品質：	Refer to quality report				

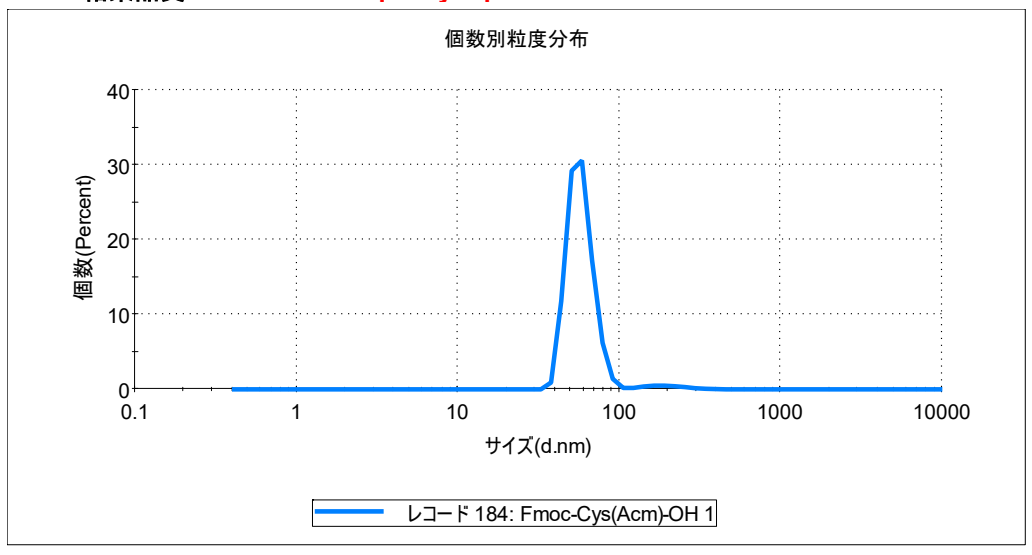

B: Three-Component Nanoassemblies

(a) Intensity-weighted distribution

結果

			直径(nm)	%散乱強度	幅 (nm):
Z平均 (d.nm):	507.7	Peak 1:	358.9	72.9	100.1
PdI:	0.502	Peak 2:	91.62	27.1	25.93
インターセプト:	0.872	Peak 3:	0.000	0.0	0.000
結果品質：	Refer to quality report				

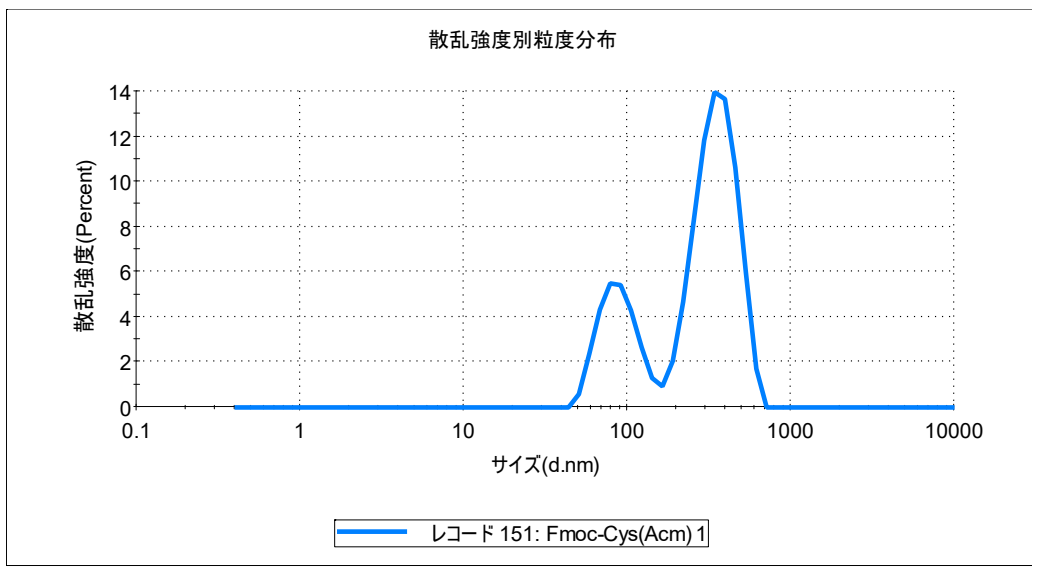

(b) Number-weighted distribution

結果

			直径(nm)	% 個数	幅 (nm):
Z平均 (d.nm):	507.7	Peak 1:	299.2	1.3	99.72
PdI:	0.502	Peak 2:	66.74	98.7	15.94
インターセプト:	0.872	Peak 3:	0.000	0.0	0.000
結果品質：	Refer to quality report				

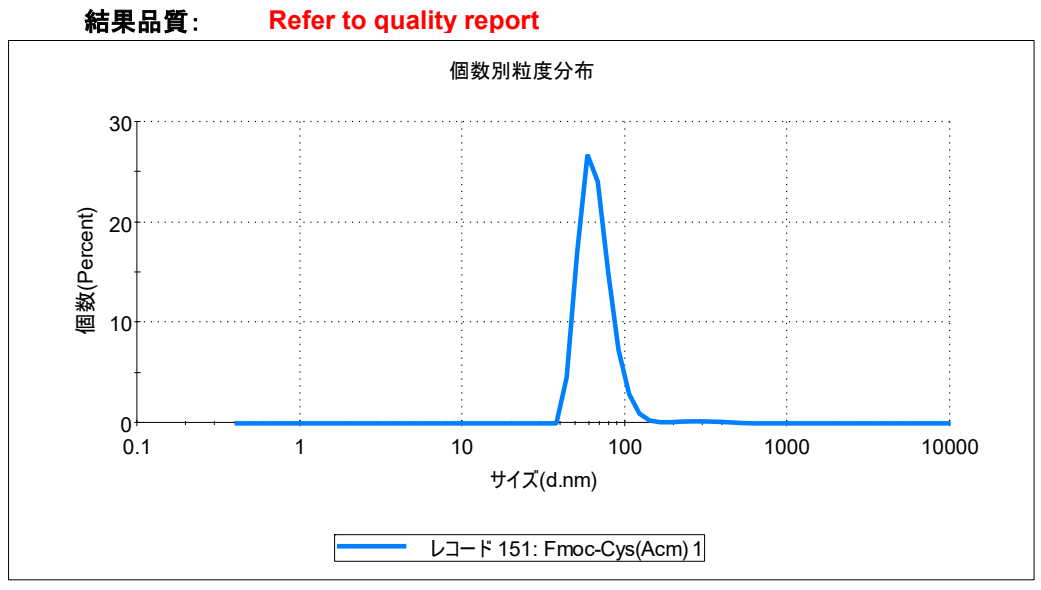

Figure S7. DLS Analysis of Fmoc-Cys(Acm)-OH Nanoassemblies.

(Japanese labels appearing in the DLS output figures are defined in Table S1.)

A: Two-Component Nanoassemblies (Fmoc-Cys(Acm)-OH and DIEA).

(a) The intensity-weighted distribution; (b) The corresponding number-weighted distribution

B: Three-Component Nanoassemblies (Fmoc-Cys(Acm)-OH, DIEA and TBTU).

(a) The intensity-weighted distribution; (b) The number-weighted distribution revealed a predominant particle population together with minor smaller species.

A: Two-Component Nanoassemblies

(a) Intensity-weighted distribution

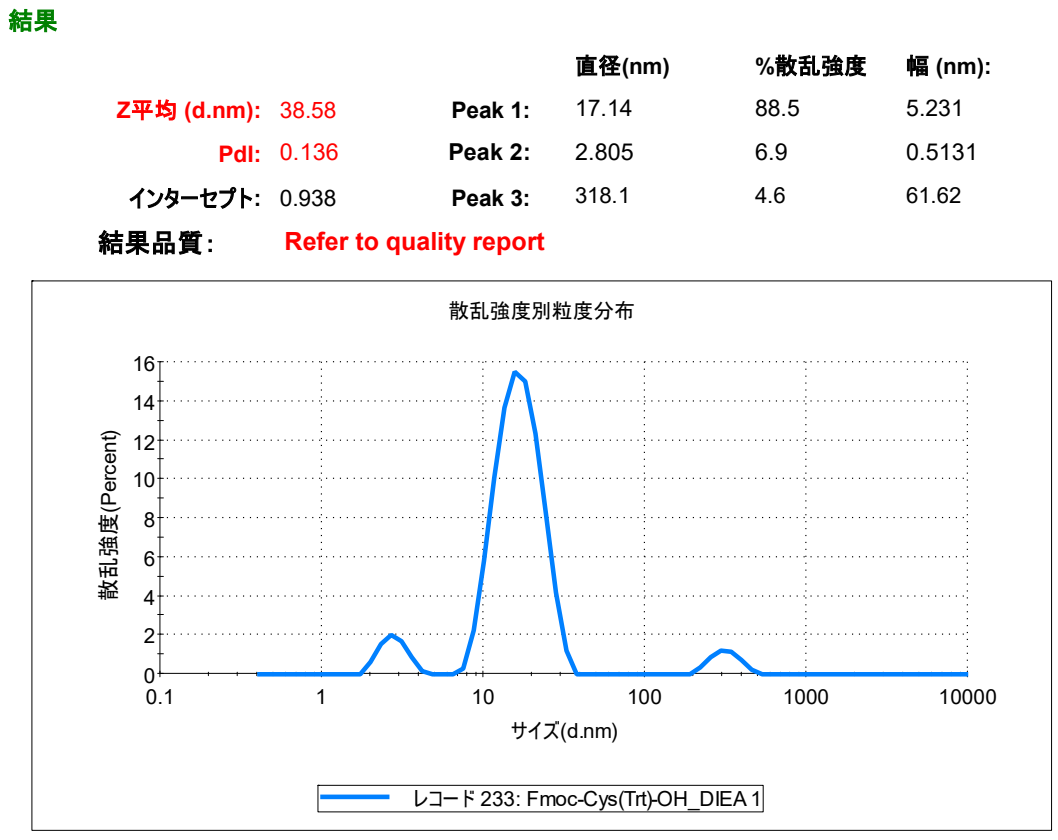

(b) Number-weighted distribution

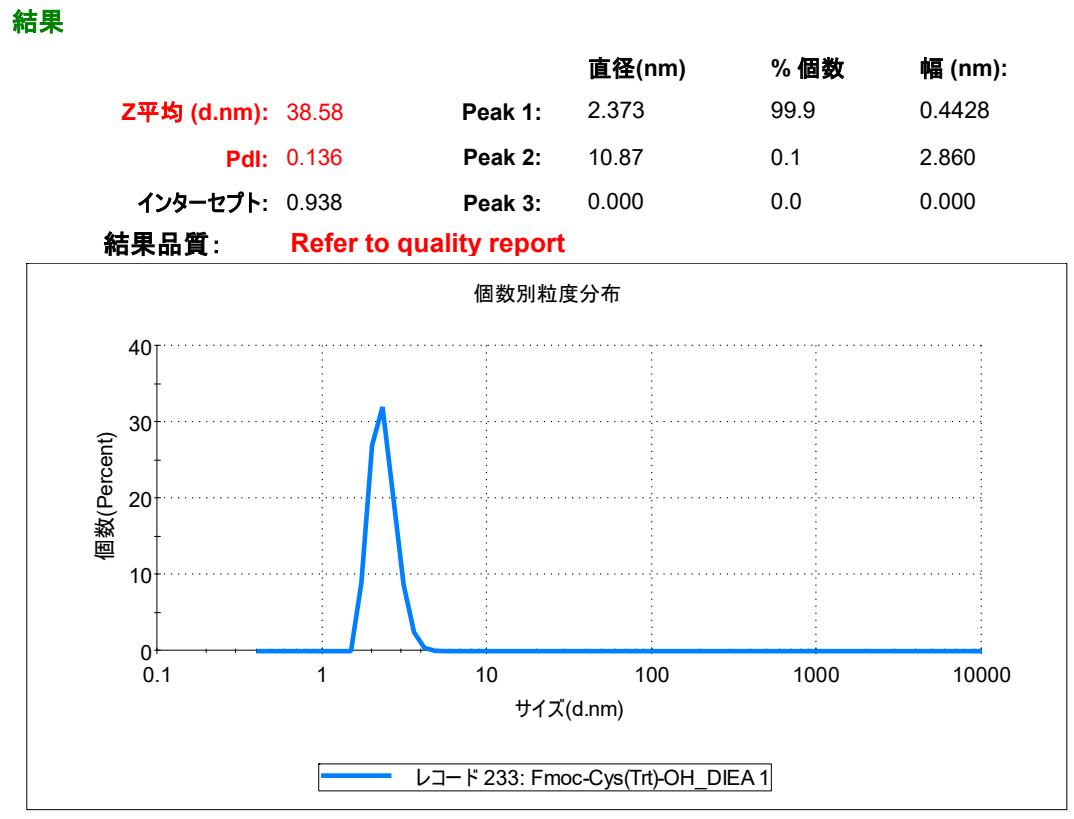

B: Three-Component Nanoassemblies

(a) Intensity-weighted distribution

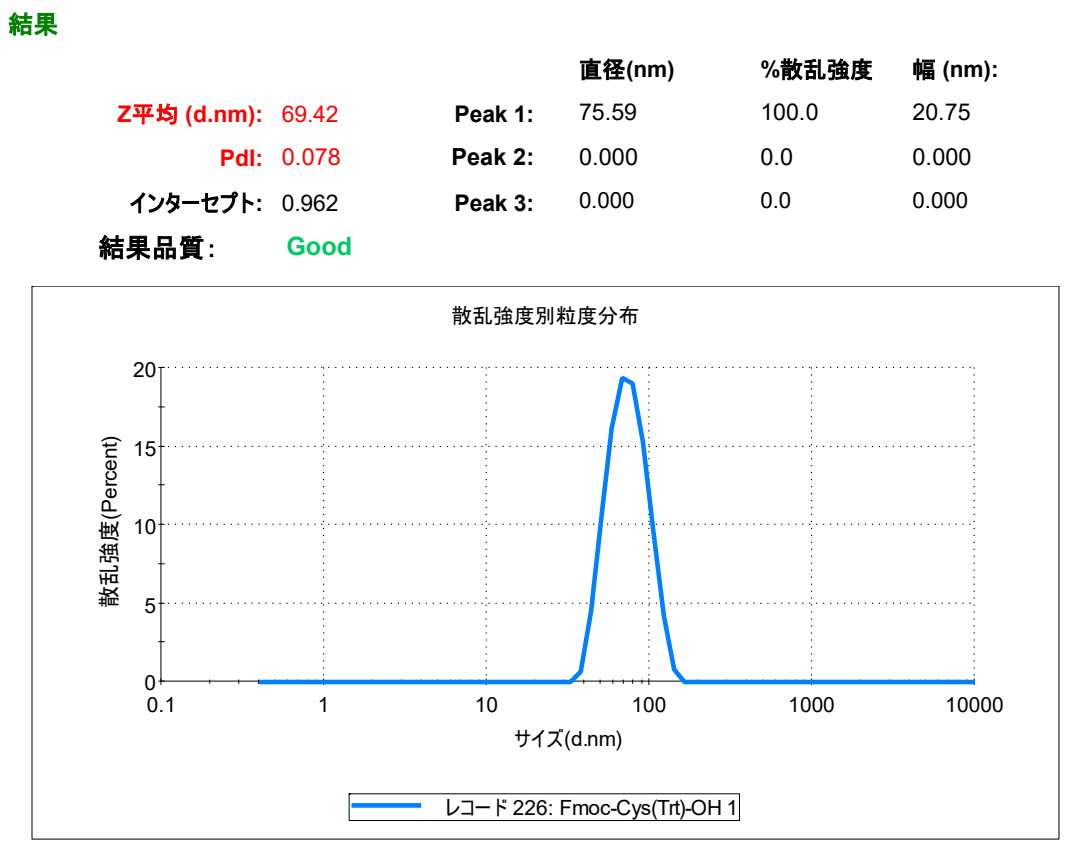

(b) Number-weighted distribution

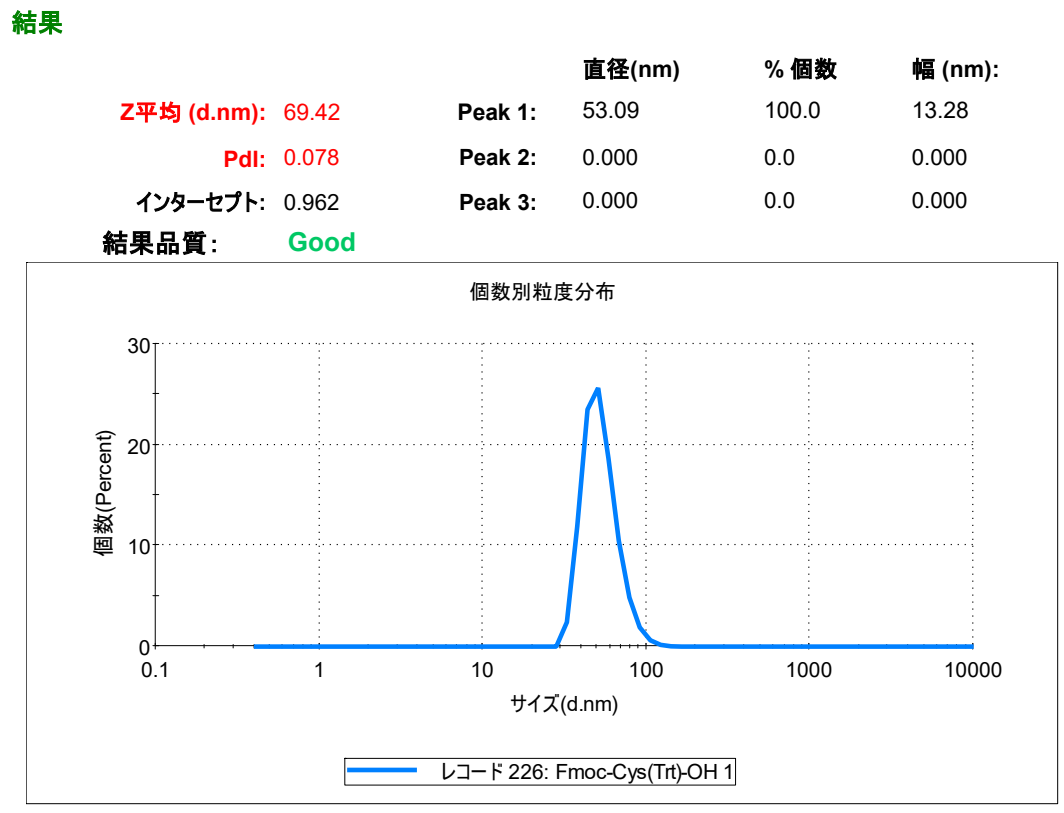

Figure S8. DLS Analysis of Fmoc-Cys(Trt)-OH Nanoassemblies.

(Japanese labels appearing in the DLS output figures are defined in Table S1.)

A: Two-Component Nanoassemblies (Fmoc-Cys(Trt)-OH and DIEA).

(a) The intensity-weighted distribution; (b) The corresponding number-weighted distribution

B: Three-Component Nanoassemblies (Fmoc-Cys(Trt)-OH, DIEA and TBTU).

(a) The intensity-weighted distribution; (b) The number-weighted distribution

A: Two-Component Nanoassemblies

(a) Intensity-weighted distribution

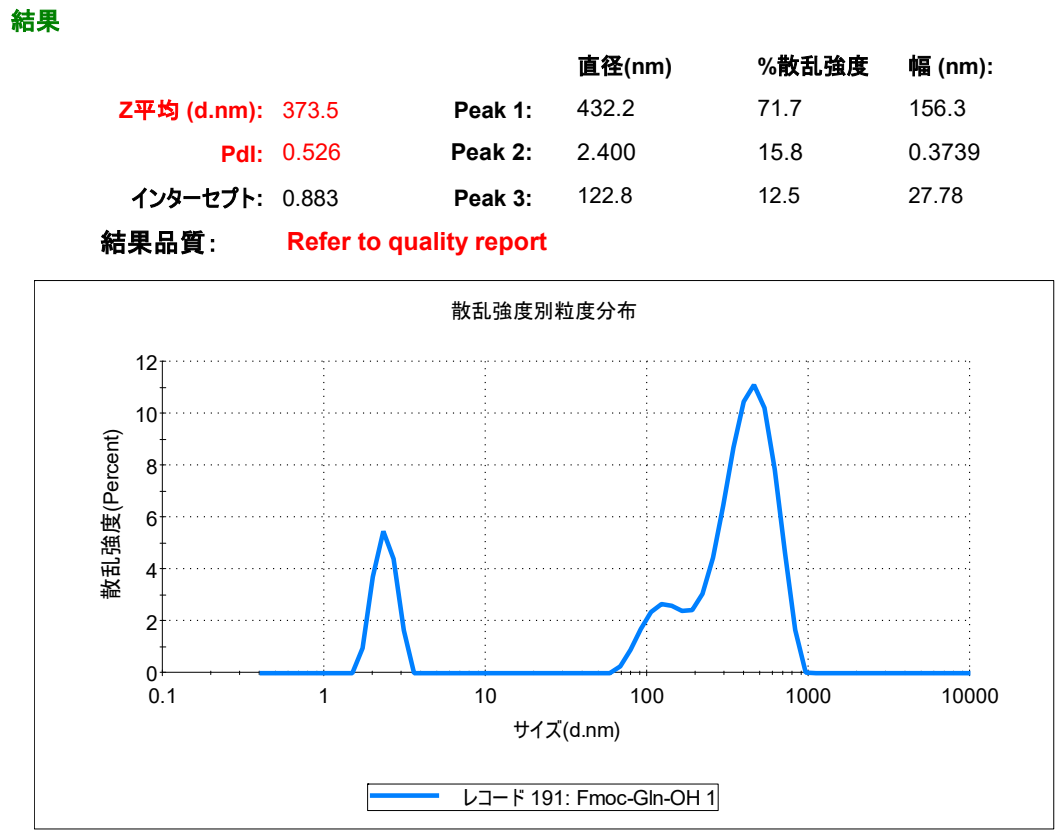

(b) Number-weighted distribution

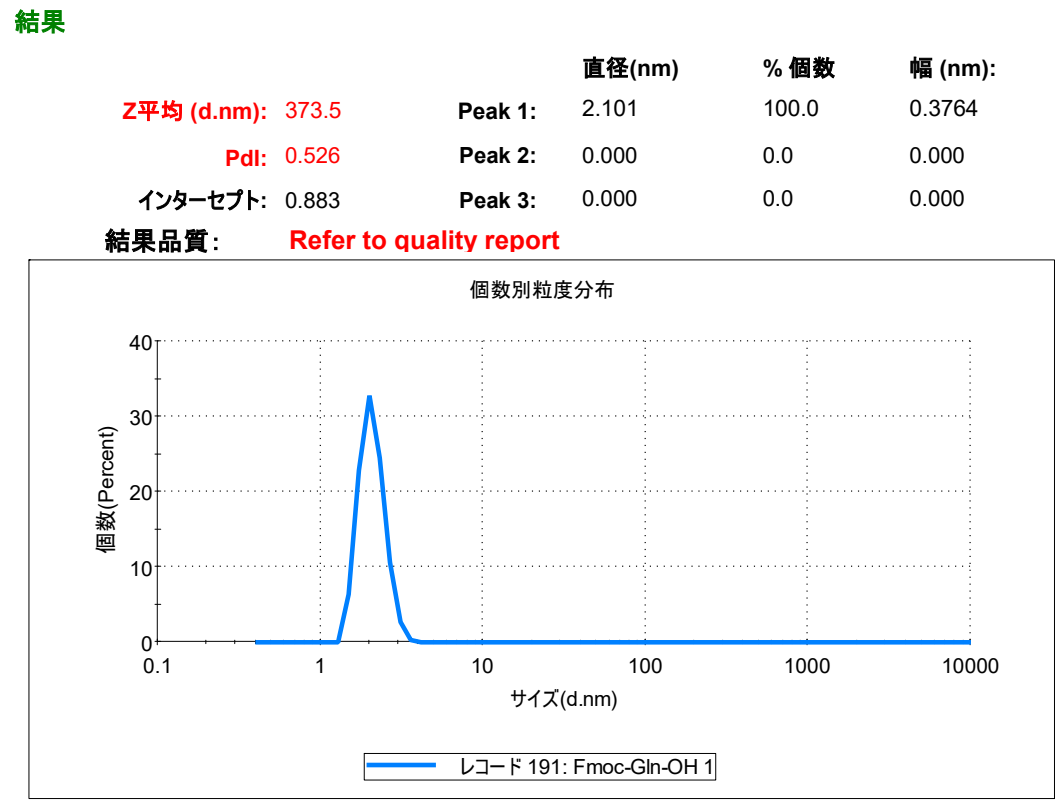

B: Three-Component Nanoassemblies

(a) Intensity-weighted distribution

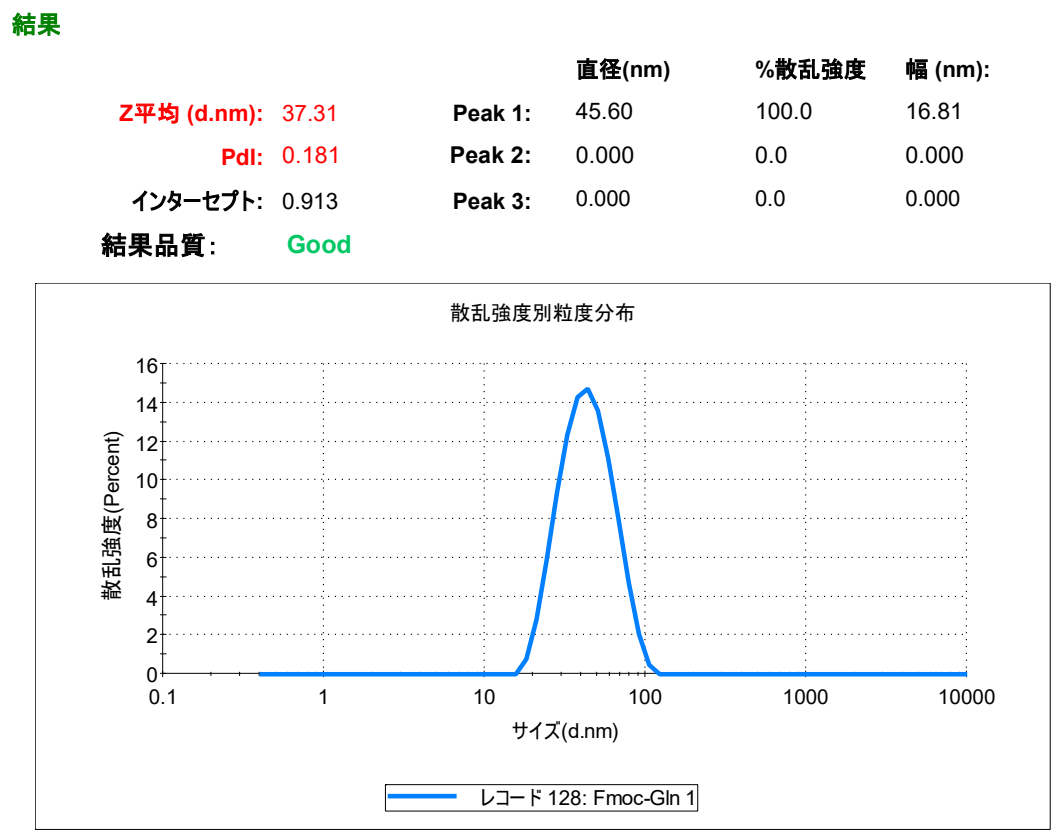

(b) Number-weighted distribution

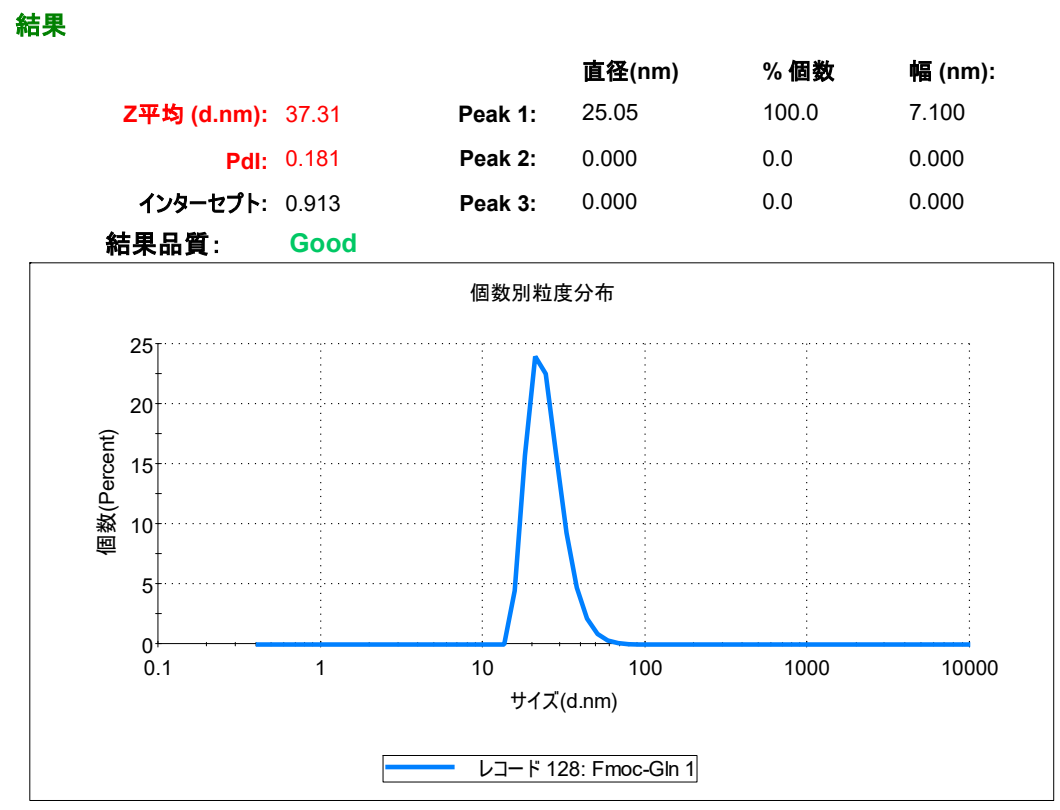

Figure S9. DLS Analysis of Fmoc-Gln(Trt)-OH Nanoassemblies.

(Japanese labels appearing in the DLS output figures are defined in Table S1.)

A: Two-Component Nanoassemblies (Fmoc-Gln(Trt)-OH and DIEA).

(a) The intensity-weighted distribution; (b) The corresponding number-weighted distribution

B: Three-Component Nanoassemblies (Fmoc-Gln(Trt)-OH, DIEA and TBTU).

(a) The intensity-weighted distribution; (b) The number-weighted distribution.

A: Two-Component Nanoassemblies

(a) Intensity-weighted distribution

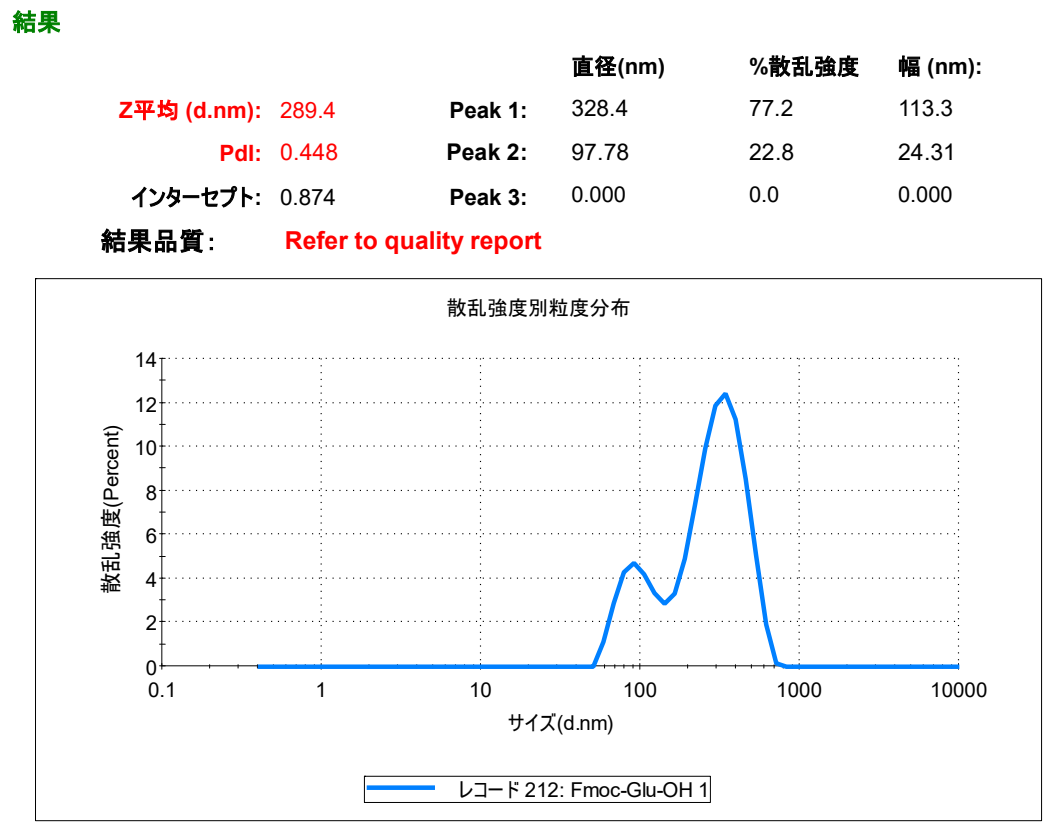

(b) Number-weighted distribution

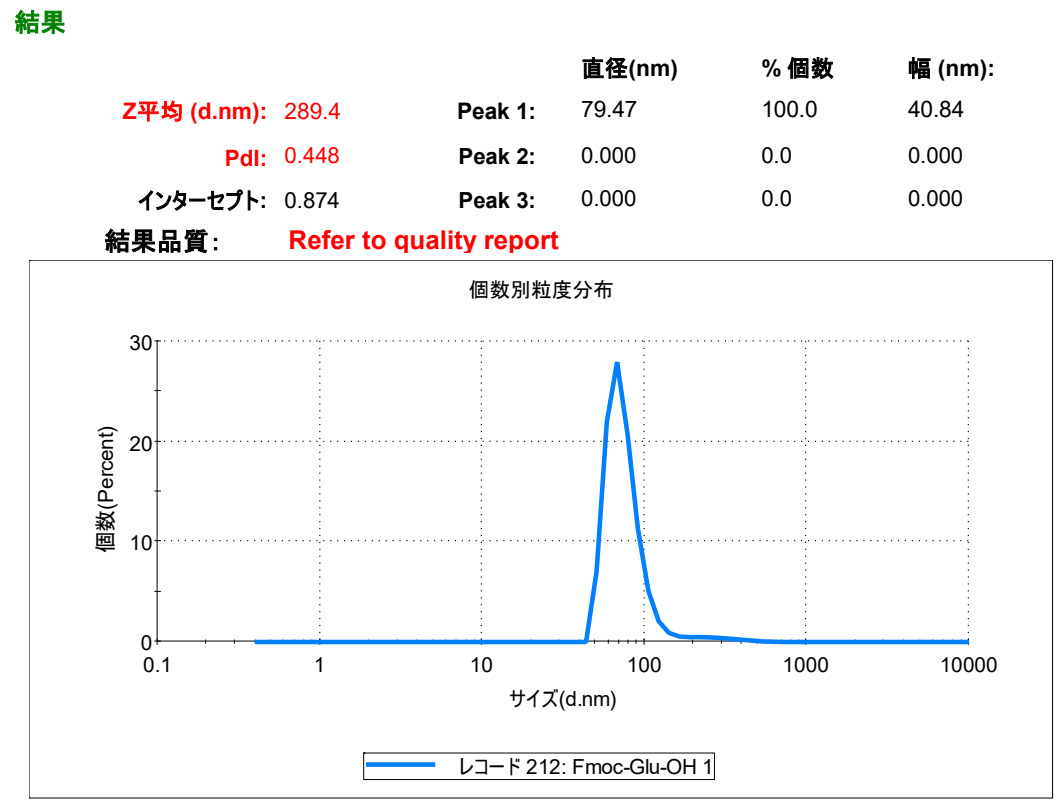

B: Three-Component Nanoassemblies

(a) Intensity-weighted distribution

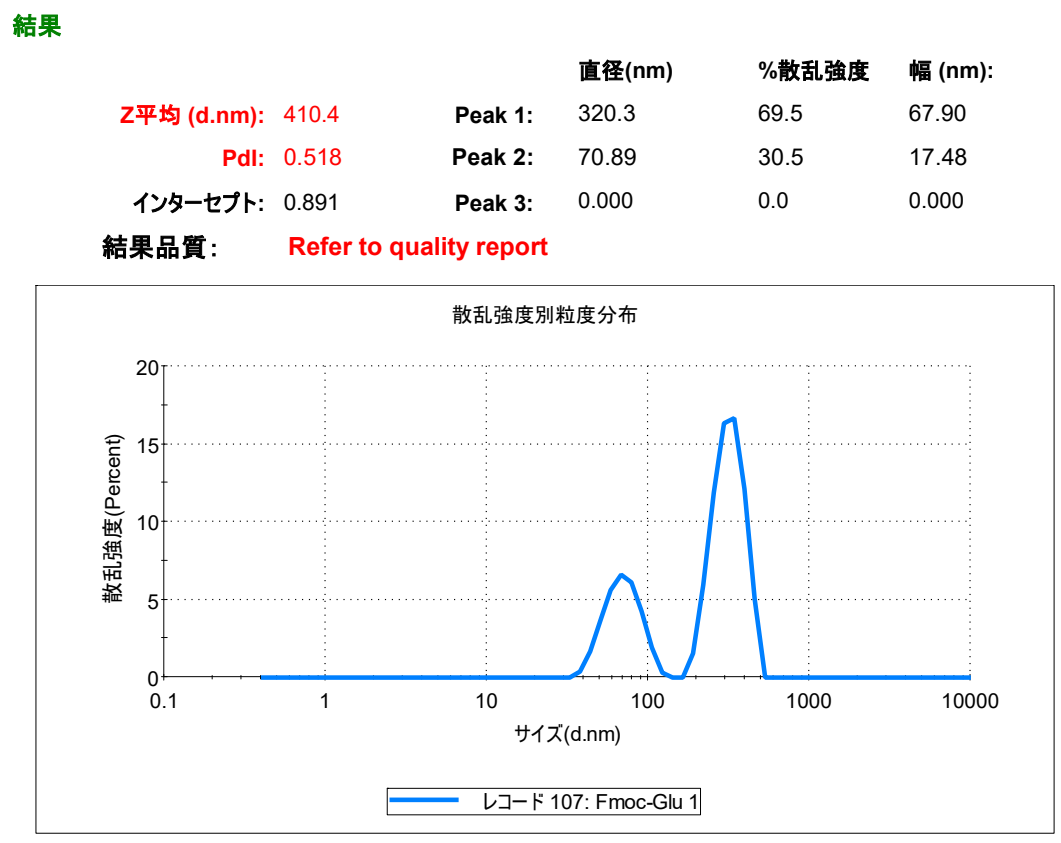

(b) Number-weighted distribution

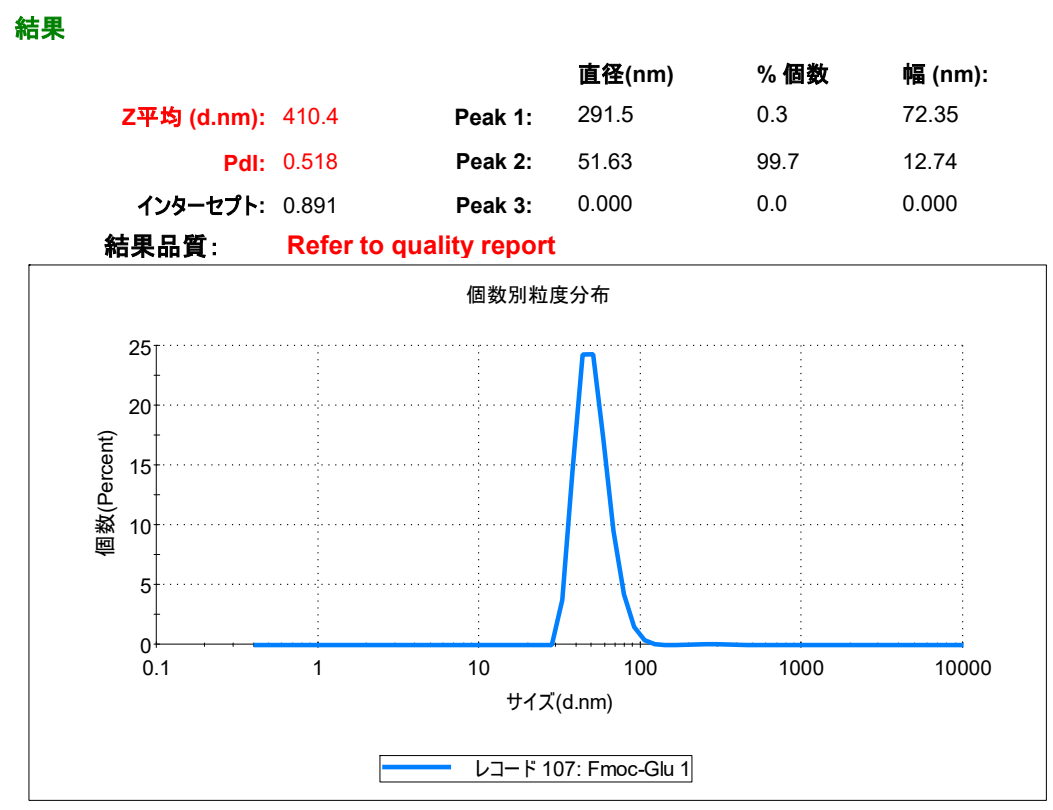

Figure S10. DLS Analysis of Fmoc-Glu(OtBu)-OH Nanoassemblies.

(Japanese labels appearing in the DLS output figures are defined in Table S1.)

A: Two-Component Nanoassemblies (Fmoc-Glu(OtBu)-OH and DIEA).

(a) The intensity-weighted distribution; (b) The corresponding number-weighted distribution.

B: Three-Component Nanoassemblies (Fmoc-Glu(OtBu)-OH, DIEA and TBTU).

(a) The intensity-weighted distribution; (b) The number-weighted distribution.

A: Two-Component Nanoassemblies

(a) Intensity-weighted distribution

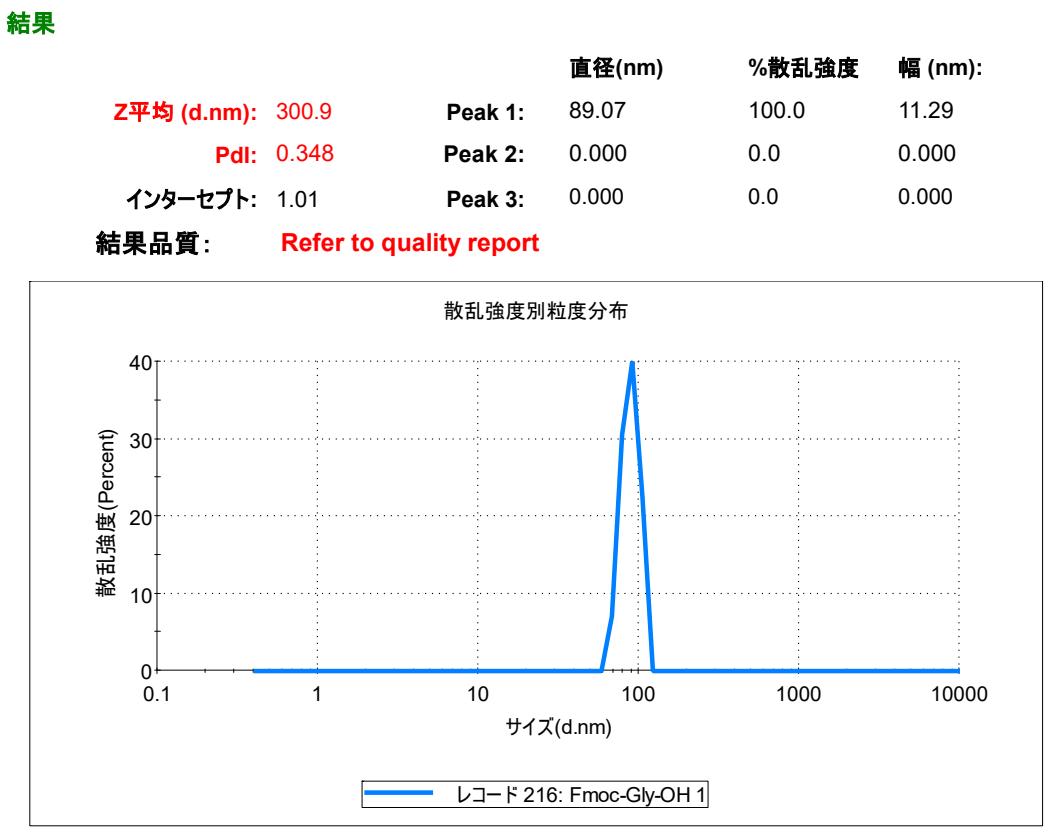

(b) Number-weighted distribution

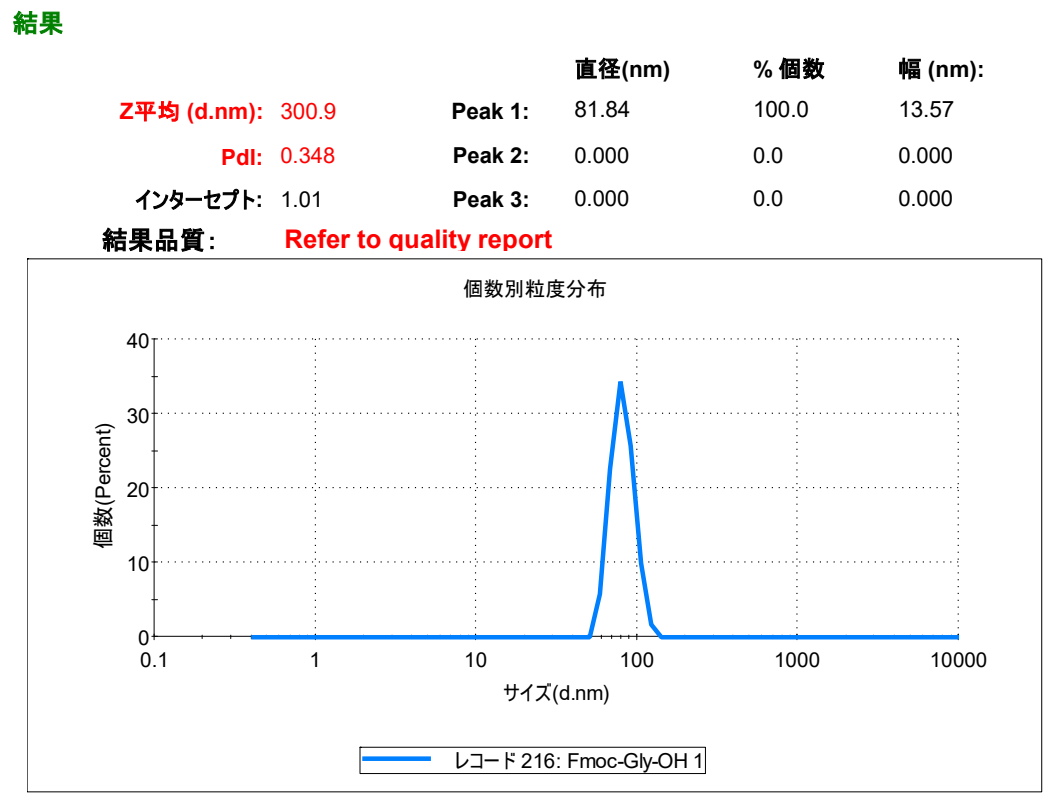

B: Three-Component Nanoassemblies

(a) Intensity-weighted distribution

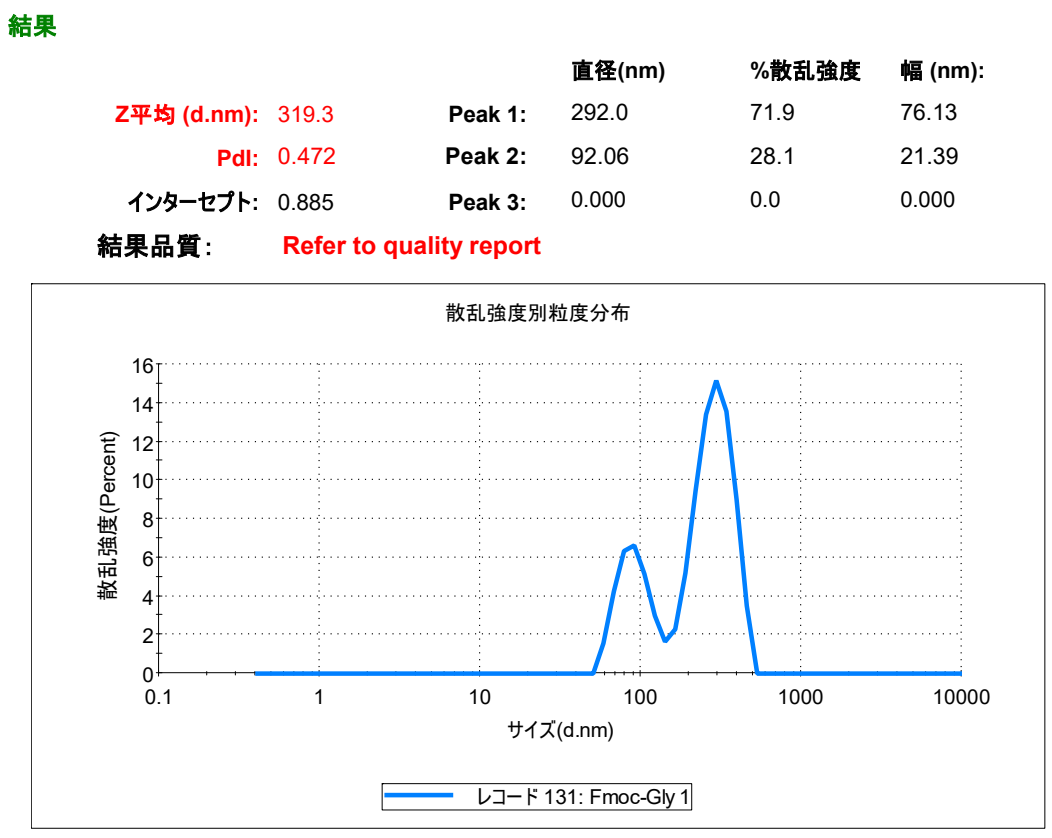

(b) Number-weighted distribution

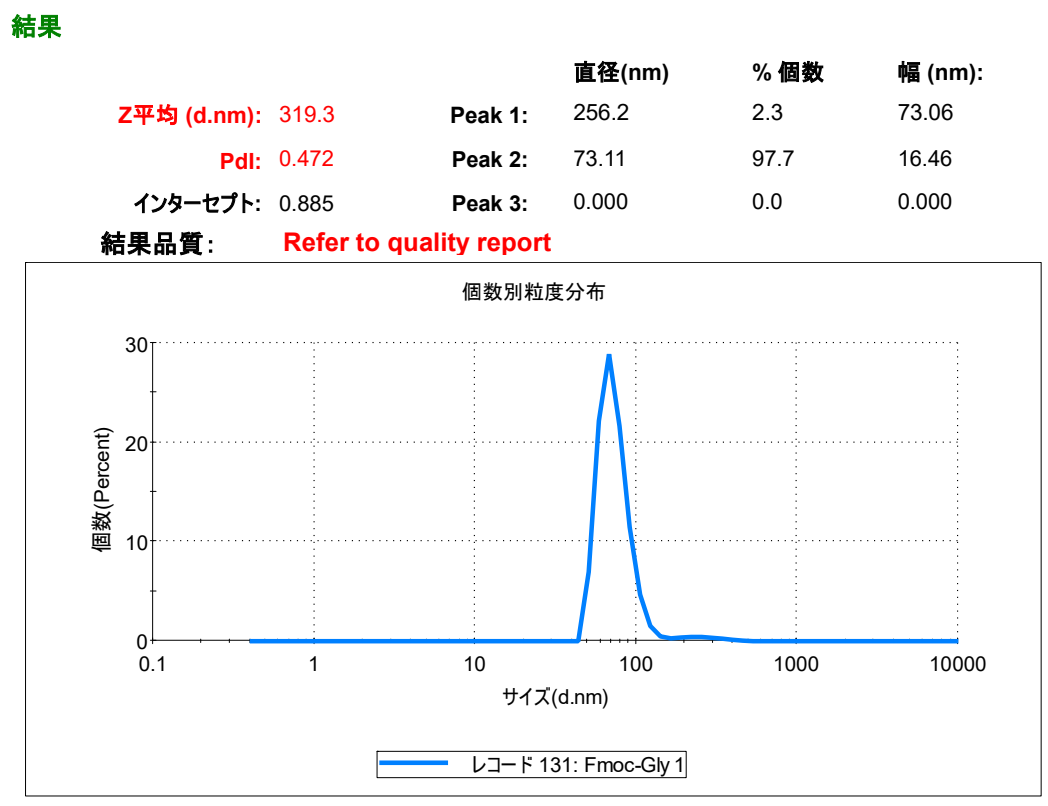

Figure S11. DLS Analysis of Fmoc-Gly-OH Nanoassemblies.

(Japanese labels appearing in the DLS output figures are defined in Table S1.)

A: Two-Component Nanoassemblies (Fmoc-Gly-OH and DIEA).

(a) The intensity-weighted distribution; (b) The corresponding number-weighted distribution.

B: Three-Component Nanoassemblies (Fmoc-Gly-OH, DIEA and TBTU).

(a) The intensity-weighted distribution; (b) The number-weighted distribution.

A: Two-Component Nanoassemblies

(a) Intensity-weighted distribution

結果

			直径(nm)	%散乱強度	幅 (nm):
Z平均 (d.nm):	258.1	Peak 1:	302.9	72.4	128.1
PdI:	0.339	Peak 2:	95.55	18.6	19.48
インターセプト:	0.850	Peak 3:	1.929	9.0	0.2578
結果品質：	Refer to quality report				

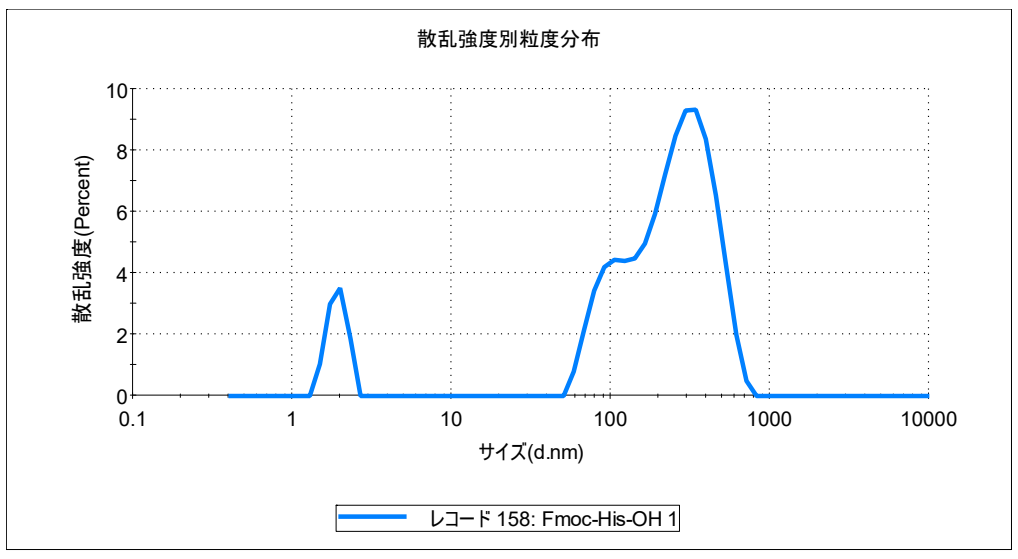

(b) Number-weighted distribution

結果

			直径(nm)	% 個数	幅 (nm):
Z平均 (d.nm):	258.1	Peak 1:	1.744	100.0	0.2894
PdI:	0.339	Peak 2:	0.000	0.0	0.000
インターセプト:	0.850	Peak 3:	0.000	0.0	0.000
結果品質：	Refer to quality report				

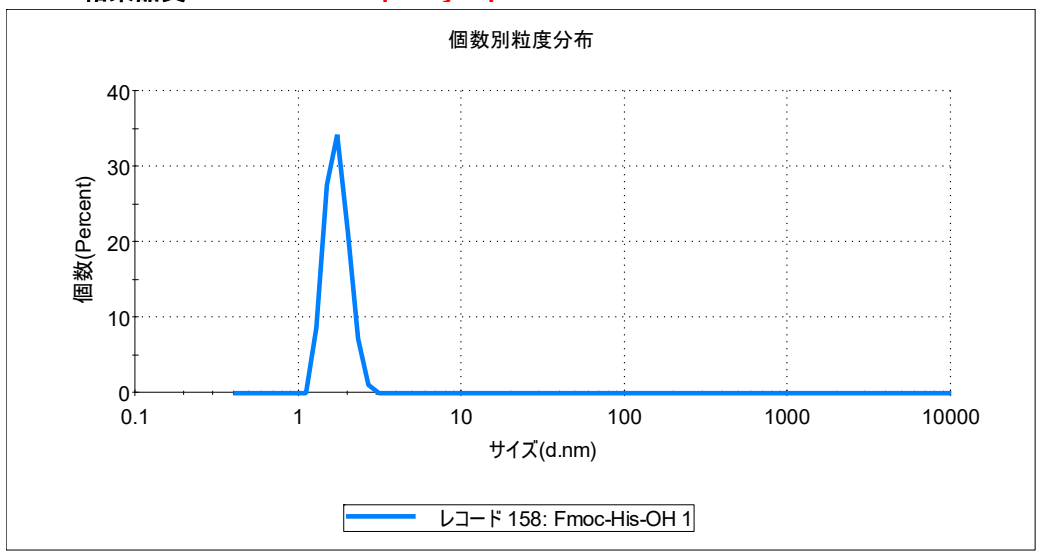

B: Three-Component Nanoassemblies

(a) Intensity-weighted distribution

結果

			直径(nm)	%散乱強度	幅 (nm):
Z平均 (d.nm):	142.1	Peak 1:	54.84	62.2	19.34
PdI:	0.228	Peak 2:	200.3	37.8	63.53
インターセプト:	0.932	Peak 3:	0.000	0.0	0.000
結果品質：	Refer to quality report				

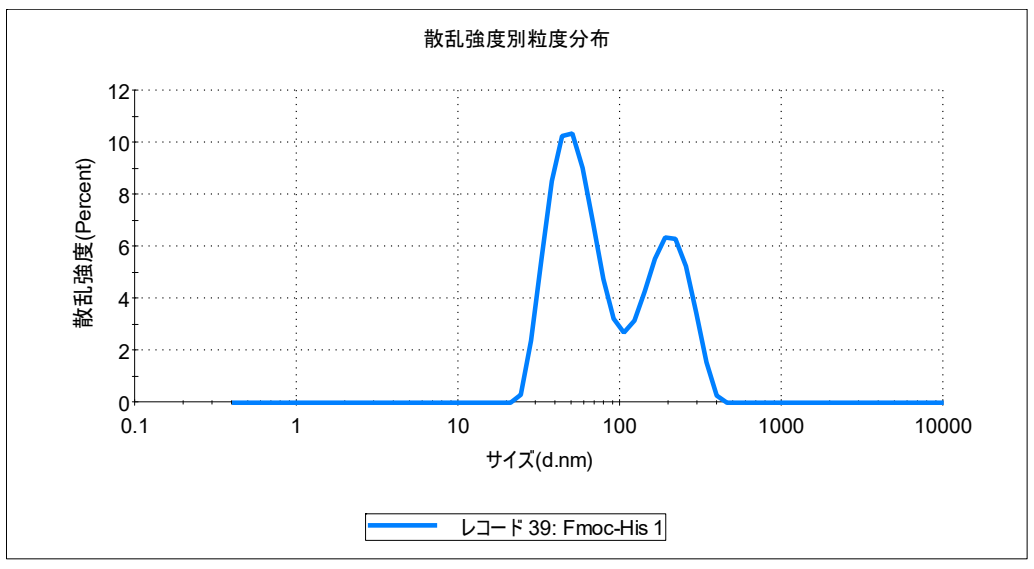

(b) Number-weighted distribution

結果

			直径(nm)	% 個数	幅 (nm):
Z平均 (d.nm):	142.1	Peak 1:	34.24	100.0	9.044
PdI:	0.228	Peak 2:	0.000	0.0	0.000
インターセプト:	0.932	Peak 3:	0.000	0.0	0.000
結果品質：	Refer to quality report				

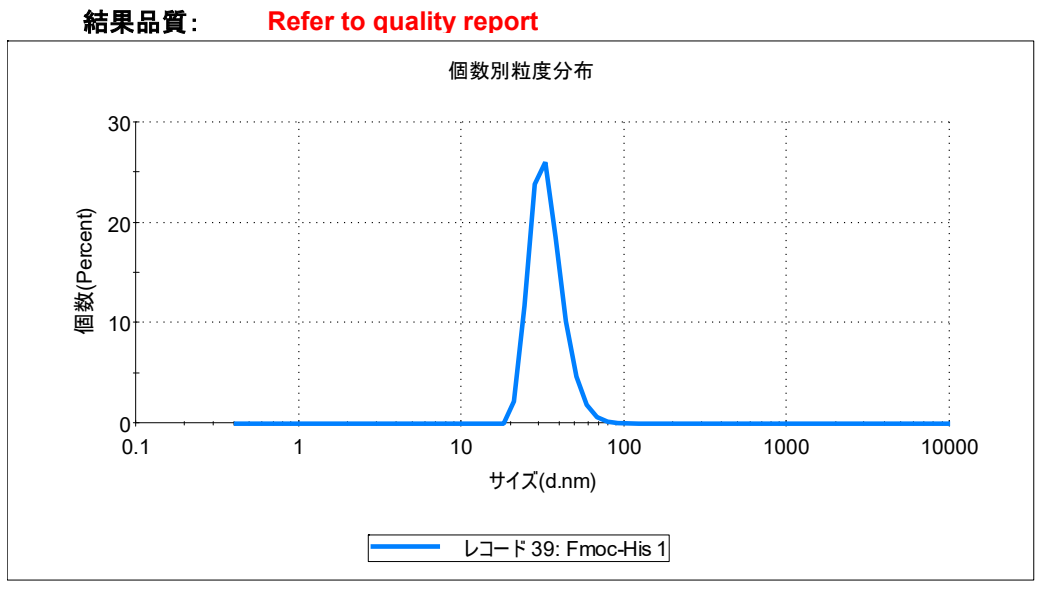

Figure S12. DLS Analysis of Fmoc-His(Boc)-OH Nanoassemblies.

(Japanese labels appearing in the DLS output figures are defined in Table S1.)

A: Two-Component Nanoassemblies (Fmoc-His(Boc)-OH and DIEA).

(a) The intensity-weighted distribution; (b) The corresponding number-weighted distribution.

B: Three-Component Nanoassemblies (Fmoc-His(Boc)-OH, DIEA and TBTU).

(a) The intensity-weighted distribution; (b) The number-weighted distribution.

A: Two-Component Nanoassemblies

(a) Intensity-weighted distribution

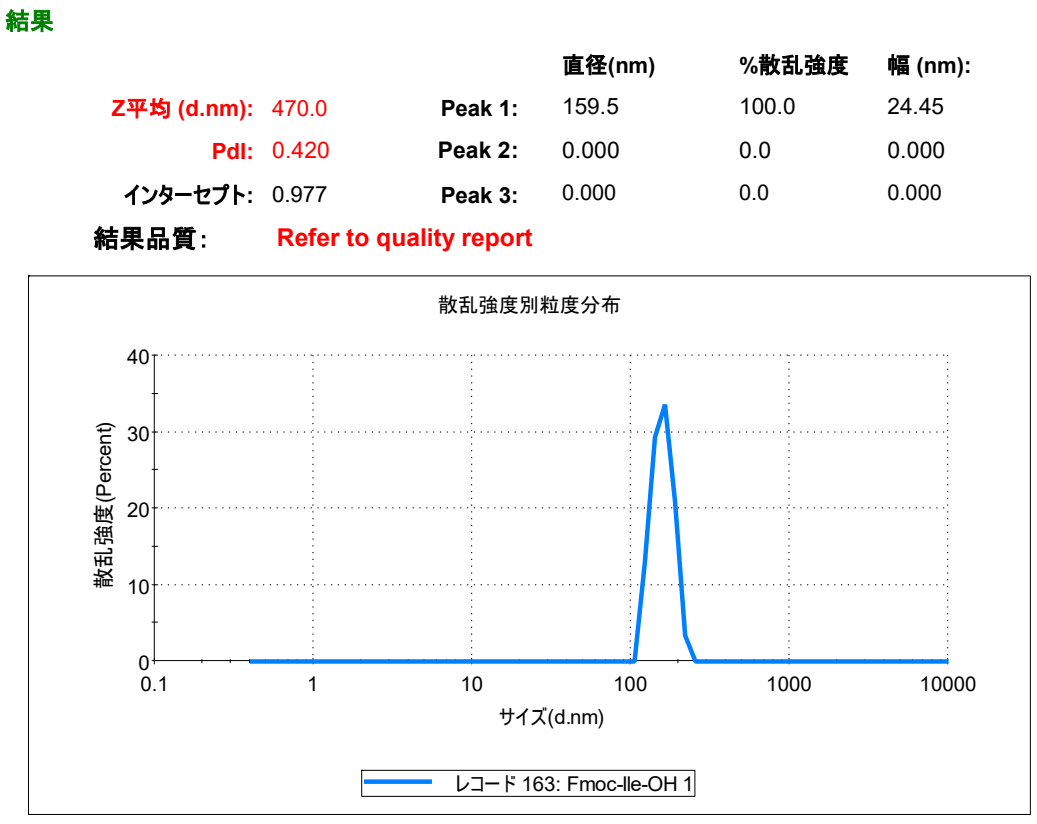

(b) Number-weighted distribution

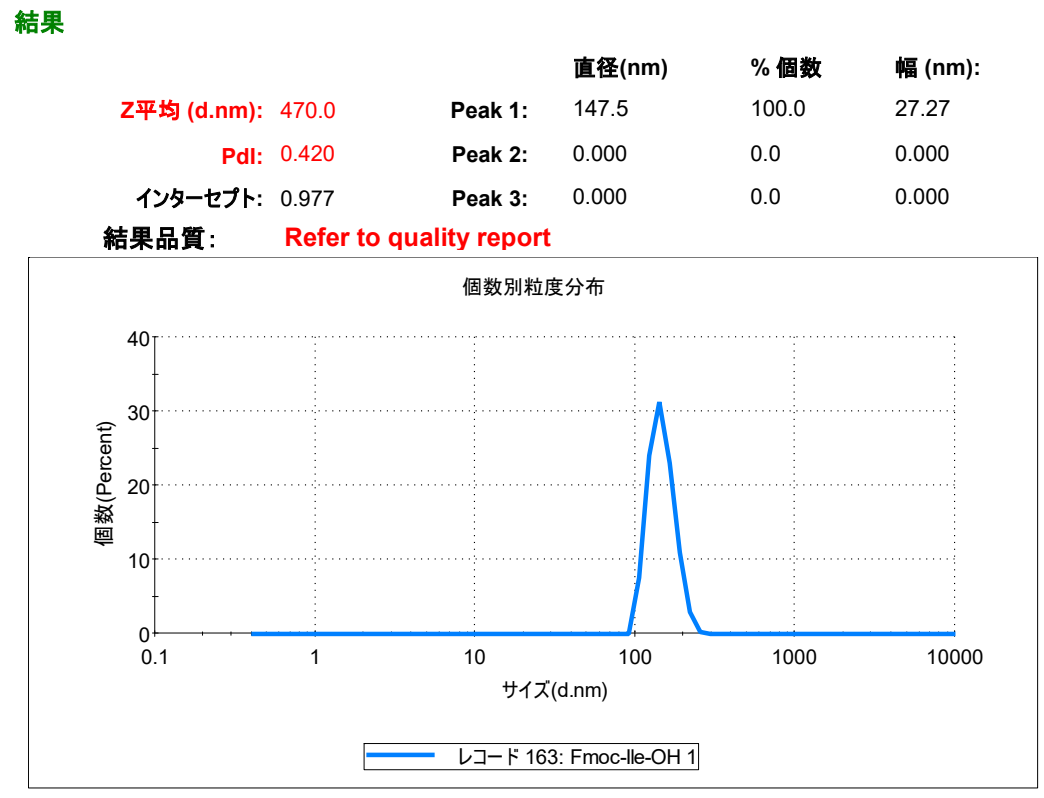

B: Three-Component Nanoassemblies

(a) Intensity-weighted distribution

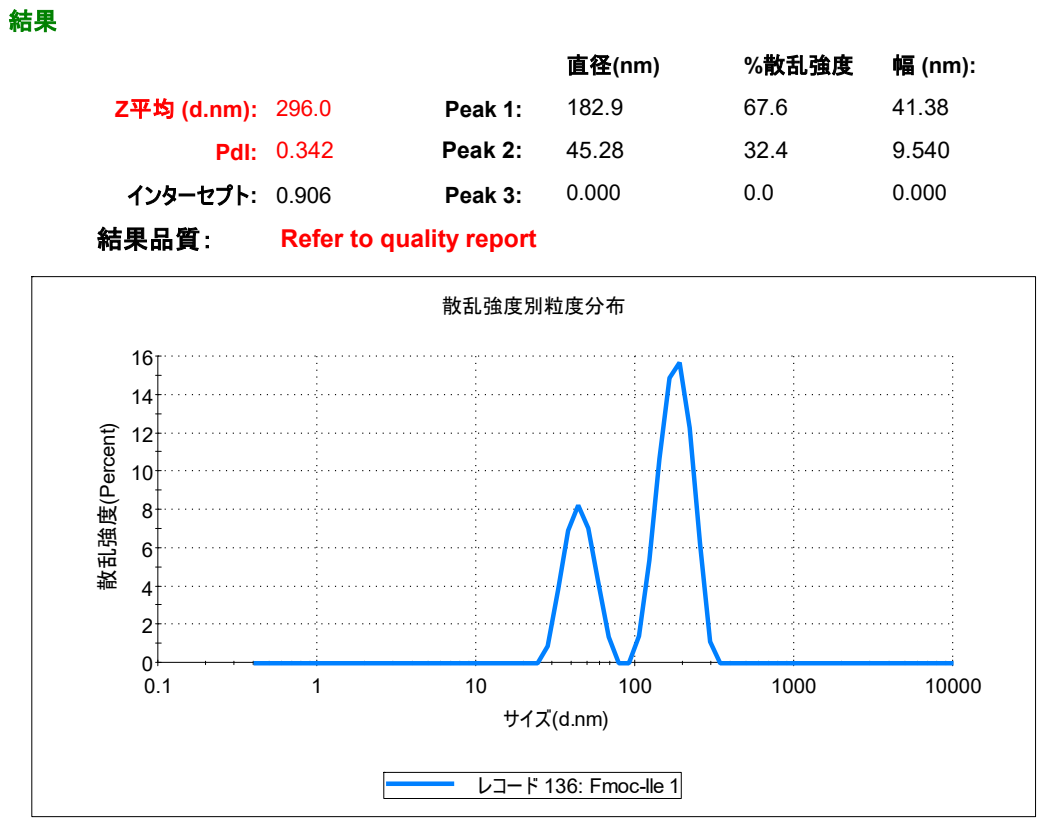

(b) Number-weighted distribution

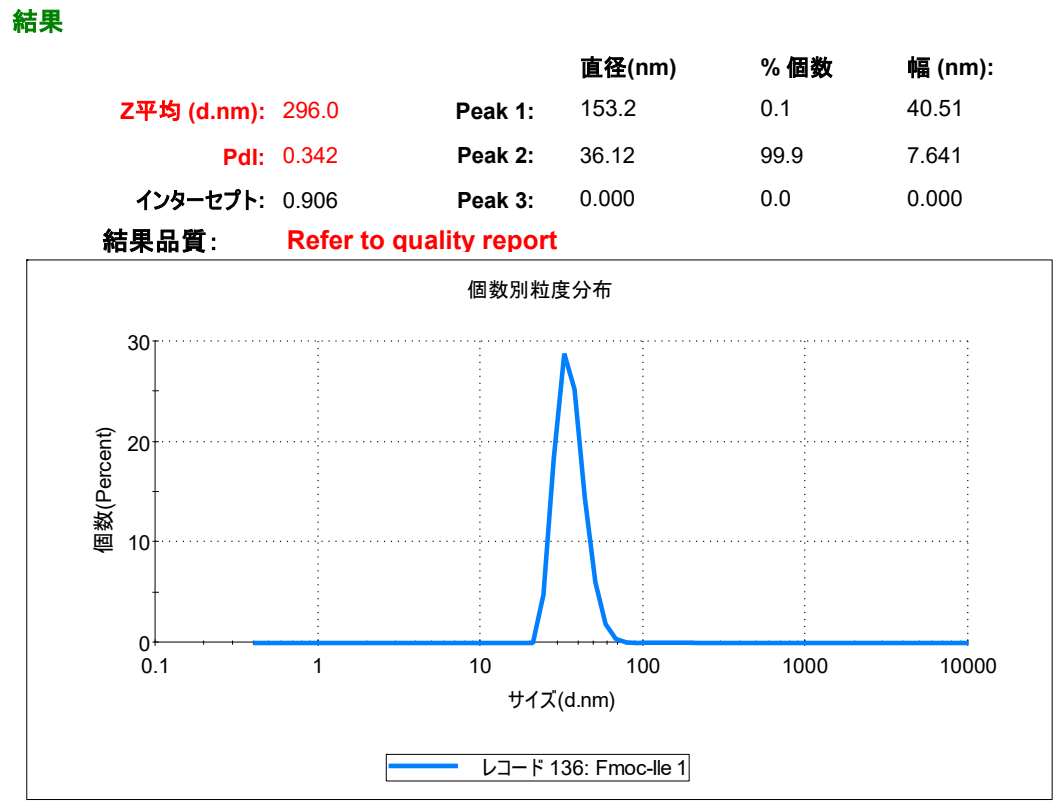

Figure S13. DLS Analysis of Fmoc-Ile-OH Nanoassemblies.

(Japanese labels appearing in the DLS output figures are defined in Table S1.)

A: Two-Component Nanoassemblies (Fmoc-Ile-OH and DIEA).

(a) The intensity-weighted distribution; (b) The corresponding number-weighted distribution.

B: Three-Component Nanoassemblies (Fmoc-Ile-OH, DIEA and TBTU).

(a) The intensity-weighted distribution; (b) The number-weighted distribution.

A: Two-Component Nanoassemblies

(a) Intensity-weighted distribution

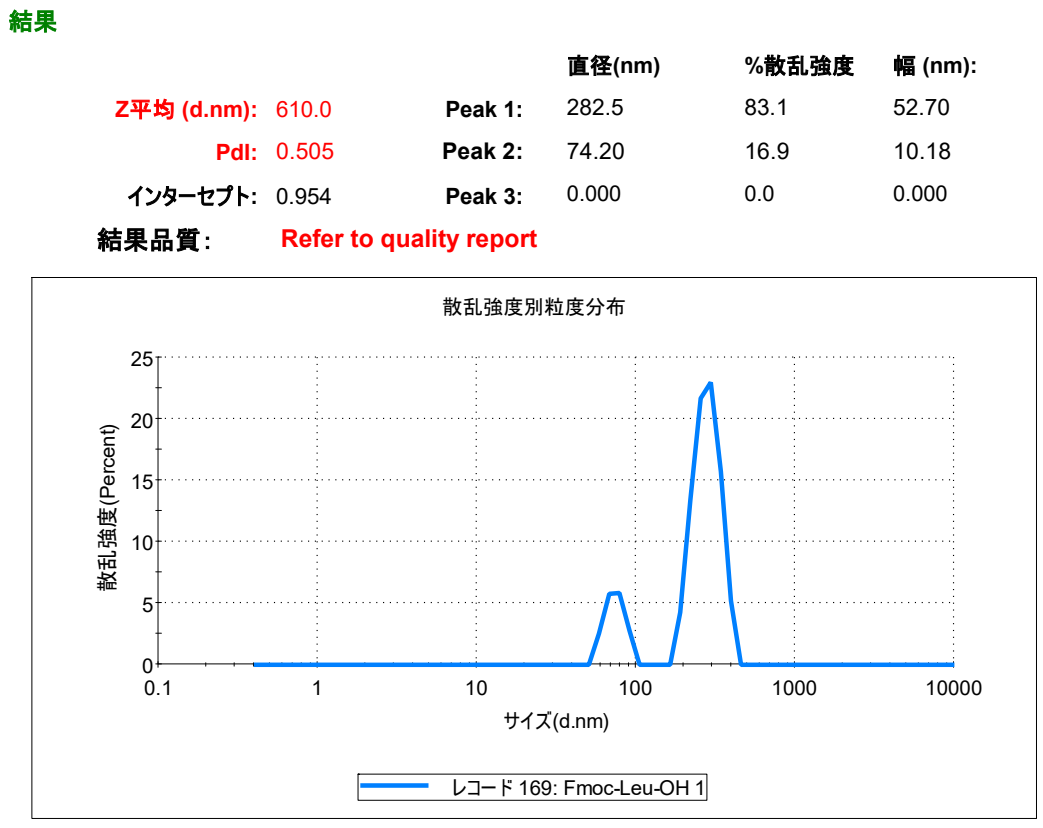

(b) Number-weighted distribution

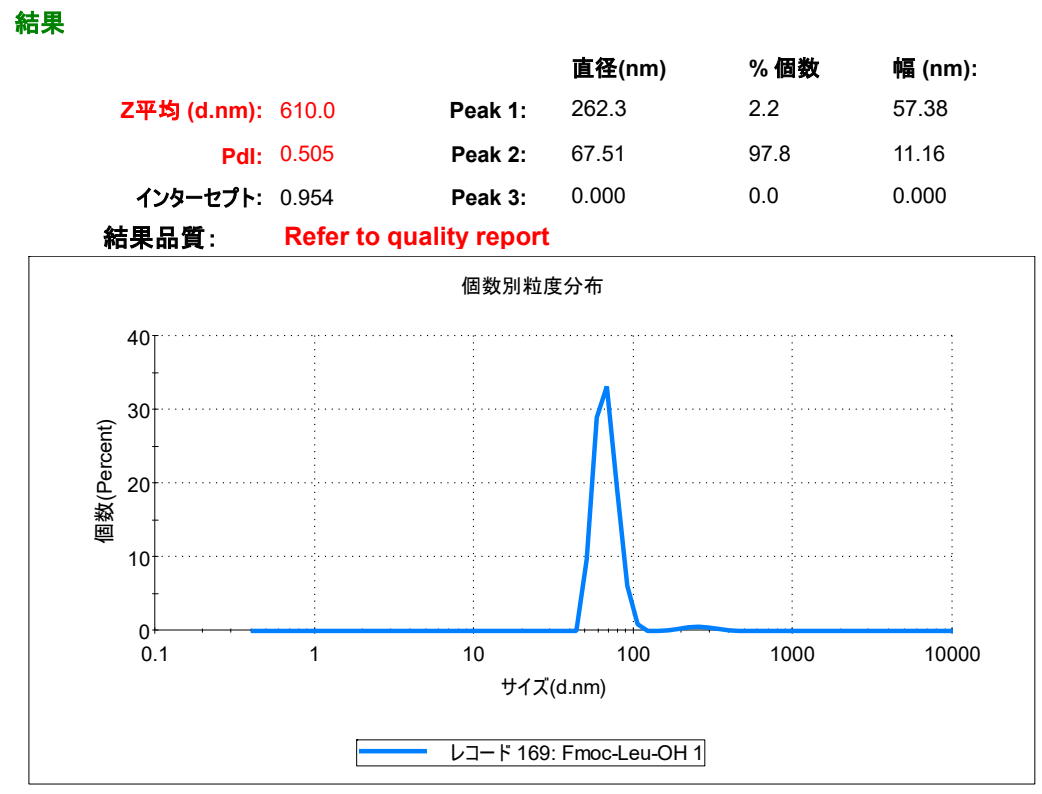

B: Three-Component Nanoassemblies

(a) Intensity-weighted distribution

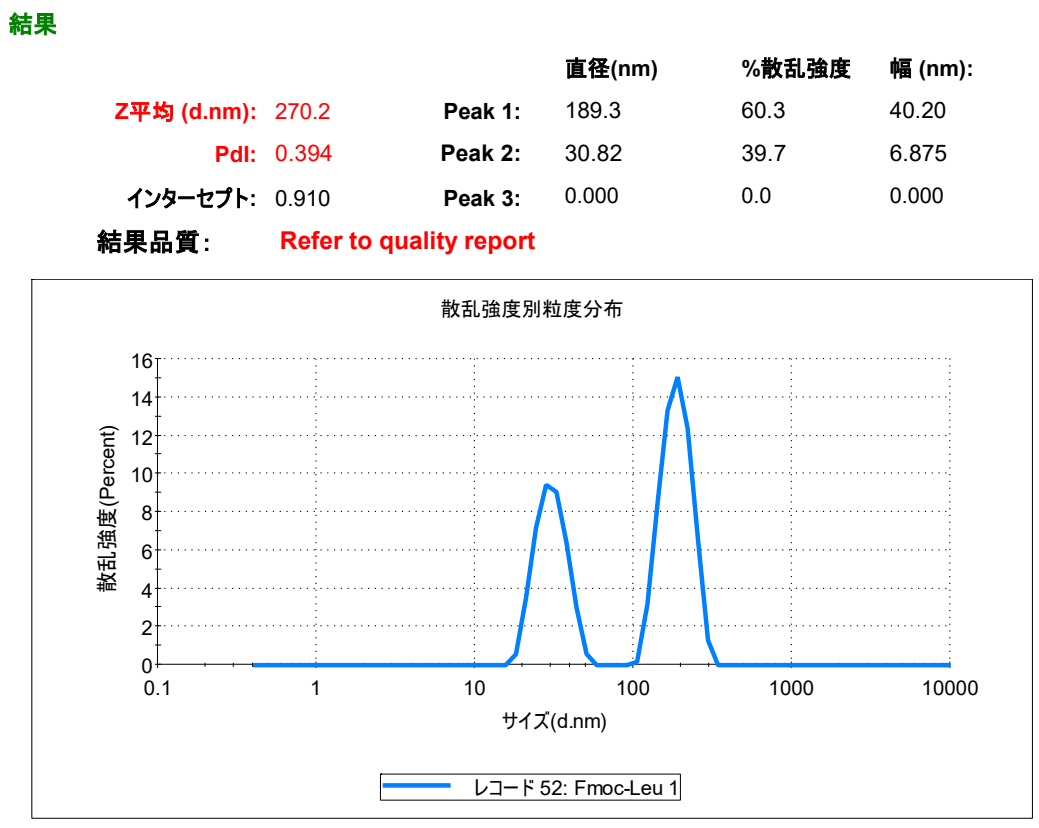

(b) Number-weighted distribution

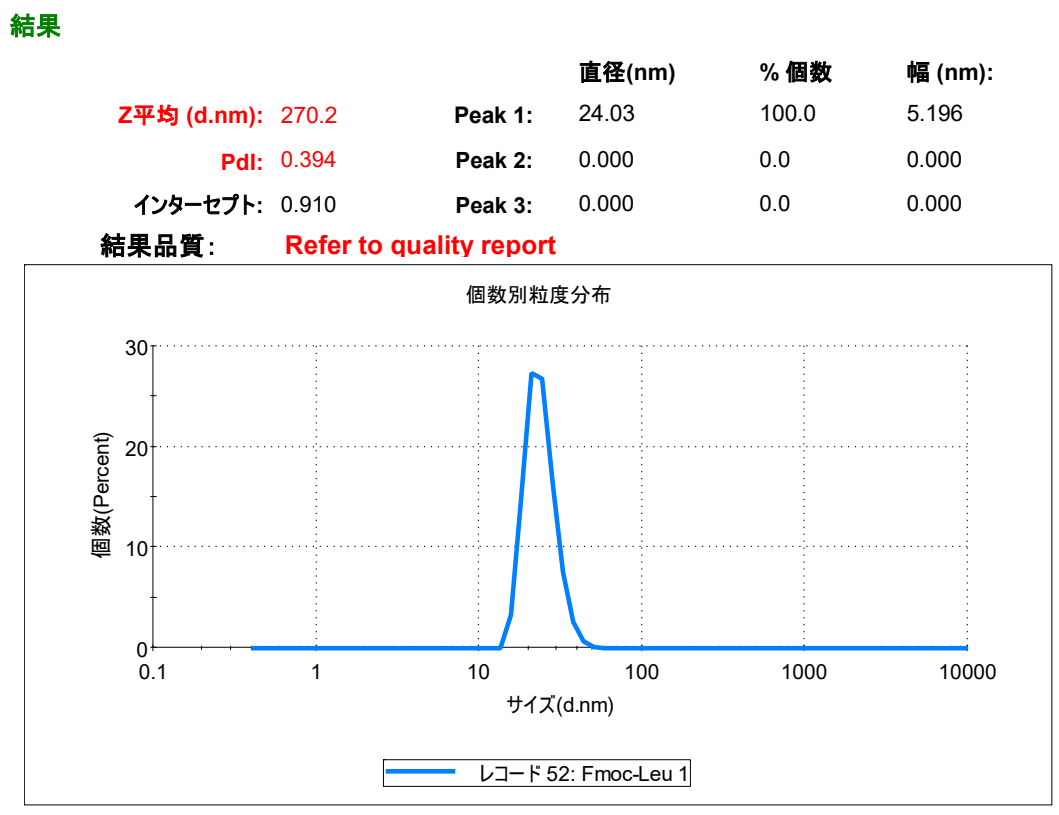

Figure S14. DLS Analysis of Fmoc-Leu-OH Nanoassemblies.

(Japanese labels appearing in the DLS output figures are defined in Table S1.)

A: Two-Component Nanoassemblies (Fmoc-Leu-OH and DIEA).

(a) The intensity-weighted distribution; (b) The corresponding number-weighted distribution.

B: Three-Component Nanoassemblies (Fmoc-Leu-OH, DIEA and TBTU).

(a) The intensity-weighted distribution; (b) The number-weighted distribution.

A: Two-Component Nanoassemblies

(a) Intensity-weighted distribution

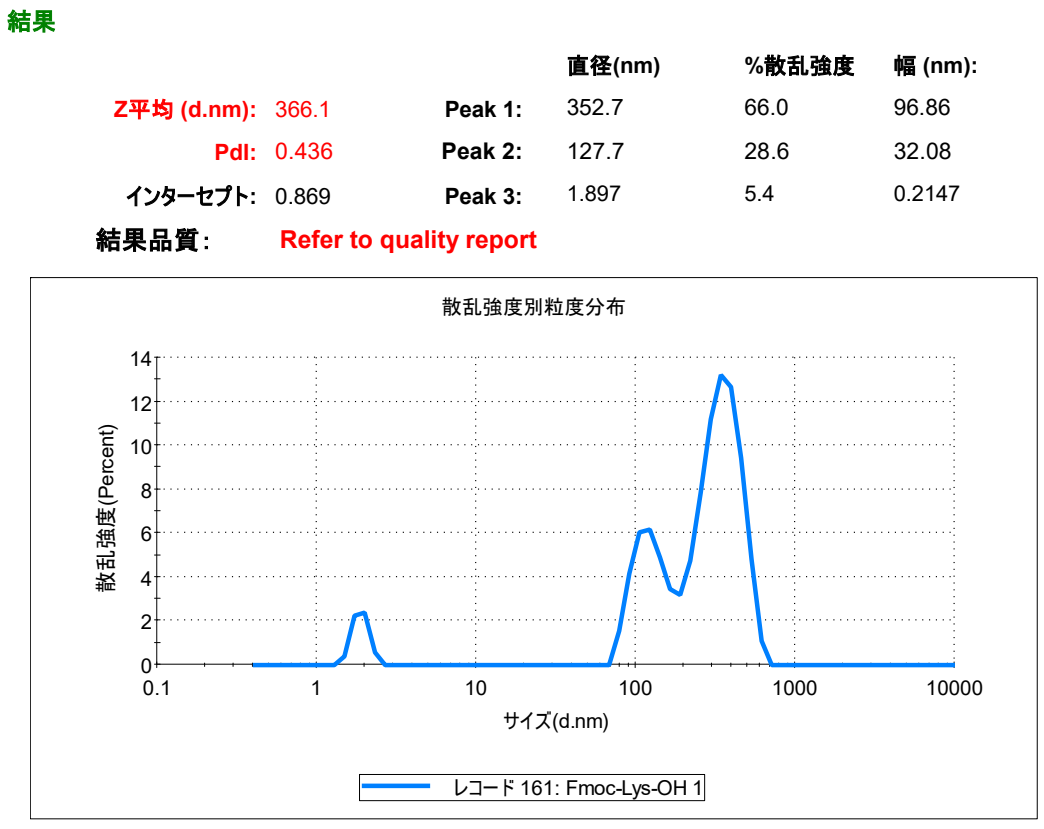

(b) Number-weighted distribution

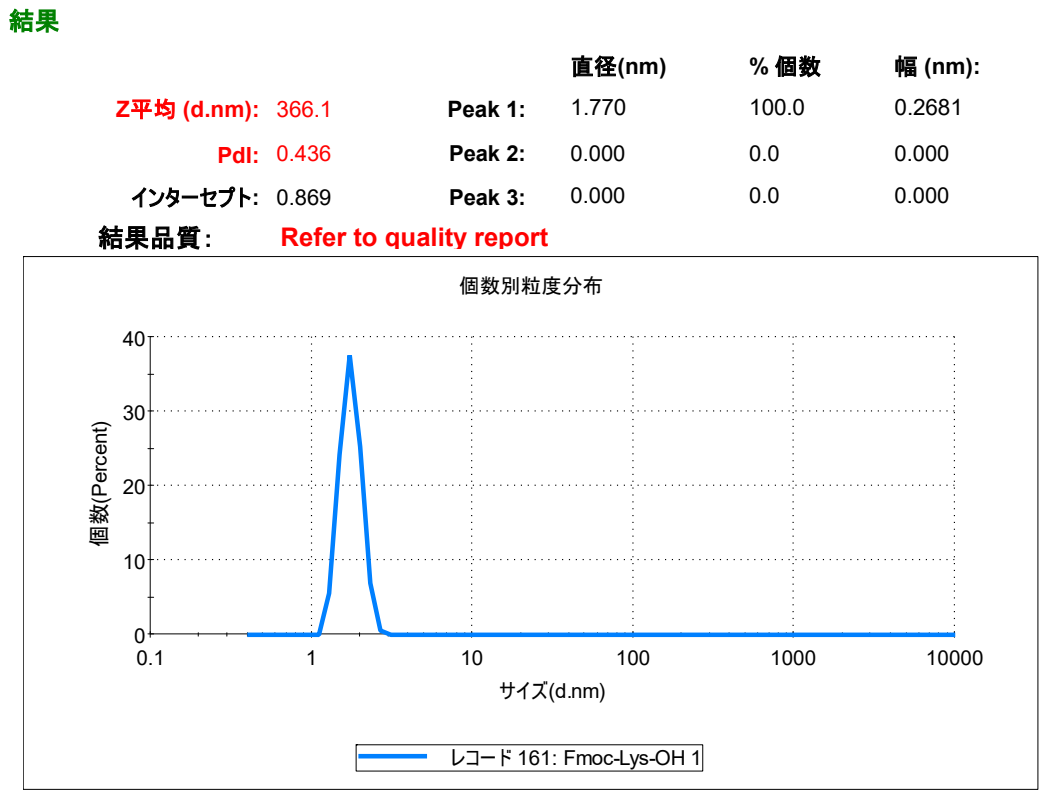

B: Three-Component Nanoassemblies

(a) Intensity-weighted distribution

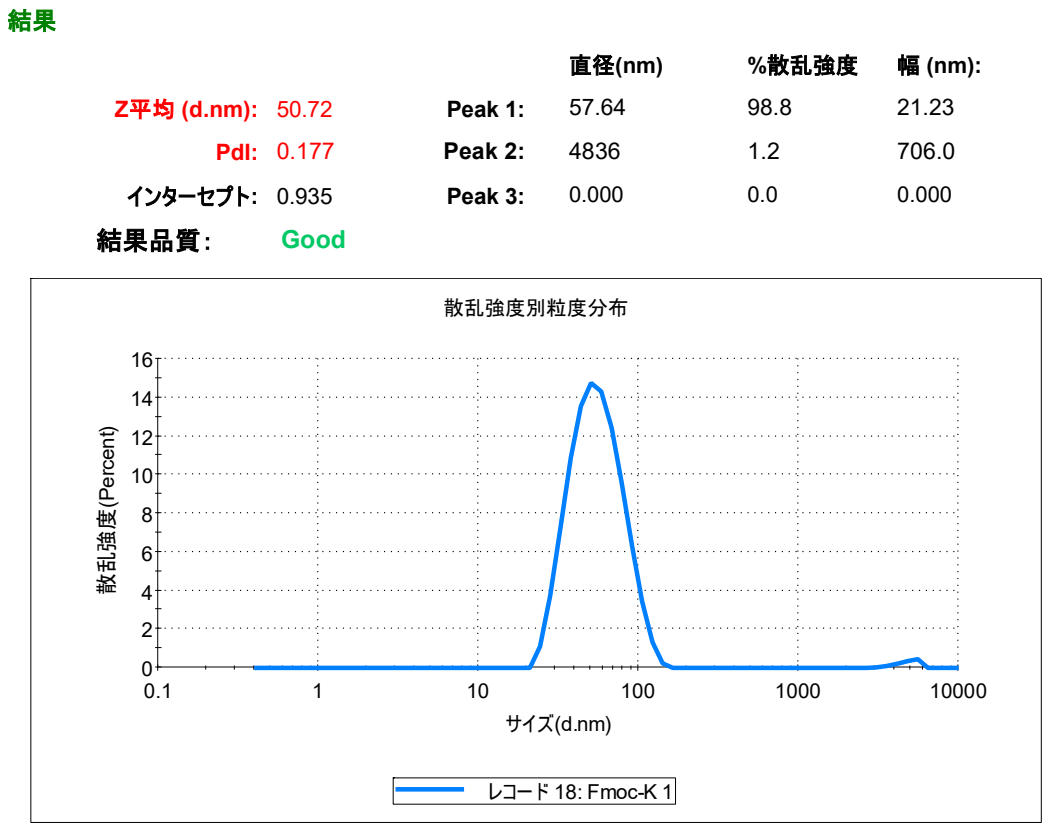

(b) Number-weighted distribution

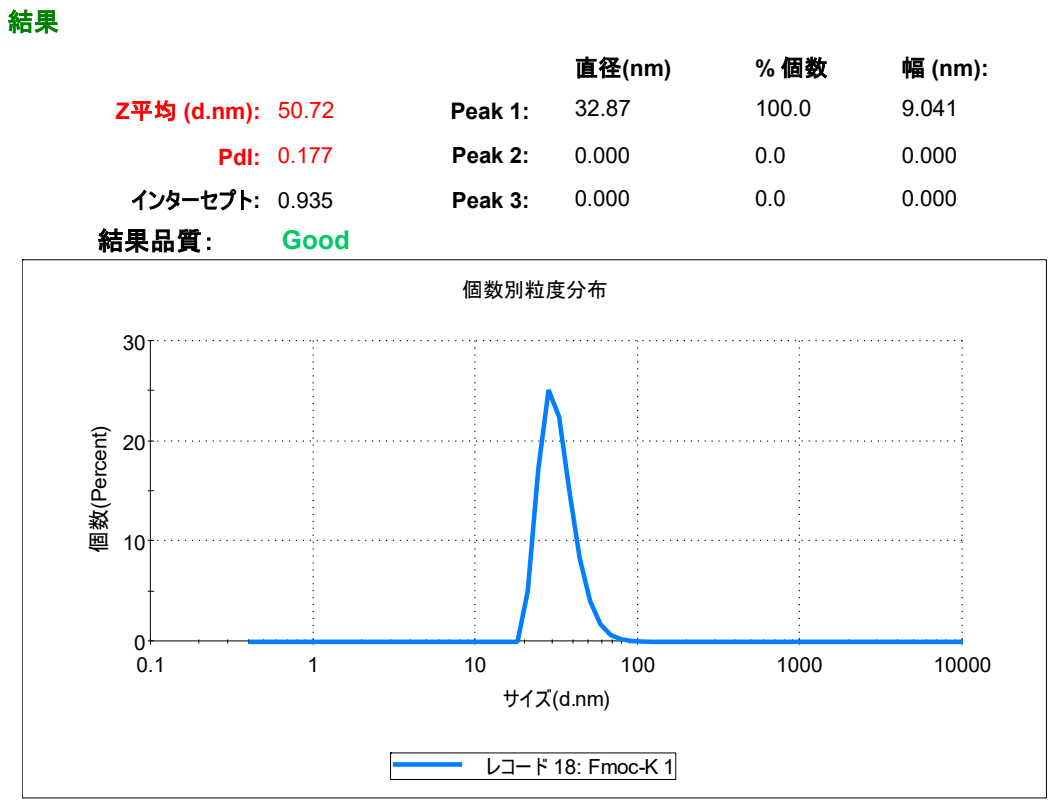

Figure S15. DLS Analysis of Fmoc-Lys(Boc)-OH Nanoassemblies.

(Japanese labels appearing in the DLS output figures are defined in Table S1.)

A: Two-Component Nanoassemblies (Fmoc-Lys(Boc)-OH and DIEA).

(a) The intensity-weighted distribution; (b) The corresponding number-weighted distribution.

B: Three-Component Nanoassemblies (Fmoc-Lys(Boc)-OH, DIEA and TBTU).

(a) The intensity-weighted distribution; (b) The number-weighted distribution.

A: Two-Component Nanoassemblies

(a) Intensity-weighted distribution

結果

			直径(nm)	%散乱強度	幅 (nm):
Z平均 (d.nm):	241.3	Peak 1:	323.6	68.3	102.9
PdI:	0.552	Peak 2:	82.12	31.7	22.88
インターセプト:	0.955	Peak 3:	0.000	0.0	0.000
結果品質:	Refer to quality report				

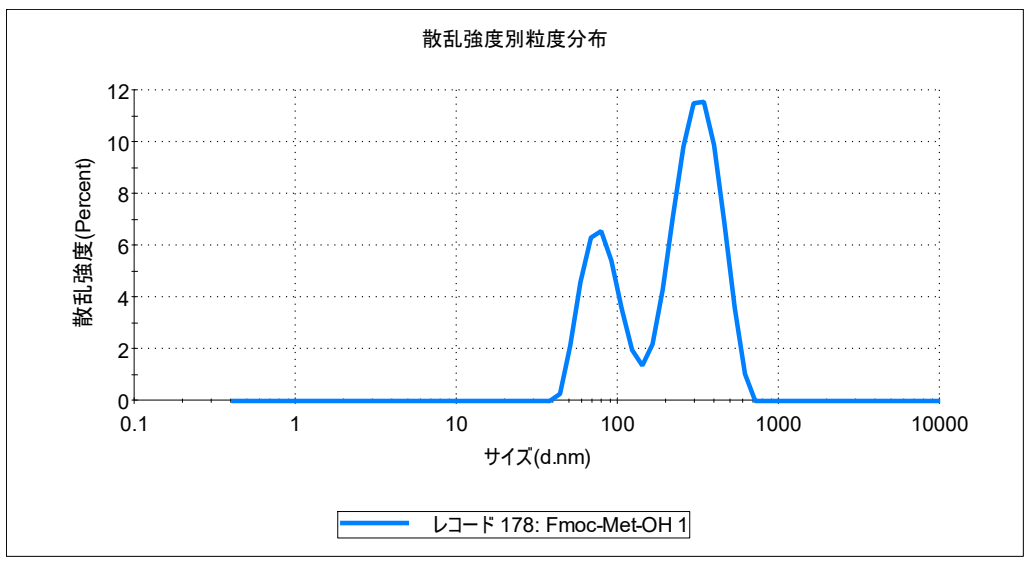

(b) Number-weighted distribution

結果

			直径(nm)	% 個数	幅 (nm):
Z平均 (d.nm):	241.3	Peak 1:	267.9	0.7	90.25
PdI:	0.552	Peak 2:	60.03	99.3	14.37
インターセプト:	0.955	Peak 3:	0.000	0.0	0.000
結果品質:	Refer to quality report				

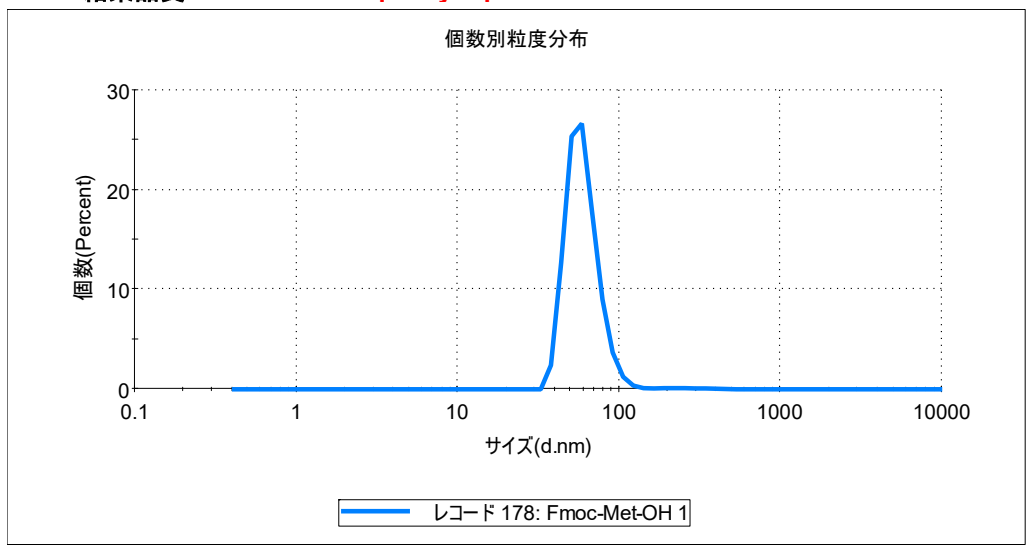

B: Three-Component Nanoassemblies

(a) Intensity-weighted distribution

結果

			直径(nm)	%散乱強度	幅 (nm):
Z平均 (d.nm):	267.6	Peak 1:	329.4	63.1	88.96
PdI:	0.645	Peak 2:	87.21	36.9	27.63
インターセプト:	0.963	Peak 3:	0.000	0.0	0.000
結果品質:	Refer to quality report				

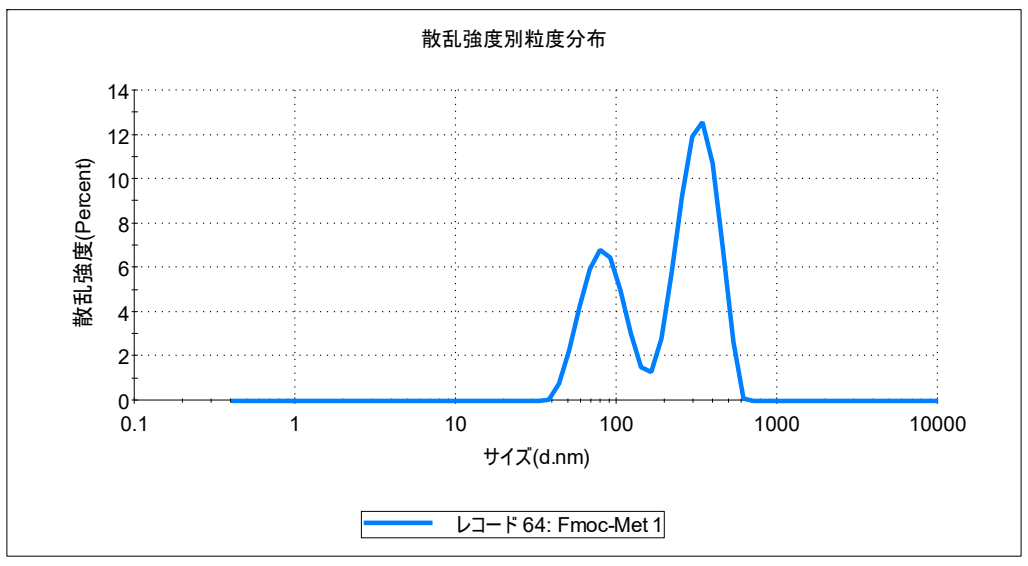

(b) Number-weighted distribution

結果

			直径(nm)	% 個数	幅 (nm):
Z平均 (d.nm):	267.6	Peak 1:	277.4	0.5	88.02
PdI:	0.645	Peak 2:	57.21	99.5	15.28
インターセプト:	0.963	Peak 3:	0.000	0.0	0.000
結果品質:	Refer to quality report				

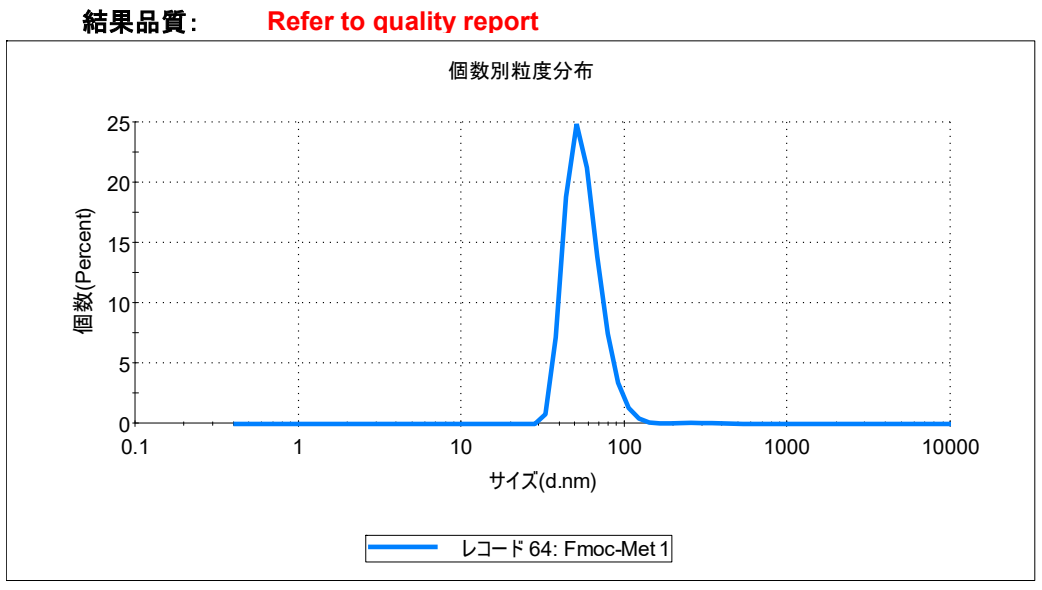

Figure S16. DLS Analysis of Fmoc-Met-OH Nanoassemblies.

(Japanese labels appearing in the DLS output figures are defined in Table S1.)

A: Two-Component Nanoassemblies (Fmoc-Met-OH and DIEA).

(a) The intensity-weighted distribution; (b) The corresponding number-weighted distribution.

B: Three-Component Nanoassemblies (Fmoc-Met-OH, DIEA and TBTU).

(a) The intensity-weighted distribution; (b) The number-weighted distribution.

A: Two-Component Nanoassemblies

(a) Intensity-weighted distribution

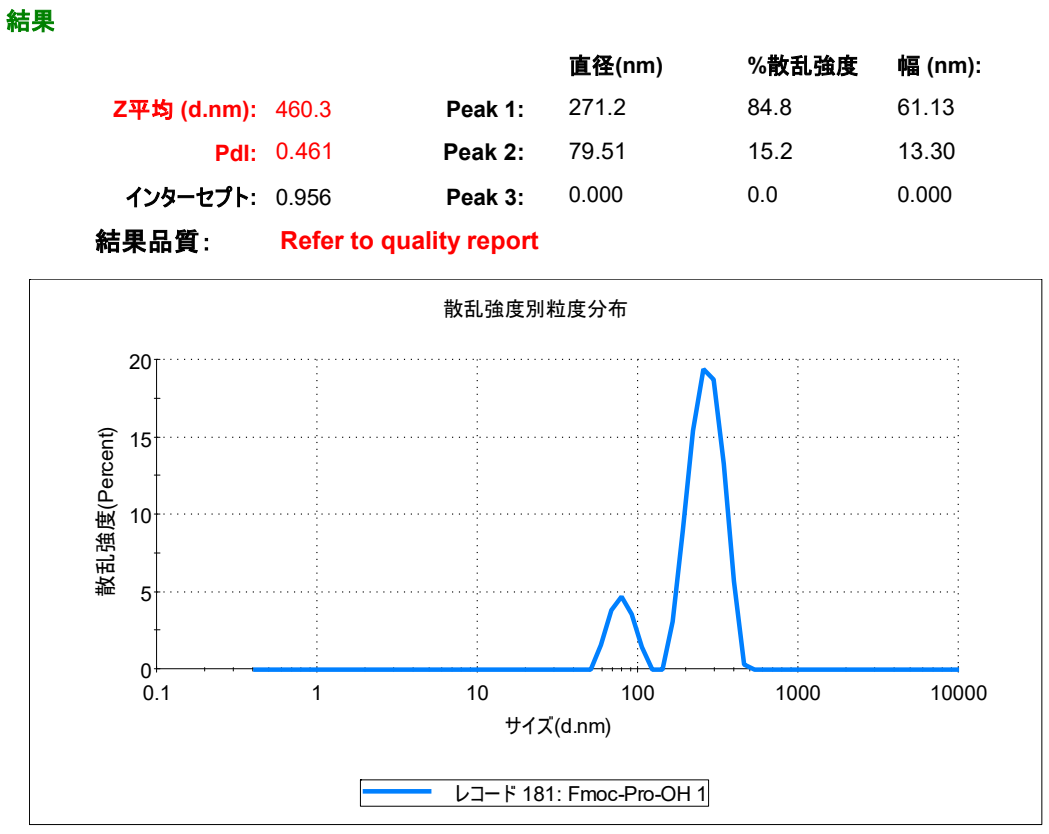

(b) Number-weighted distribution

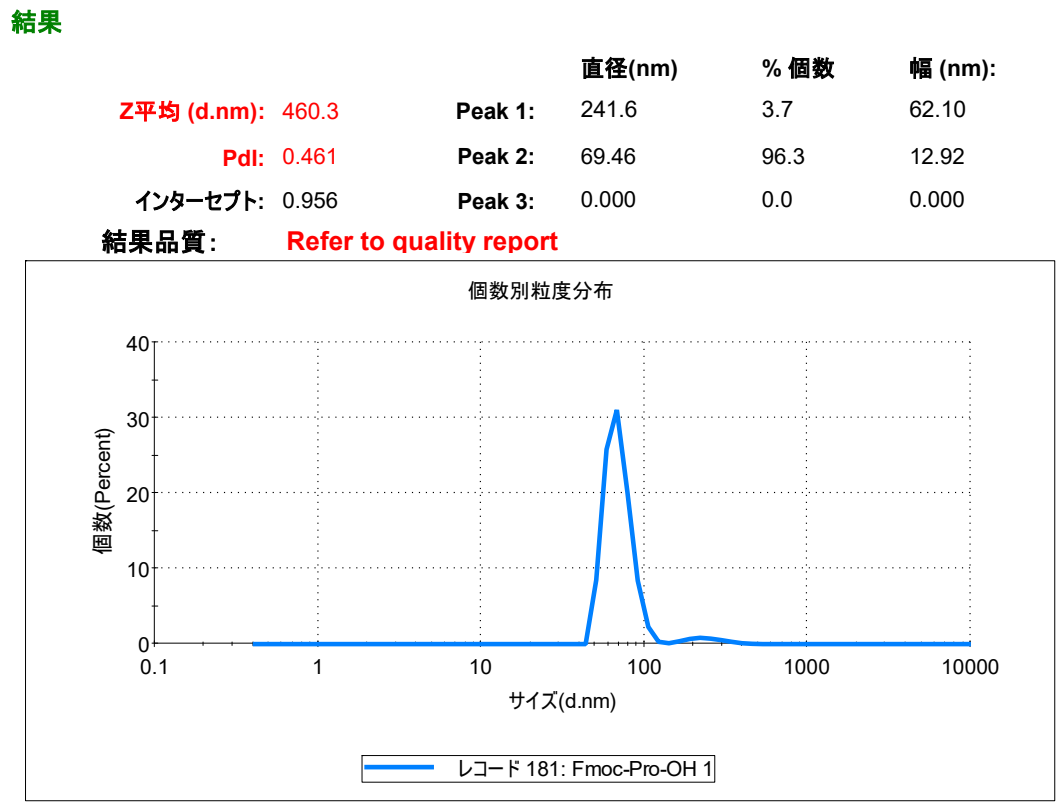

B: Three-Component Nanoassemblies

(a) Intensity-weighted distribution

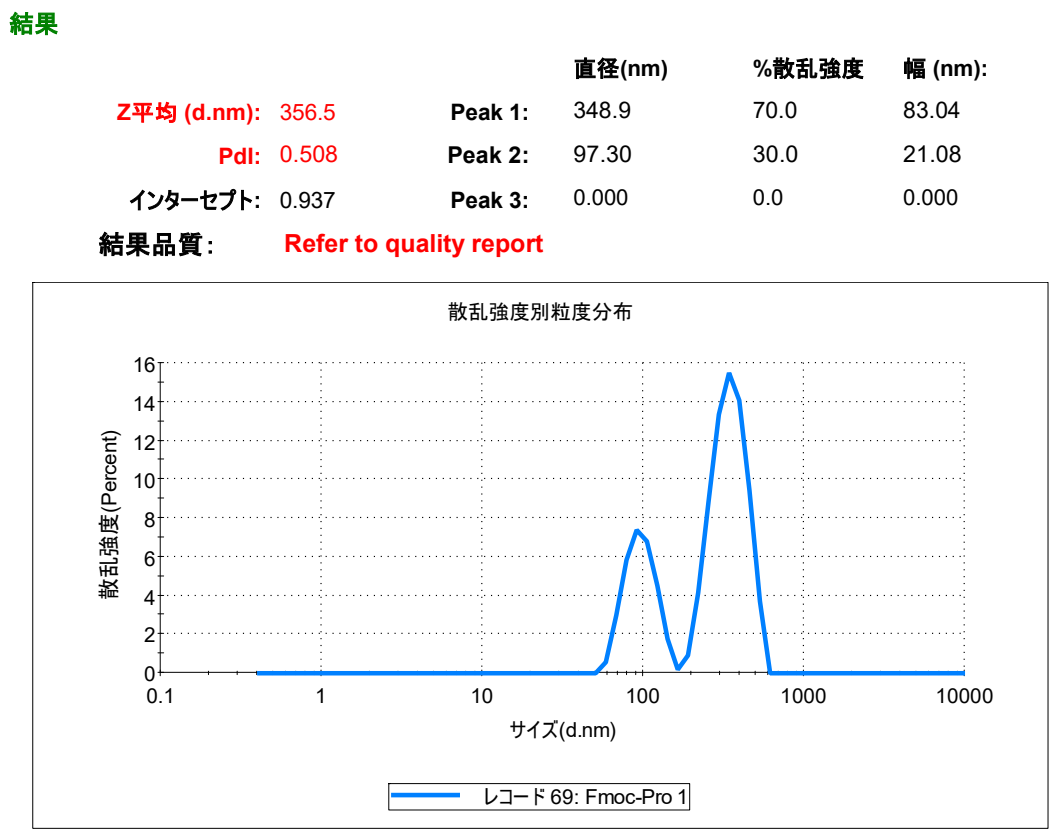

(b) Number-weighted distribution

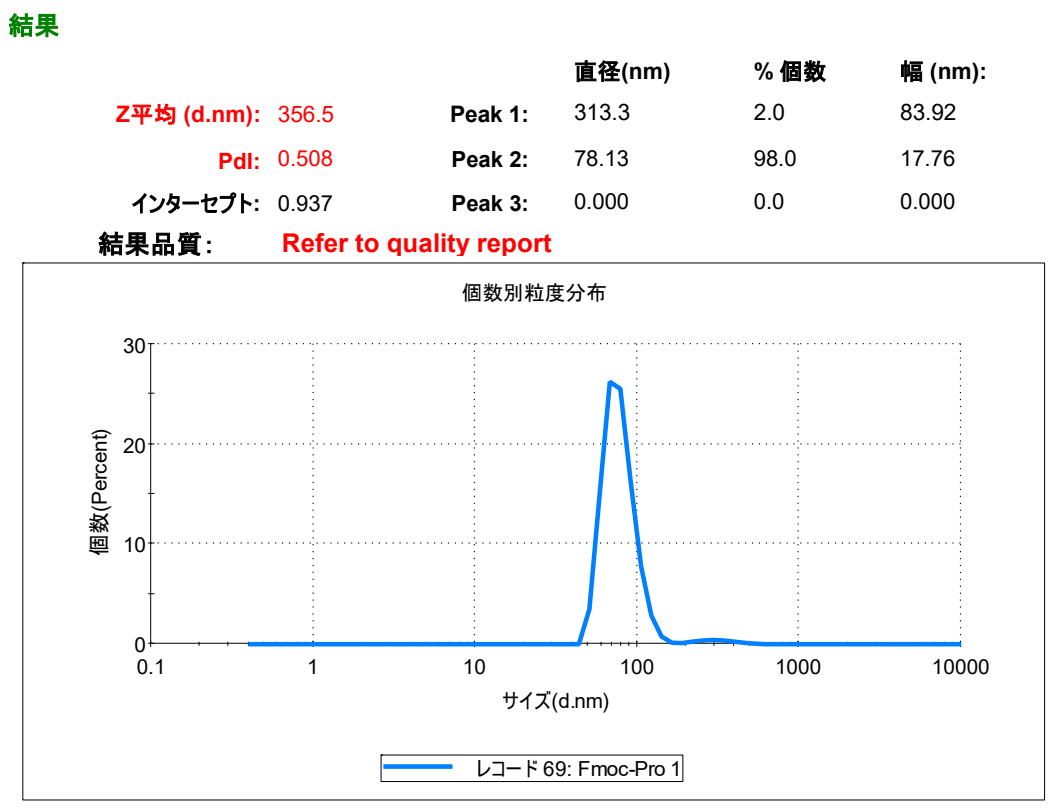

Figure S17. DLS Analysis of Fmoc-Pro-OH Nanoassemblies.

(Japanese labels appearing in the DLS output figures are defined in Table S1.)

A: Two-Component Nanoassemblies (Fmoc-Pro-OH and DIEA).

(a) The intensity-weighted distribution; (b) The corresponding number-weighted distribution.

B: Three-Component Nanoassemblies (Fmoc-Pro-OH, DIEA and TBTU).

(a) The intensity-weighted distribution; (b) The corresponding number-weighted distribution.

A: Two-Component Nanoassemblies

(a) Intensity-weighted distribution

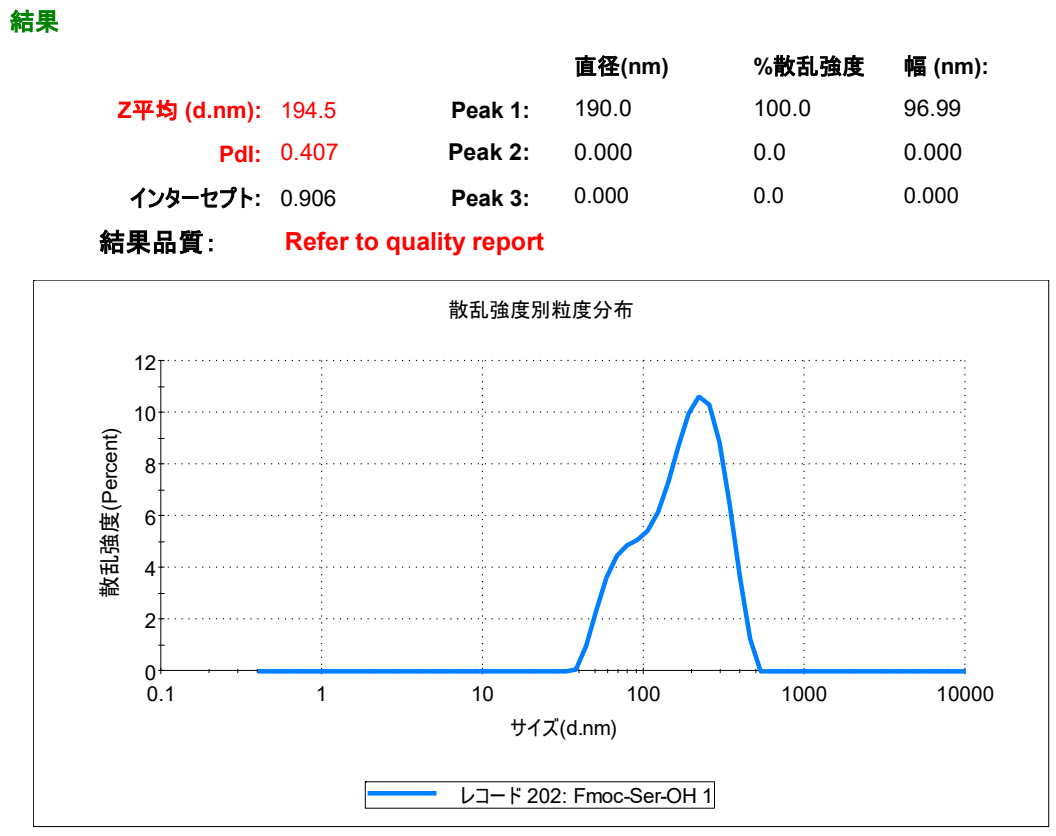

(b) Number-weighted distribution

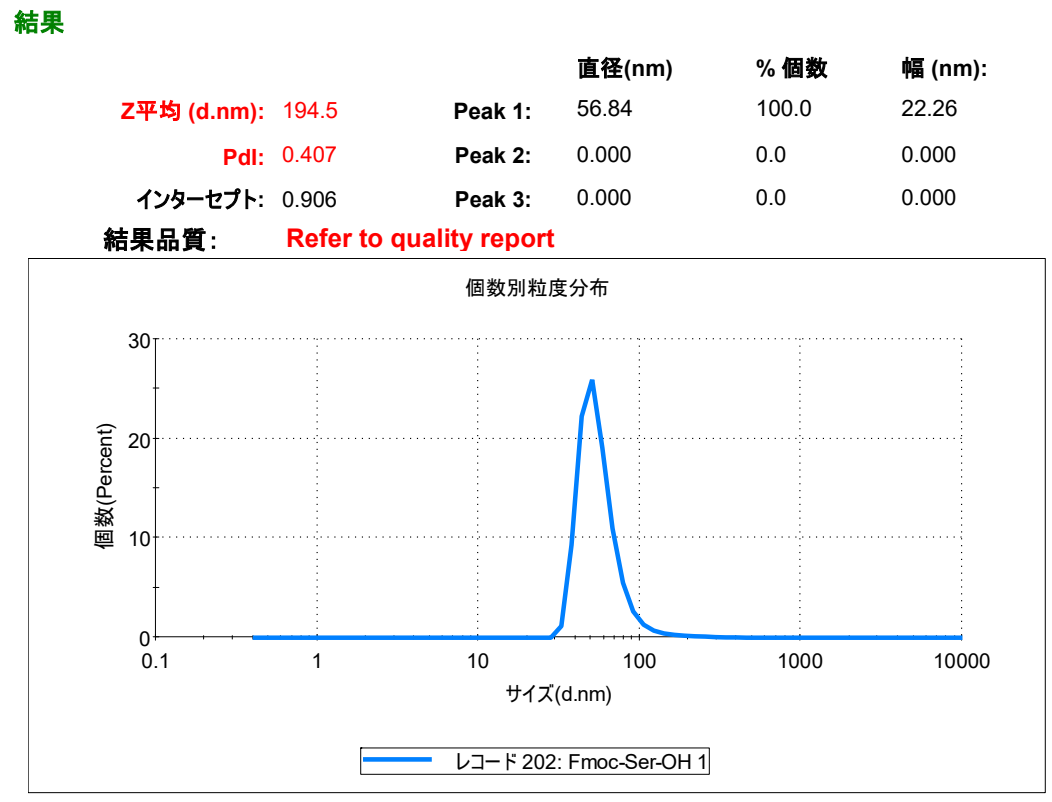

B: Three-Component Nanoassemblies

(a) Intensity-weighted distribution

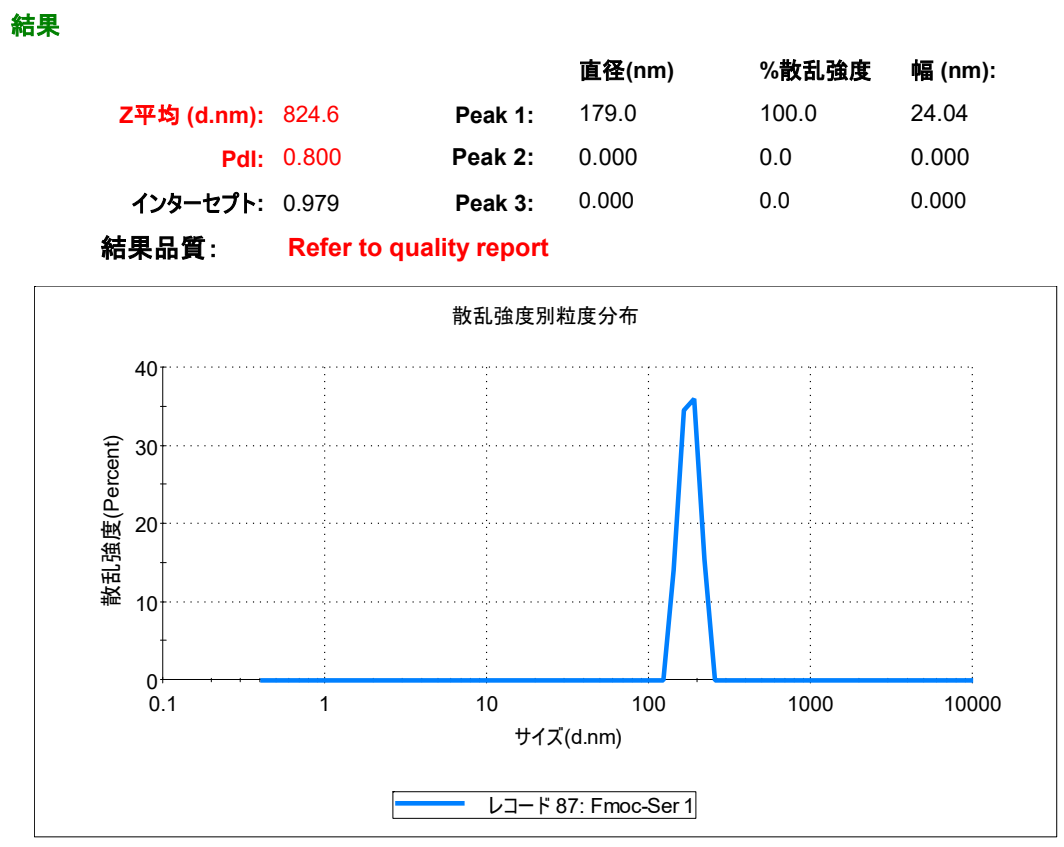

(b) Number-weighted distribution

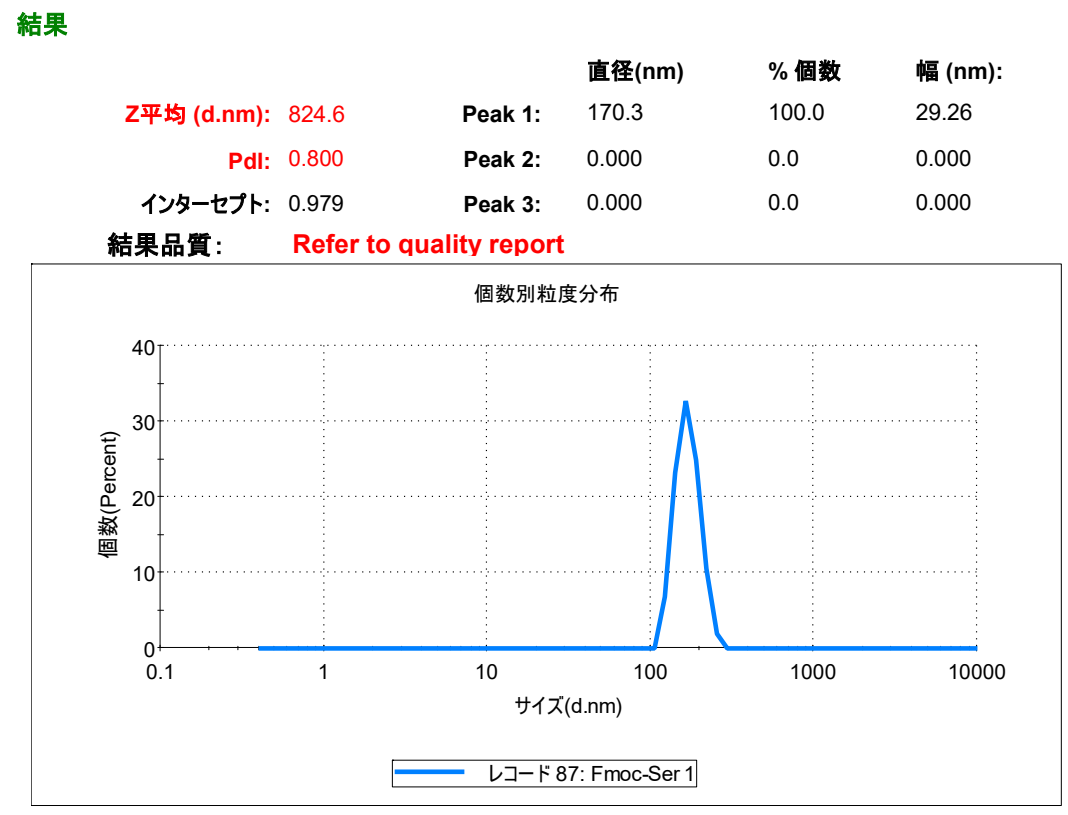

Figure S18. DLS Analysis of Fmoc-Ser(tBu)-OH Nanoassemblies.

(Japanese labels appearing in the DLS output figures are defined in Table S1.)

A: Two-Component Nanoassemblies (Fmoc-Ser(tBu)-OH and DIEA).

(a) The intensity-weighted distribution; (b) The corresponding number-weighted distribution.

B: Three-Component Nanoassemblies (Fmoc-Ser(tBu)-OH, DIEA and TBTU).

(a) The intensity-weighted distribution; (b) The corresponding number-weighted distribution.

A: Two-Component Nanoassemblies

(a) Intensity-weighted distribution

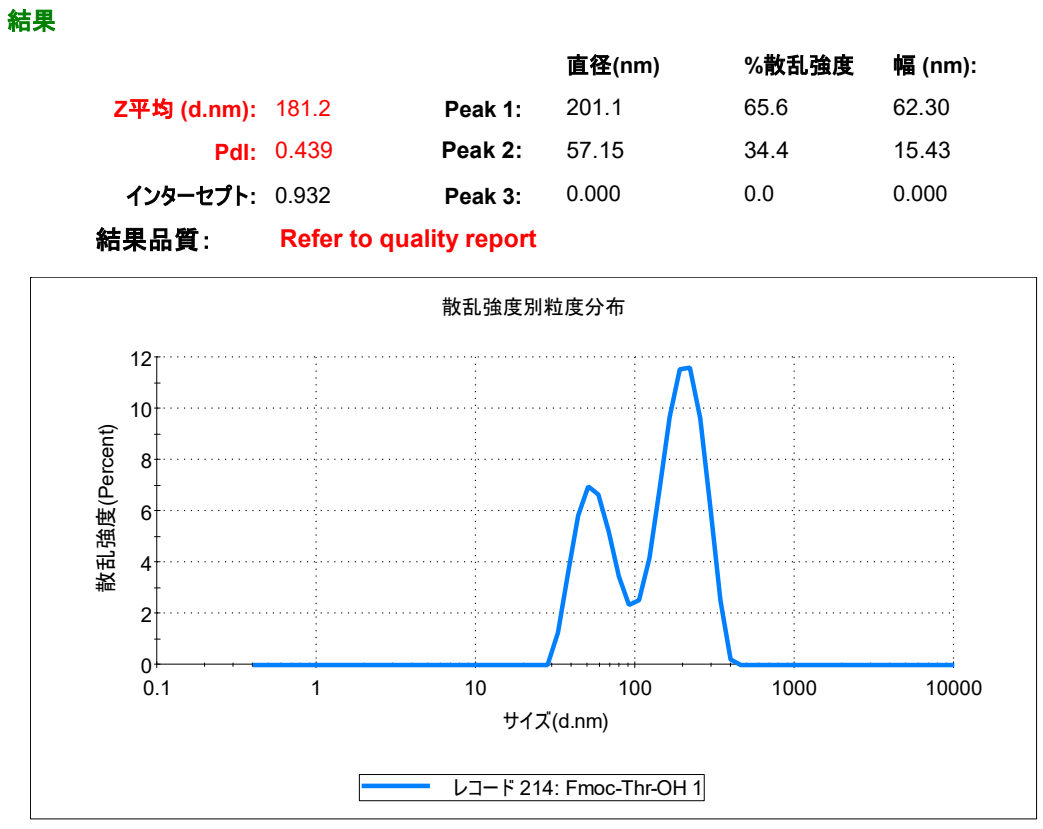

(b) Number-weighted distribution

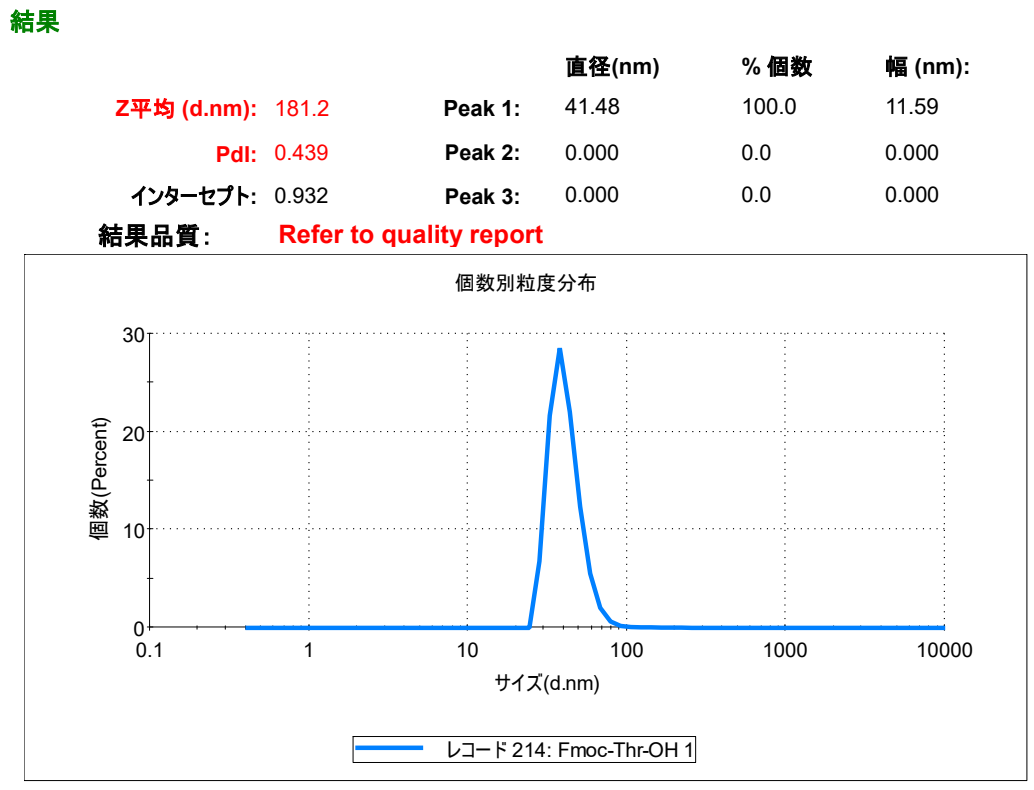

B: Three-Component Nanoassemblies

(a) Intensity-weighted distribution

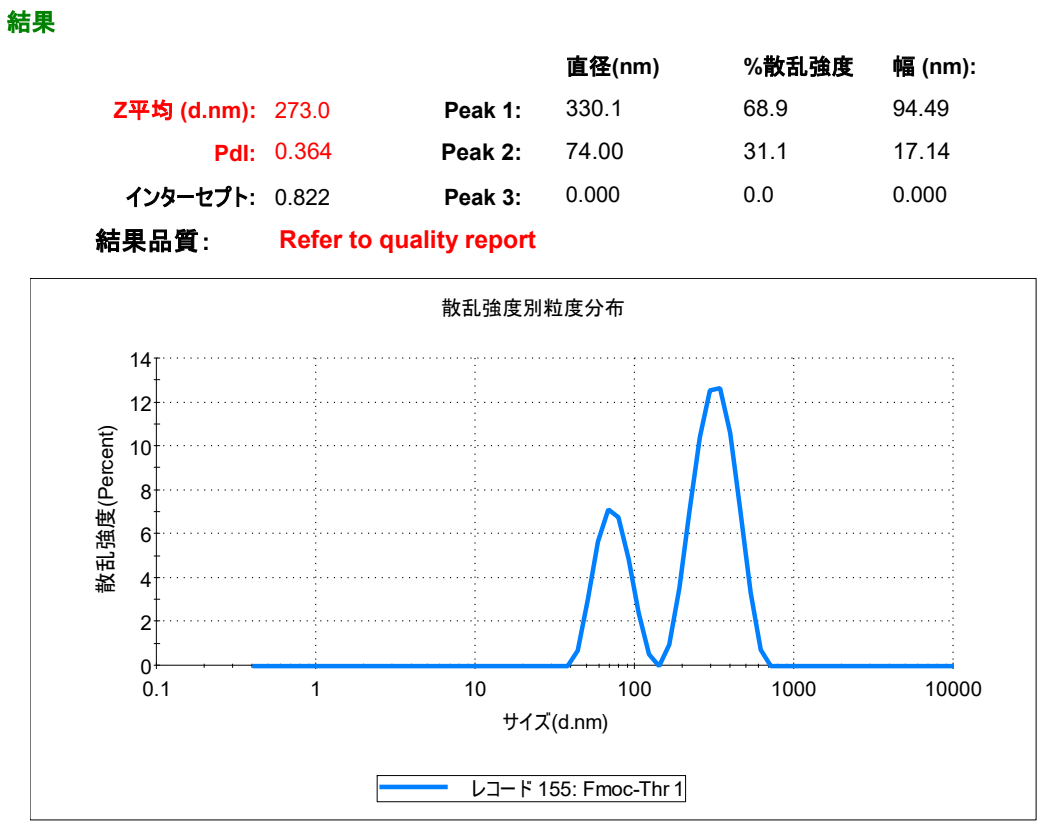

(b) Number-weighted distribution

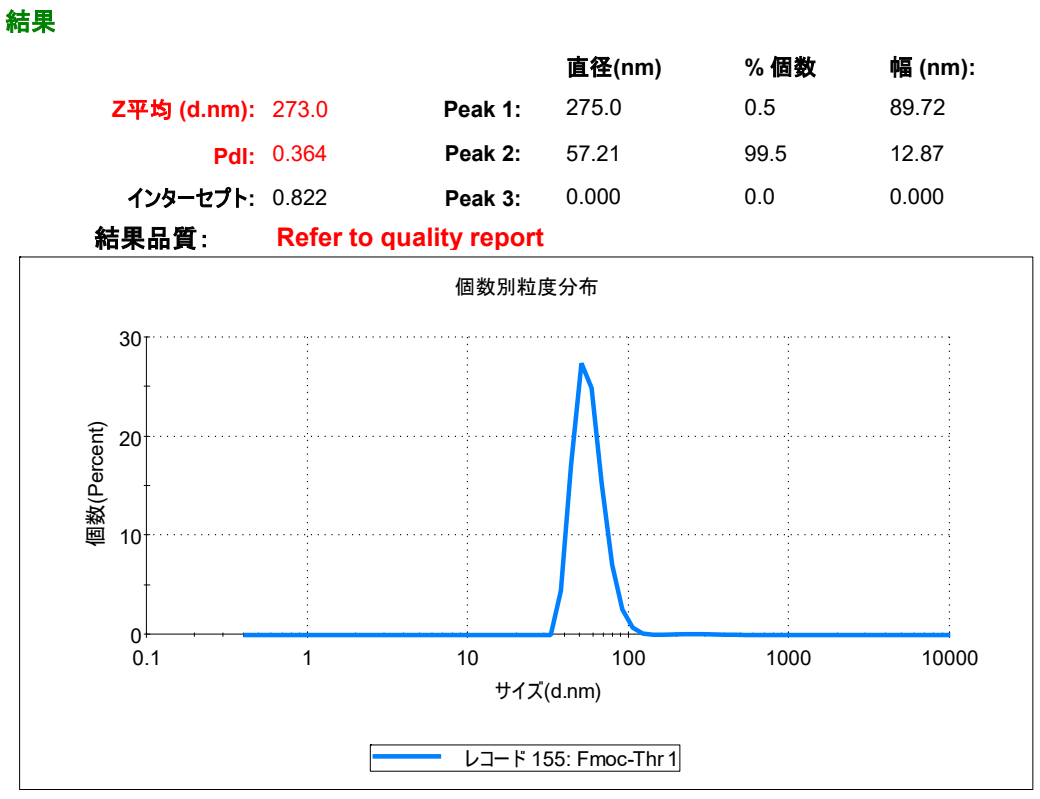

Figure S19. DLS Analysis of Fmoc-Thr(tBu)-OH Nanoassemblies.

(Japanese labels appearing in the DLS output figures are defined in Table S1.)

A: Two-Component Nanoassemblies (Fmoc-Thr(tBu)-OH and DIEA).

(a) The intensity-weighted distribution; (b) The corresponding number-weighted distribution.

B: Three-Component Nanoassemblies (Fmoc-Thr(tBu)-OH, DIEA and TBTU).

(a) The intensity-weighted distribution; (b) The corresponding number-weighted distribution.

A: Two-Component Nanoassemblies

(a) Intensity-weighted distribution

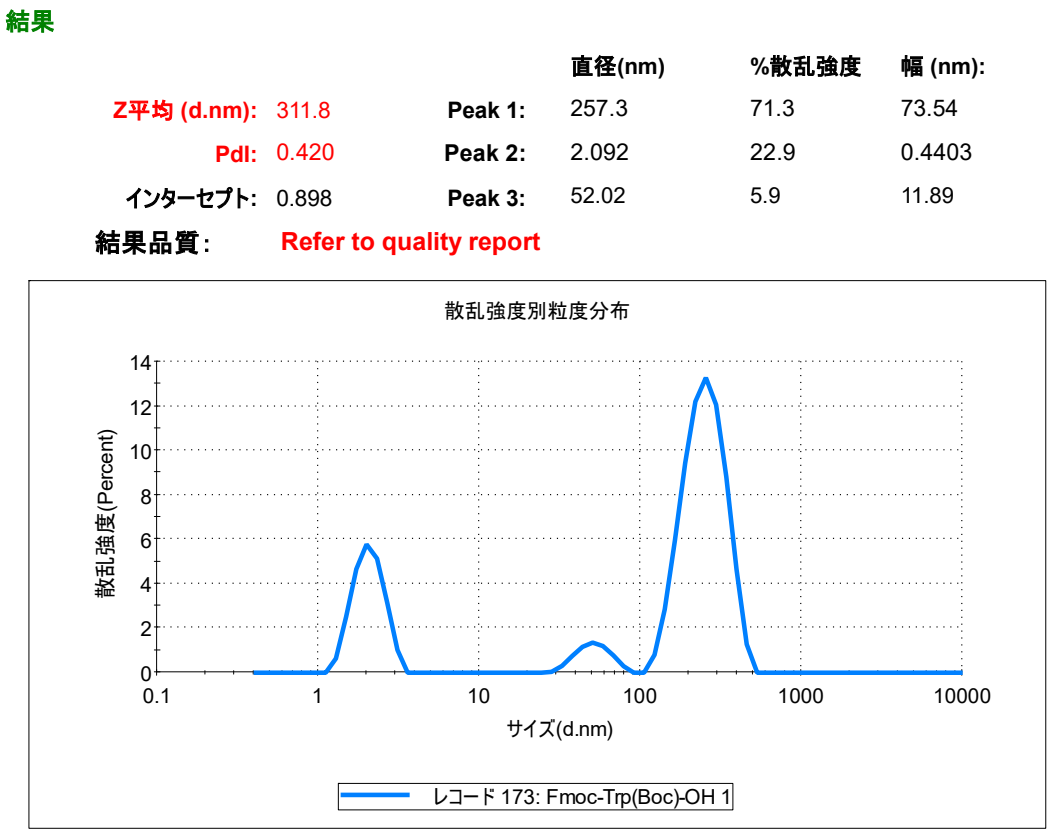

(b) Number-weighted distribution

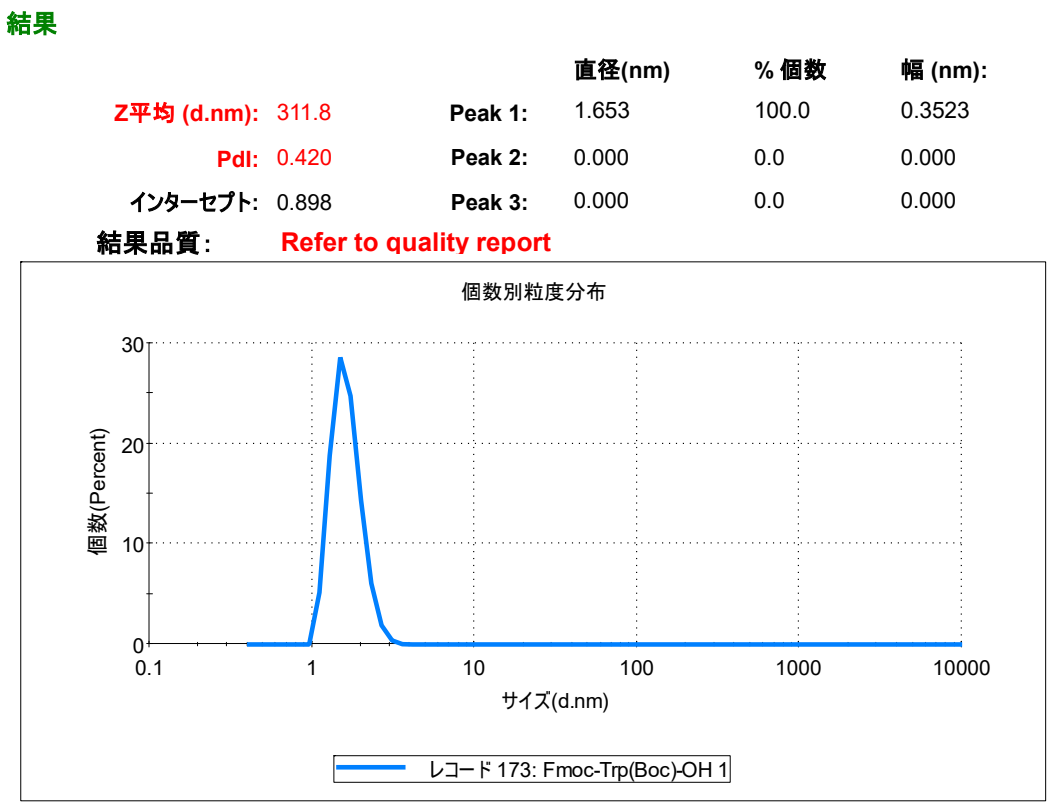

B: Three-Component Nanoassemblies

(a) Intensity-weighted distribution

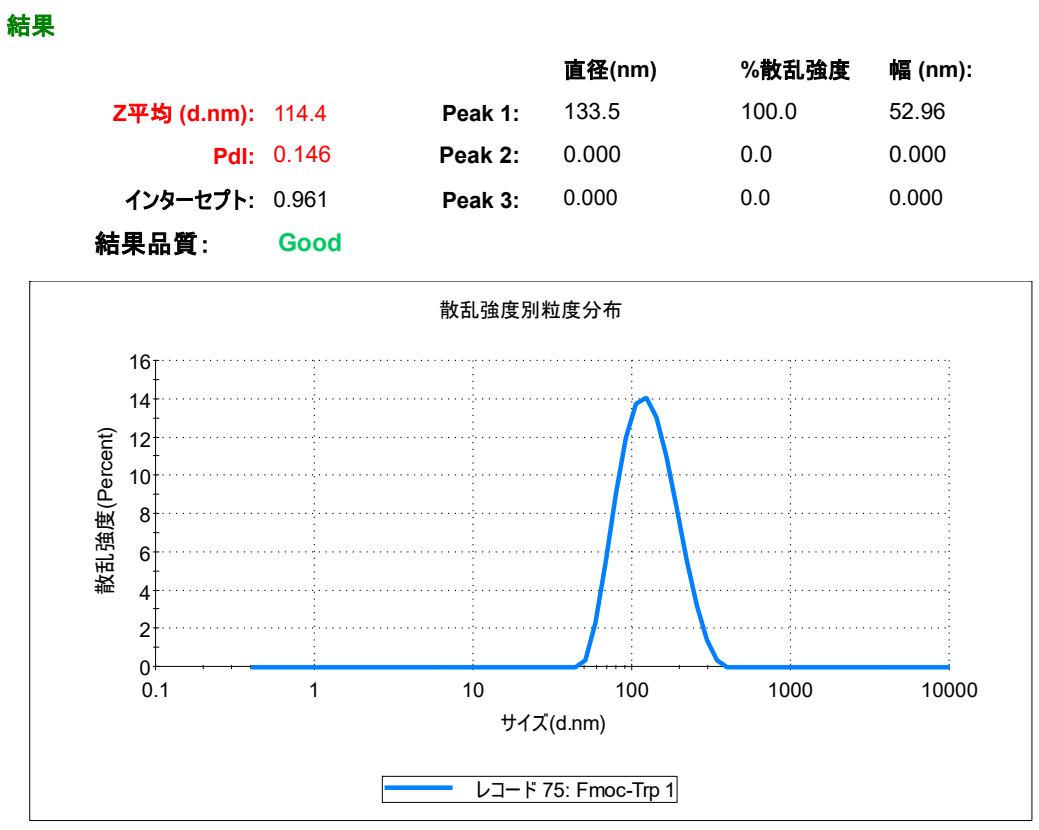

(b) Number-weighted distribution

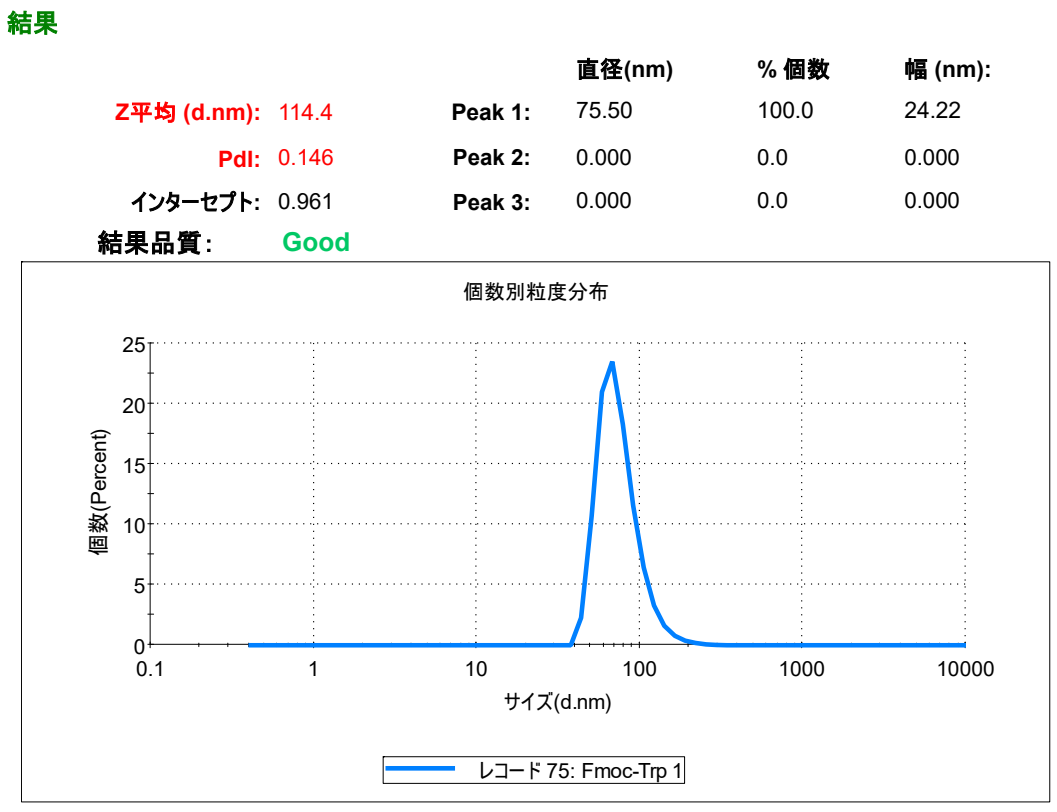

Figure S20. DLS Analysis of Fmoc-Trp(Boc)-OH Nanoassemblies.

(Japanese labels appearing in the DLS output figures are defined in Table S1.)

A: Two-Component Nanoassemblies (Fmoc-Trp(Boc)-OH and DIEA).

(a) The intensity-weighted distribution; (b) The corresponding number-weighted distribution.

B: Three-Component Nanoassemblies (Fmoc-Trp(Boc)-OH, DIEA and TBTU).

(a) The intensity-weighted distribution; (b) The corresponding number-weighted distribution.

A: Two-Component Nanoassemblies

(a) Intensity-weighted distribution

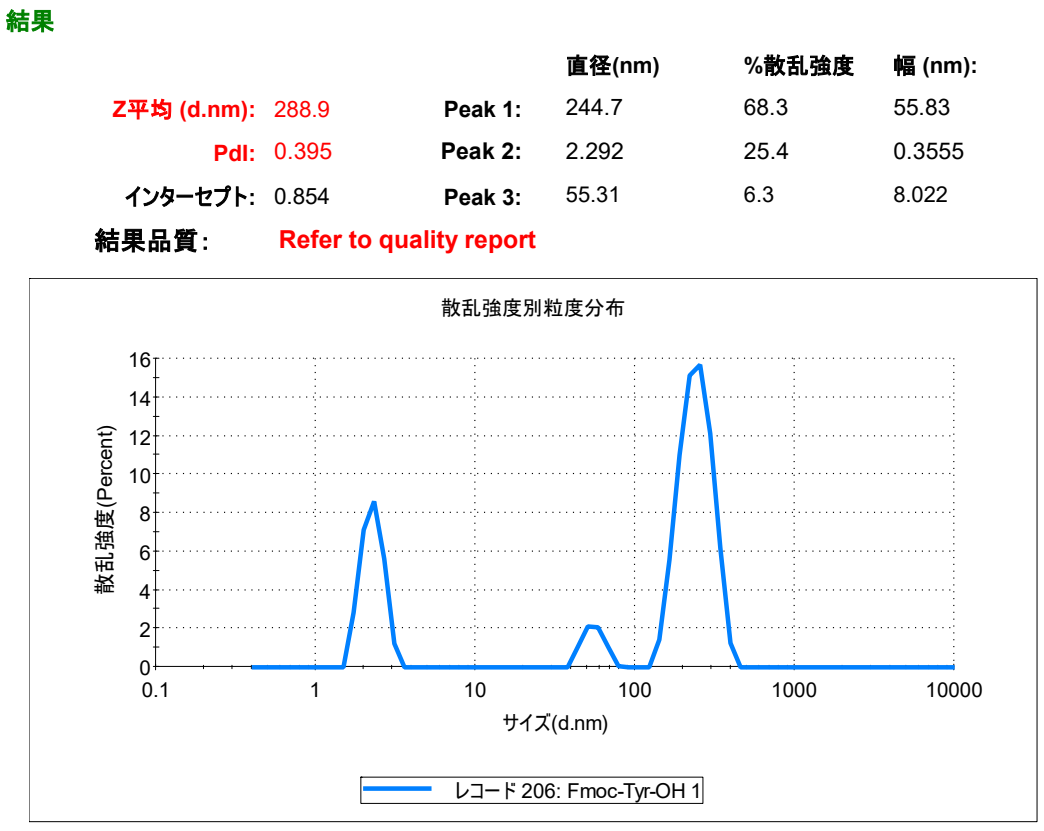

(b) Number-weighted distribution

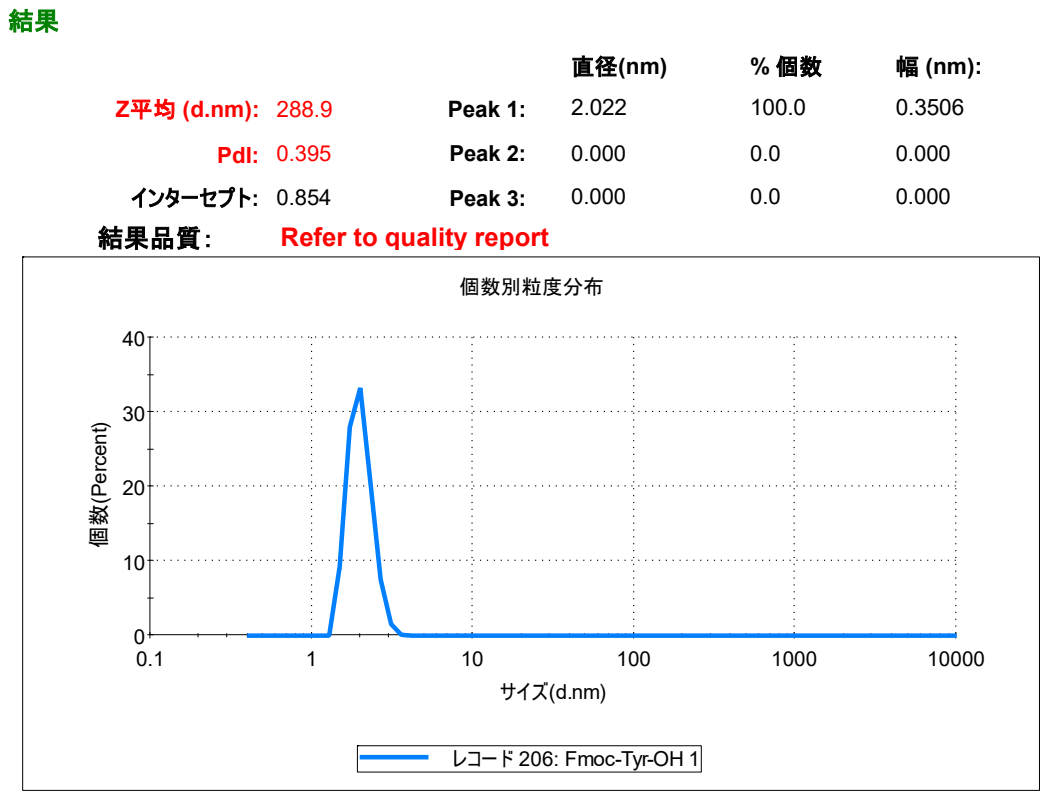

B: Three-Component Nanoassemblies

(a) Intensity-weighted distribution

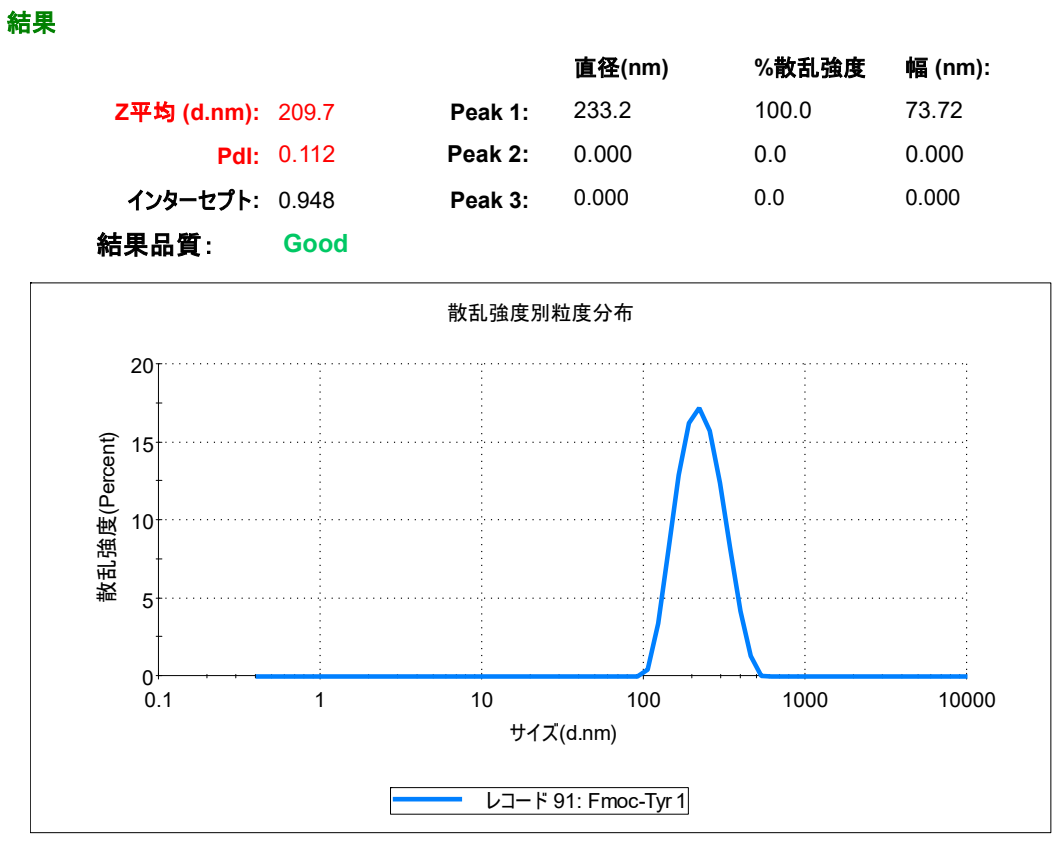

(b) Number-weighted distribution

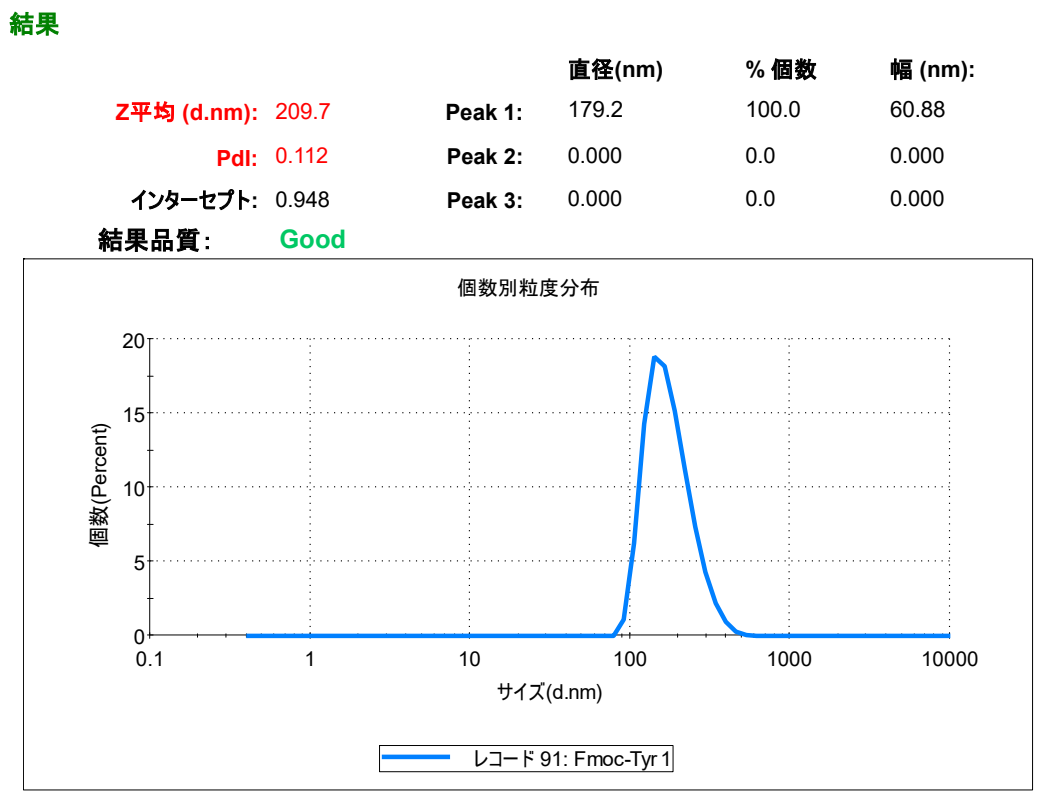

Figure S21. DLS Analysis of Fmoc-Tyr(tBu)-OH Nanoassemblies.

(Japanese labels appearing in the DLS output figures are defined in Table S1.)

A: Two-Component Nanoassemblies (Fmoc-Tyr(tBu)-OH and DIEA).

(a) The intensity-weighted distribution; (b) The corresponding number-weighted distribution.

B: Three-Component Nanoassemblies (Fmoc-Tyr(tBu)-OH, DIEA and TBTU).

(a) The intensity-weighted distribution; (b) The corresponding number-weighted distribution.

S1.4. Particle Size of Nanoassemblies

Table S2. Mode Diameter (Intensity-Weighted) of Fmoc-Amino Acid Nanoassemblies.

Fmoc-amino acid	Two-component nanoassemblies (Fmoc-AA-OH/DIEA=1:1.2) Mode diameter (nm) (PDI)	Three-component nanoassemblies (Fmoc-AA-OH/DIEA/TBTU=1:1.2:1) Mode diameter (nm) (PDI)
Fmoc-Ala-OH	187.1 ± 39.53 (0.427)	274.0 ± 77.41 (0.469)
Fmoc-Asn(Trt)-OH*	317.7 ± 112.9 (0.443)	120.4 ± 46.44 (0.231)
Fmoc-Asp(OtBu)-OH	183.0 ± 80.24 (0.297)	158.2 ± 17.97 (0.748)
Fmoc-Arg(Pbf)-OH*	267.0 ± 110.0 (0.365)	217.3 ± 57.15 (0.060)
Fmoc-Cys(Trt)-OH*	17.14 ± 65.23 (0.136)	75.59 ± 20.75 (0.076)
Fmoc-Gln(Trt)-OH*	432.2 ± 156.3 (0.526)	45.60 ± 116.8 (0.181)
Fmoc-Glu(OtBu)-OH	328.4 ± 113.3 (0.448)	320.3 ± 67.90 (0.518)
Fmoc-Gly-OH	89.07 ± 11.29 (0.348)	292.0 ± 76.13 (0.472)
Fmoc-His(Boc)-OH	302.9 ± 128.1 (0.850)	54.84 ± 19.34 (0.228)
Fmoc-Ile-OH	159.5 ± 24.45 (0.420)	182.9 ± 41.38 (0.342)
Fmoc-Leu-OH	282.5 ± 52.70 (0.505)	189.3 ± 40.20 (0.394)
Fmoc-Lys(Boc)-OH	352.7 ± 96.86 (0.438)	57.64 ± 21.23 (0.177)
Fmoc-Met-OH	323.6 ± 102.9 (0.552)	329.4 ± 88.96 (0.645)
Fmoc-Phe-OH	255.9 ± 67.92 (0.385)	66.73 ± 23.43 (0.195)
Fmoc-Pro-OH	271.2 ± 61.13 (0.461)	48.90 ± 83.04 (0.508)
Fmoc-Ser(tBu)-OH	190.0 ± 96.99 (0.407)	179.0 ± 24.04 (0.800)
Fmoc-Thr(tBu)-OH	201.1 ± 62.30 (0.439)	330.1 ± 94.49 (0.364)
Fmoc-Trp(Boc)-OH	257.3 ± 73.54 (0.420)	133.5 ± 52.96 (0.146)
Fmoc-Tyr(tBu)-OH	244.7 ± 55.83 (0.395)	233.2 ± 73.72 (0.112)
Fmoc-Val-OH	212.7 ± 56.77 (0.061)	249.8 ± 94.48 (0.571)

* Aqueous 10% *i*-PrOH solution was used for preparation.

Table S3. Mode Diameter (Number-Weighted) of Fmoc-Amino Acid Nanoassemblies.

Fmoc-amino acid	Two-component nanoassemblies (Fmoc-AA-OH/DIEA=1:1.2) Mode diameter (nm) (PDI)	Three-component nanoassemblies (Fmoc-AA-OH/DIEA/TBTU=1:1.2:1) Mode diameter (nm) (PDI)
Fmoc-Ala-OH	43.76 ± 7.226 (0.427)	50.41 ± 11.07 (0.469)
Fmoc-Asn(Trt)-OH*	2.007 ± 0.3512 (0.443)	65.16 ± 21.05 (0.231)
Fmoc-Asp(OtBu)-OH	83.61 ± 35.98 (0.297)	151.8 ± 23.64 (0.748)
Fmoc-Arg(Pbf)-OH*	1.997 ± 0.3315 (0.365)	180.2 ± 51.90 (0.060)
Fmoc-Cys(Trt)-OH*	2.373 ± 0.4428 (0.136)	53.09 ± 13.28 (0.076)
Fmoc-Gln(Trt)-OH*	2.101 ± 0.3764 (0.526)	25.05 ± 7.100 (0.181)
Fmoc-Glu(OtBu)-OH	79.47 ± 40.84 (0.448)	51.63 ± 12.74 (0.518)
Fmoc-Gly-OH	81.84 ± 13.57 (0.348)	73.11 ± 16.46 (0.472)
Fmoc-His(Boc)-OH	1.744 ± 0.2894 (0.850)	34.24 ± 9.044 (0.228)
Fmoc-Ile-OH	147.5 ± 27.27 (0.420)	36.12 ± 7.641 (0.342)
Fmoc-Leu-OH	67.51 ± 11.16 (0.505)	24.03 ± 5.196 (0.394)
Fmoc-Lys(Boc)-OH	1.770 ± 0.2681 (0.438)	32.87 ± 9.041 (0.177)
Fmoc-Met-OH	60.03 ± 14.37 (0.552)	57.21 ± 15.28 (0.645)
Fmoc-Phe-OH	2.275 ± 0.3689 (0.385)	39.33 ± 10.86 (0.195)
Fmoc-Pro-OH	69.46 ± 12.92 (0.461)	78.13 ± 17.76 (0.508)
Fmoc-Ser(tBu)-OH	56.84 ± 22.26 (0.407)	170.3 ± 29.26 (0.800)
Fmoc-Thr(tBu)-OH	41.18 ± 11.59 (0.439)	57.21 ± 12.87 (0.364)
Fmoc-Trp(Boc)-OH	1.653 ± 0.3523 (0.420)	75.50 ± 24.22 (0.146)
Fmoc-Tyr(tBu)-OH	2.002 ± 0.3506 (0.395)	179.2 ± 60.88 (0.112)
Fmoc-Val-OH	173.5 ± 51.38 (0.061)	51.52 ± 9.745 (0.571)

* Aqueous 10% *i*-PrOH solution was used for preparation.

S2. Coupling Cycle Design for Fully Automated Aqueous SPPS of Substance P and GLP-1 (1-37)

S2.1. Guidelines for Determining the Number of Coupling Cycles

The number of coupling cycles at each residue was empirically determined based on prior synthetic experience, anticipated steric hindrance during chain elongation, and preliminary optimization studies.

S2.2. The Number of Coupling Cycles

Table S4. Coupling Cycles Used for Fully Automated Aqueous SPPS of Substance P.

Residue number	1	2	3	4	5	6	7	8	9	10	11
	R	P	K	P	Q	Q	F	F	G	L	M
Number of coupling cycles	**2**	**2**	**2**	**2**	**2**	**2**	**1**	**1**	**1**	**1**	**2**

Table S5. Coupling Cycles Used for Fully Automated Aqueous SPPS of GLP-1 (1-37).

Residue number	1	2	3	4	5	6	7	8	9	10
	H	D	E	F	E	R	H	A	E	G
Number of coupling cycles	**3***	**1**	**2**	**1**	**2**	**2**	**3***	**2**	**2**	**2**
Residue number	11	12	13	14	15	16	17	18	19	20
	T	F	T	S	D	V	S	S	Y	L
Number of coupling cycles	**2**	**1**	**2**	**1**	**1**	**1**	**1**	**1**	**1**	**1**
Residue number	21	22	23	24	25	26	27	28	29	30
	E	G	Q	A	A	K	E	F	I	A
Number of coupling cycles	**2**	**2**	**2**	**1**	**2**	**2**	**2**	**1**	**1**	**2**
Residue number	31	32	33	34	35	36	37			
	W	L	V	K	G	R	G			
Number of coupling cycles	**2**	**1**	**1**	**1**	**2**	**2**	**2**			

* 3: Triple coupling performed as double coupling followed by single coupling according to the programmed workflow shown in Scheme 2.

S3. Method of Cleavage and Global Deprotection

S3-1. Cleavage and Global Deprotection of H-Protected Peptide–Rink Amide TentaGel Resin, Example Peptide: GLP-1 (1-37)

After automated synthesis, the resulting H-protected peptide–Rink amide TentaGel resin was washed with chloroform, and then treated with a TFA cleavage cocktail (TFA/H_2O/thioanisole/ TIPS/DODT = 92:2:3:2:1) for 3 h at room temperature. The reaction mixture was filtered to remove the resin, and the filtrate was concentrated under a stream of nitrogen. Cold diethyl ether was then added to the residue to afford the crude peptide as a white precipitate. The crude peptide was subsequently subjected to HPLC analysis and mass spectrometry.

4. Characterization for Synthetic Peptides

4.1. HPLC Analysis

HPLC analysis was performed following a previously reported procedure.[1] Briefly, analyses were carried out on a Waters Alliance e2695 system equipped with a COSMOSIL 5C18-AR-II column (4.6 × 150 mm). Elution was conducted using a gradient of acetonitrile/water containing 0.1% trifluoroacetic acid (TFA) at a flow rate of 1.0 mL/min, with UV detection at 220 nm. Chromatographic data were processed using a consistent integration protocol. Peaks with a signal-to-noise ratio (S/N) ≥ 10 were integrated, while the solvent front and system-related artifacts were excluded. Valley-to-valley integration was applied when the valley depth exceeded 10% of the corresponding peak height. Crude purities were determined based on peak area percentages under these conditions. Purity values reported in the main text are rounded to the nearest integer, whereas more precise values are provided in the Supporting Information.

S4.2. Mass Analysis

Mass spectrometric analysis was performed as previously described.[1] Spectra were recorded on an electrospray ionization quadrupole time-of-flight mass spectrometer (ESI-Q-TOF-MS; micrOTOF-Q, Bruker Daltonik GmbH). Samples were prepared in acetonitrile/water containing 0.1% formic acid and introduced by direct infusion. Measurements were conducted in the positive ion mode over an appropriate m/z range. Observed signals are assigned to protonated molecular ions ($[M+H]^+$), unless otherwise noted.

S4.3. Leu-Enkephalin Amide

(a)

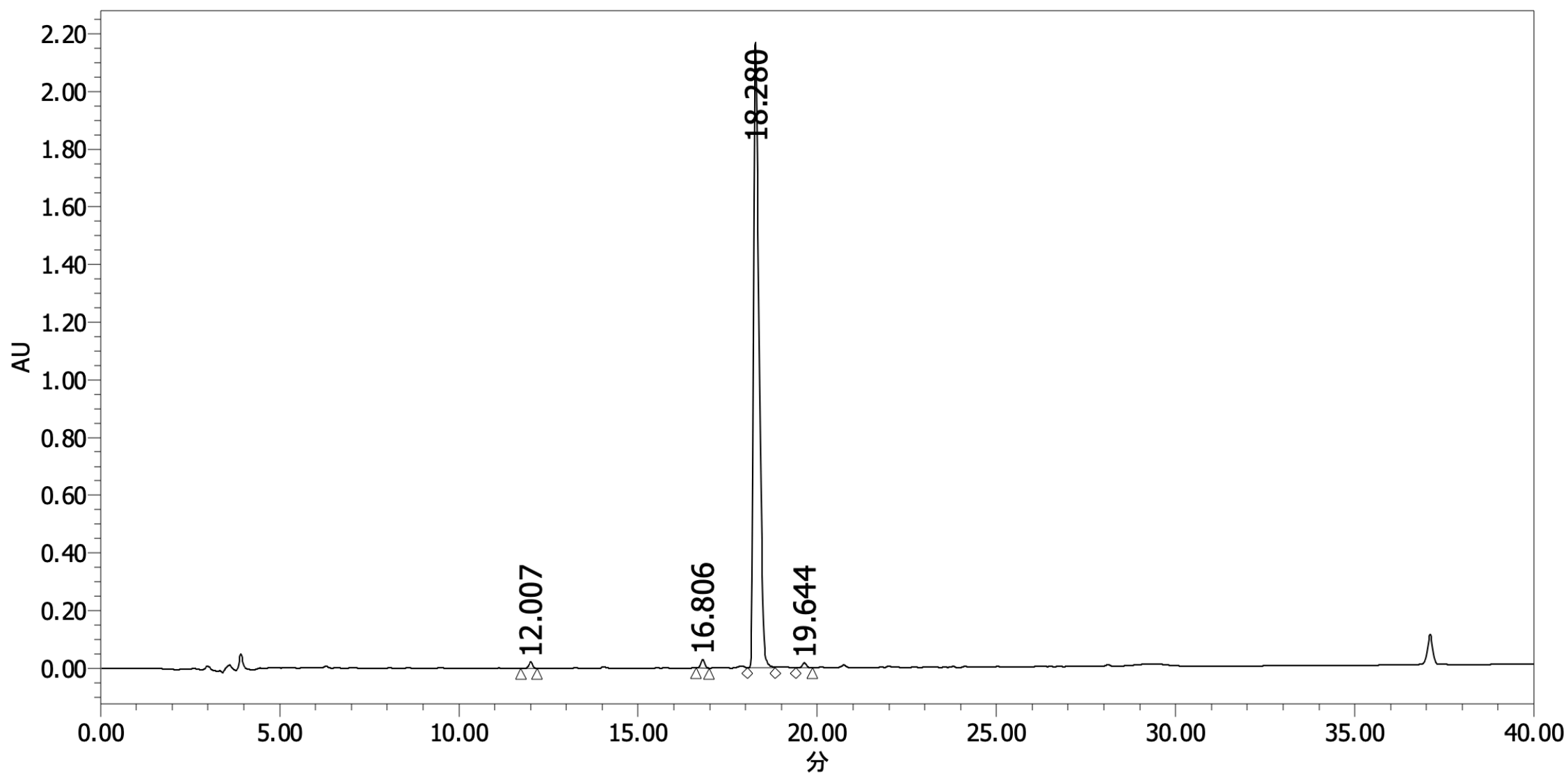

(b)

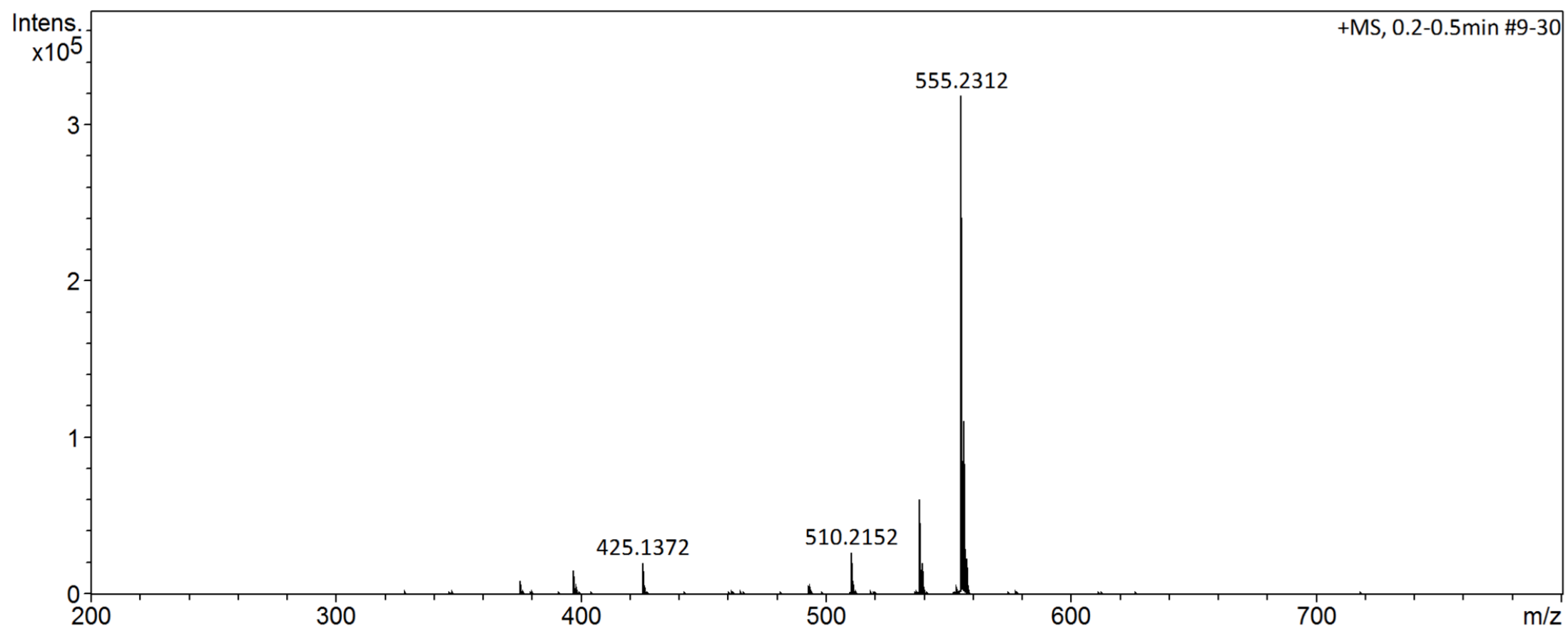

Figure S22. Analytical HPLC Profile and ESI-MS Spectrum of Leu-Enkephalin Amide Synthesized by Fully Automated Aqueous SPPS Using TBTU.

(a) Analytical HPLC profile of Leu-enkephalin amide obtained by fully automated aqueous SPPS using TBTU and nanoassemblies of Fmoc-amino acids with DIEA under programmed workflow shown in Scheme 1 (single coupling throughout). Elution was carried out over 40 min at a flow rate of 1 mL/min with a linear gradient from 90:10 to 50:50 mixture of 0.1% aqueous TFA and 0.1% TFA in acetonitrile. A dominant peak was observed at a retention time of 18.2 min with a calculated purity of 97.8 %; (b) ESI-MS (TOF) spectrum of the crude product. The observed mass was m/z 555.2312 ($[M+H]^+$), which corresponds to $C_{28}H_{39}N_6O_6$ (calculated: 555.2931).

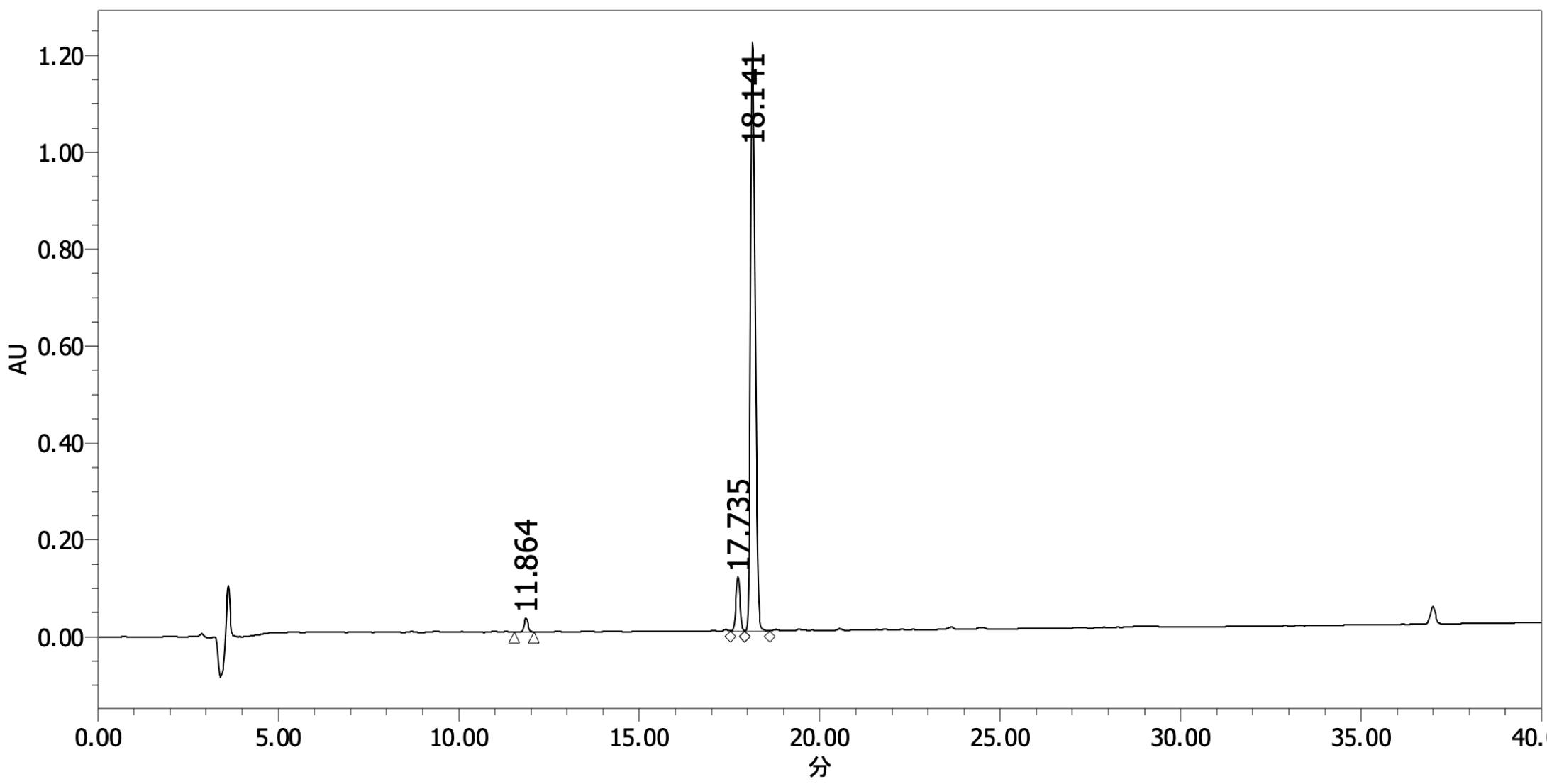

Figure S23. Analytical HPLC Profile of Leu-Enkephalin Amide Synthesized by Fully Automated Aqueous SPPS Using DMT-MM.

Analytical HPLC profile of Leu-enkephalin amide obtained by fully automated aqueous SPPS using DMT-MM and nanoassemblies of Fmoc-amino acids with NMM under the programmed workflow shown in Scheme 1 (single coupling throughout). Elution was carried out over 40 min at a flow rate of 1 mL/min with a linear gradient from 90:10 to 50:50 mixture of 0.1% aqueous TFA and 0.1% TFA in acetonitrile. A dominant peak was observed at a retention time of 18.1 min with a calculated purity of 91.0 %.

(a)

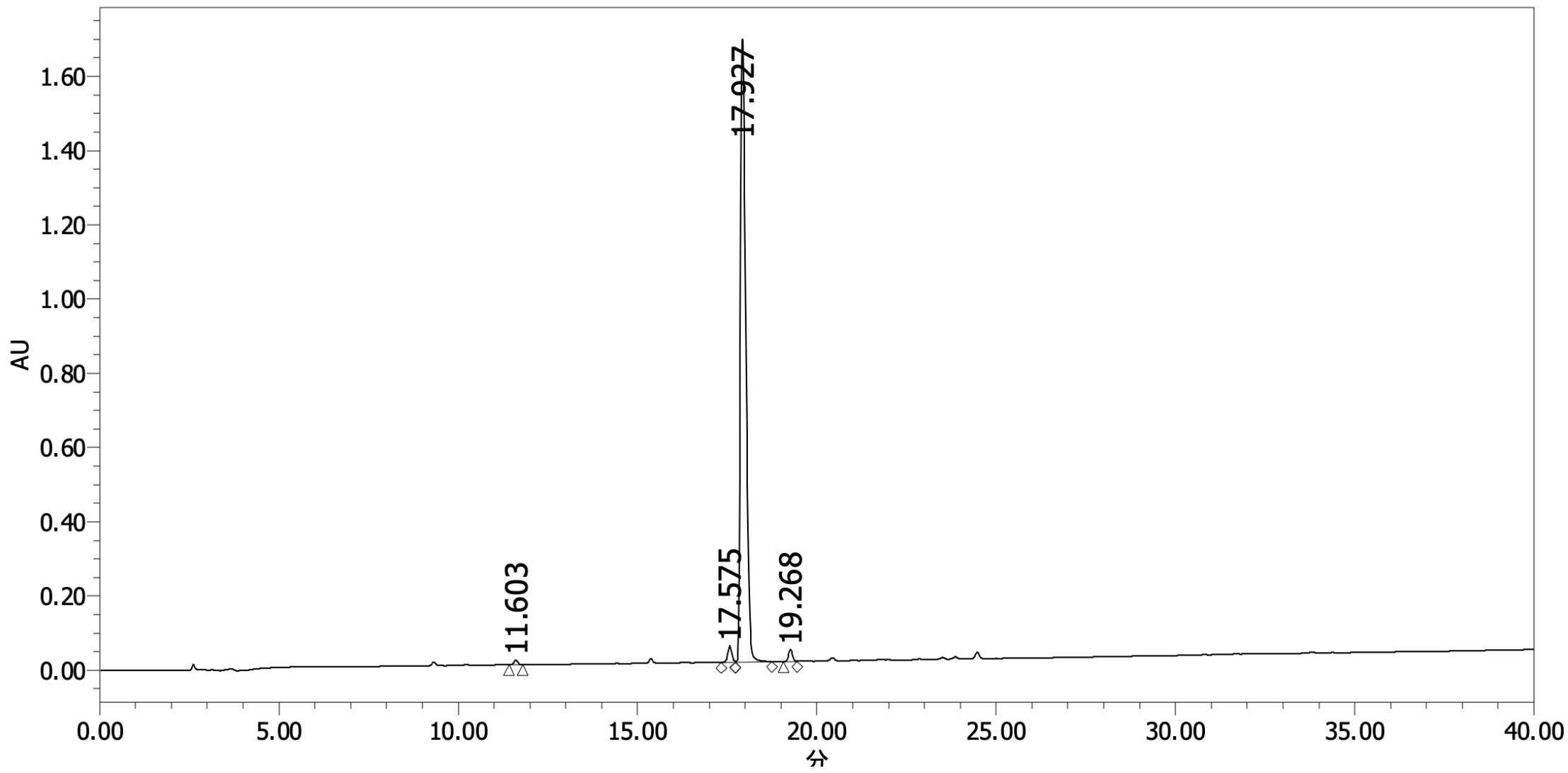

(b)

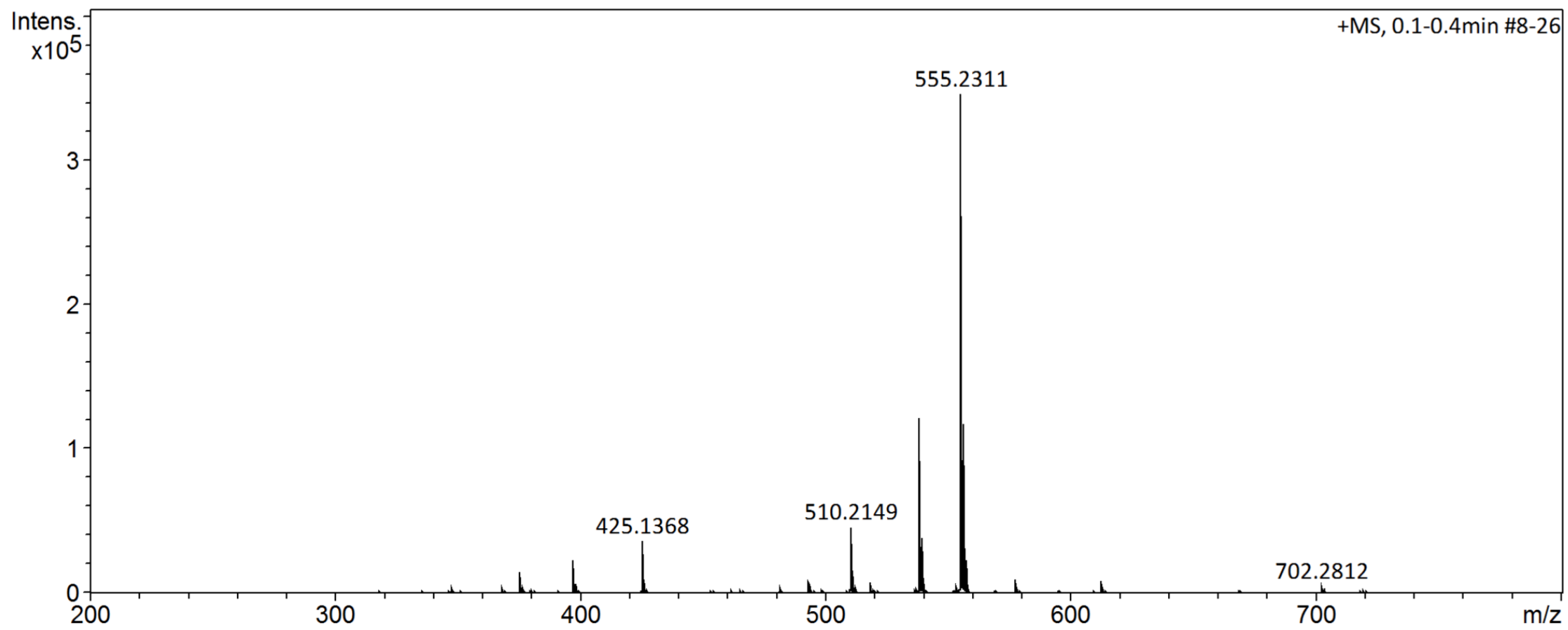

Figure S24. Analytical HPLC Profile and ESI-MS Spectrum of Leu-Enkephalin Amide Synthesized by Conventional Fmoc-SPPS in DMF.

(a) Analytical HPLC profile of Leu-enkephalin amide obtained by conventional Fmoc-SPPS in DMF. Elution was carried out over 40 min at a flow rate of 1 mL/min with a linear gradient from 90:10 to 50:50 mixture of 0.1% aqueous TFA and 0.1% TFA in acetonitrile. A dominant peak was observed at a retention time of 17.9 min with a calculated purity of 96.23 %; (b) ESI-MS (TOF) spectrum of the crude product. The observed mass was m/z 555.2311 ($[M+H]^+$), which corresponds to $C_{28}H_{39}N_6O_6$ (calculated: 555.2931).

S4.4. Substance P

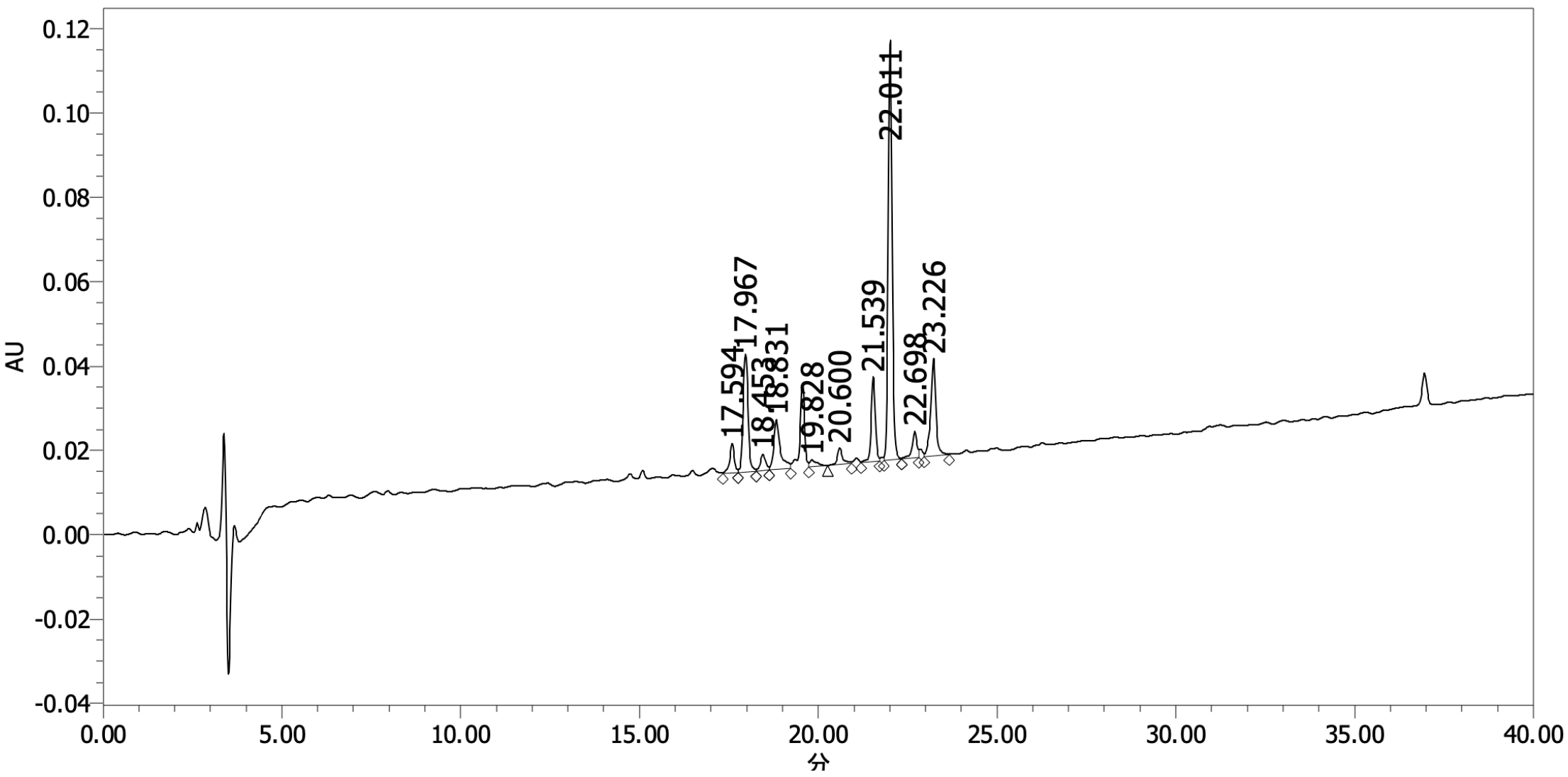

Figure S25. Analytical HPLC Profile of Substance P Synthesized by Fully Automated Aqueous SPPS Using TBTU According to Scheme 1.

Analytical HPLC profile of Substance P obtained by fully automated aqueous SPPS using TBTU and nanoassemblies of Fmoc-amino acids with DIEA under programmed workflow shown in Scheme 1 (single coupling throughout). Elution was carried out over 40 min at a flow rate of 1 mL/min with a linear gradient from 90:10 to 50:50 mixture of 0.1% aqueous TFA and 0.1% TFA in acetonitrile. A major peak was observed at a retention time of 22.01 min with a calculated purity of 42.7%.

(a)

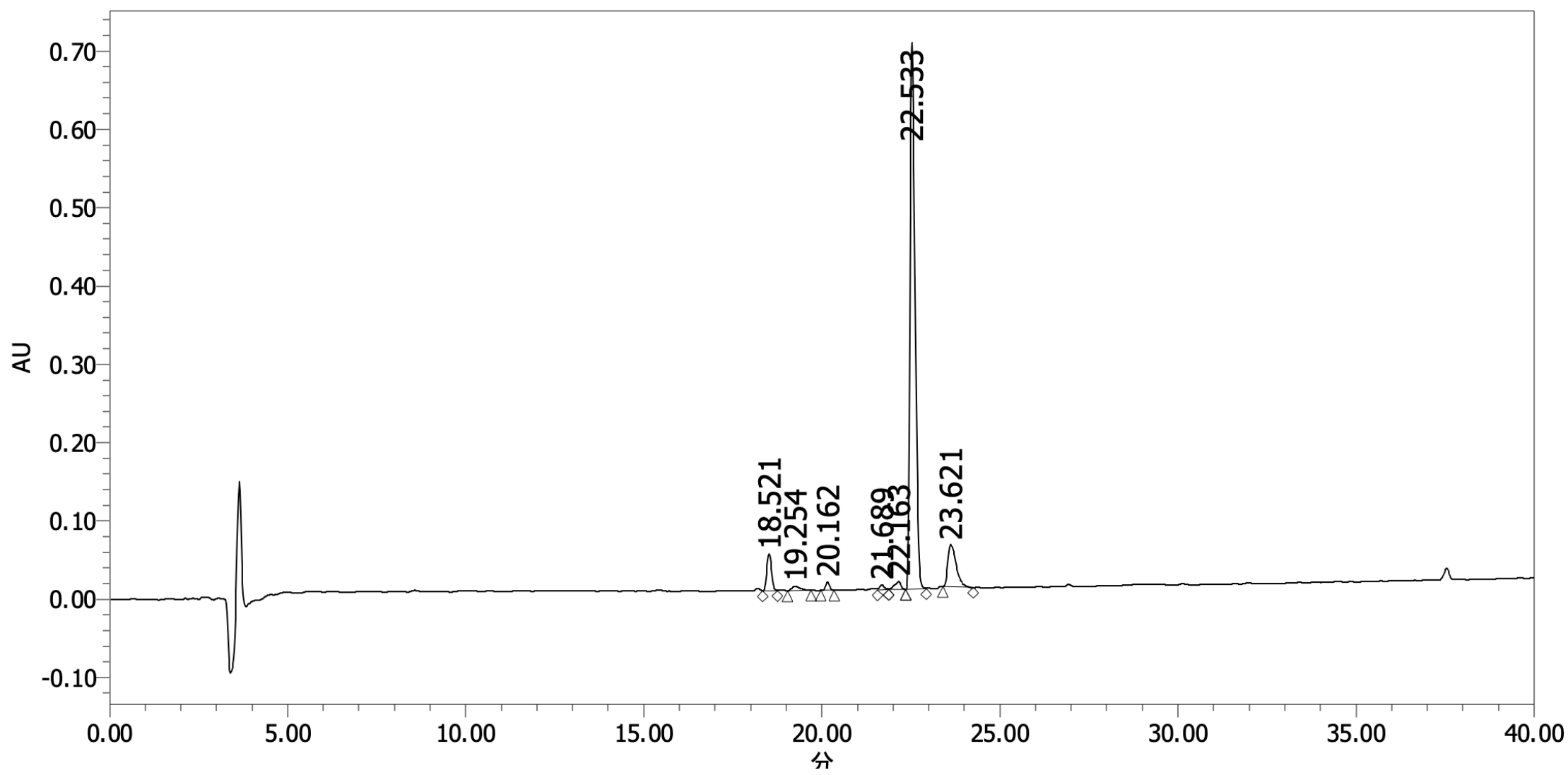

(b)

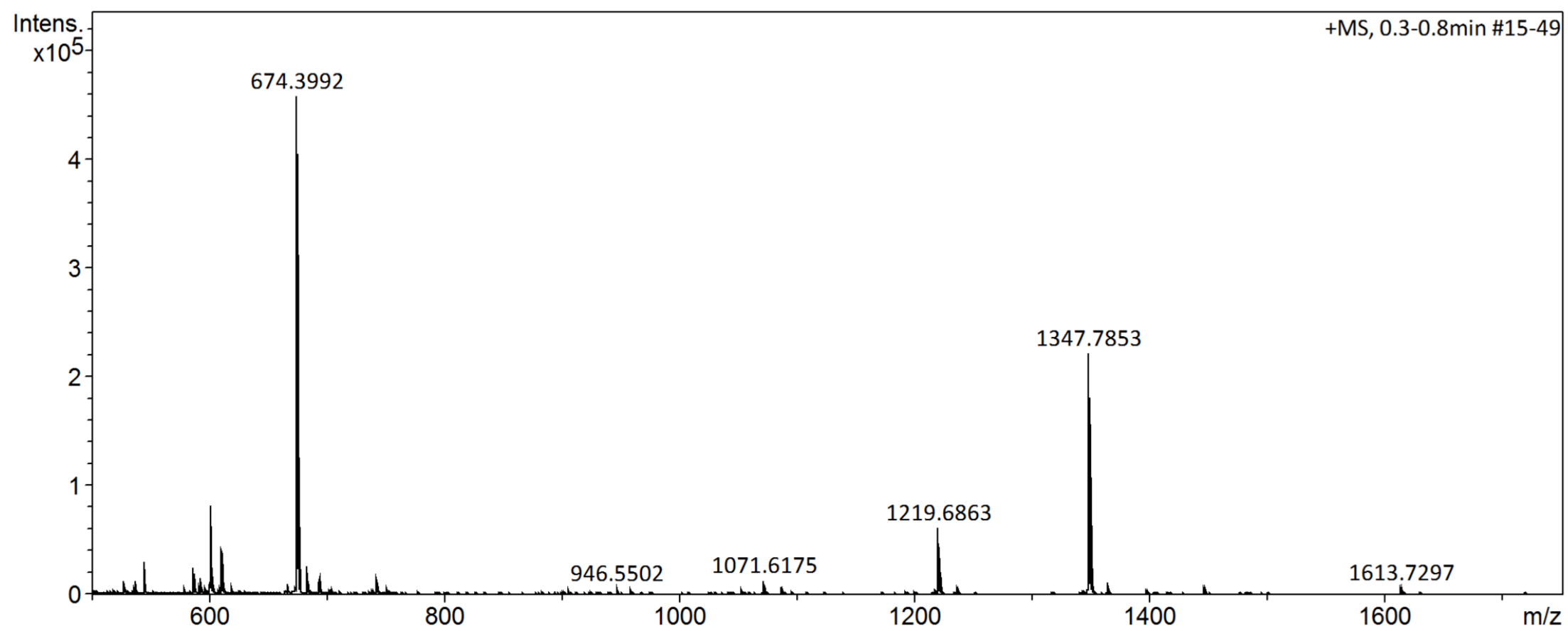

Figure S26. Analytical HPLC Profile and ESI-MS Spectrum of Substance P Synthesized by Fully Automated Aqueous SPPS Using TBTU According to Scheme 2.

(a) Analytical HPLC profile of Substance P obtained by fully automated aqueous SPPS using TBTU and nanoassemblies of Fmoc-amino acids with DIEA under the programmed workflow shown in Scheme 2 and the coupling schedule summarized in Table S3; Elution was carried out over 40 min at a flow rate of 1 mL/min with a linear gradient from 90:10 to 50:50 mixture of 0.1% aqueous TFA and 0.1% TFA in acetonitrile. A dominant peak was observed at a retention time of 22.5 min with a calculated purity of 80.8 %; (b) ESI-MS (TOF) spectrum of the crude product. The observed mass was m/z 1347.7373 ($[M+H]^+$), which corresponds to $C_{63}H_{98}N_{19}O_{13}S$ (calculated: 1347.728).

(a)

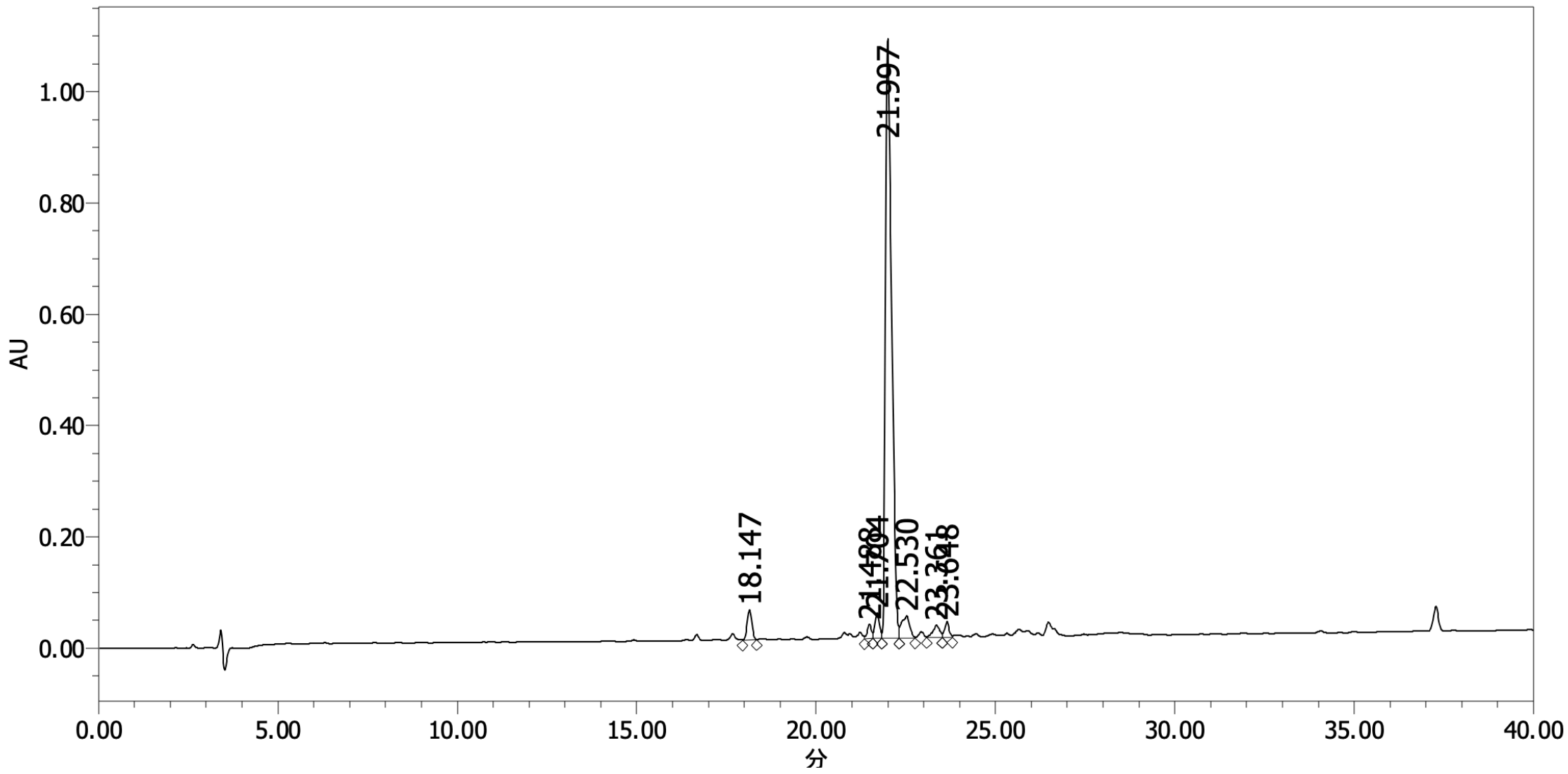

(b)

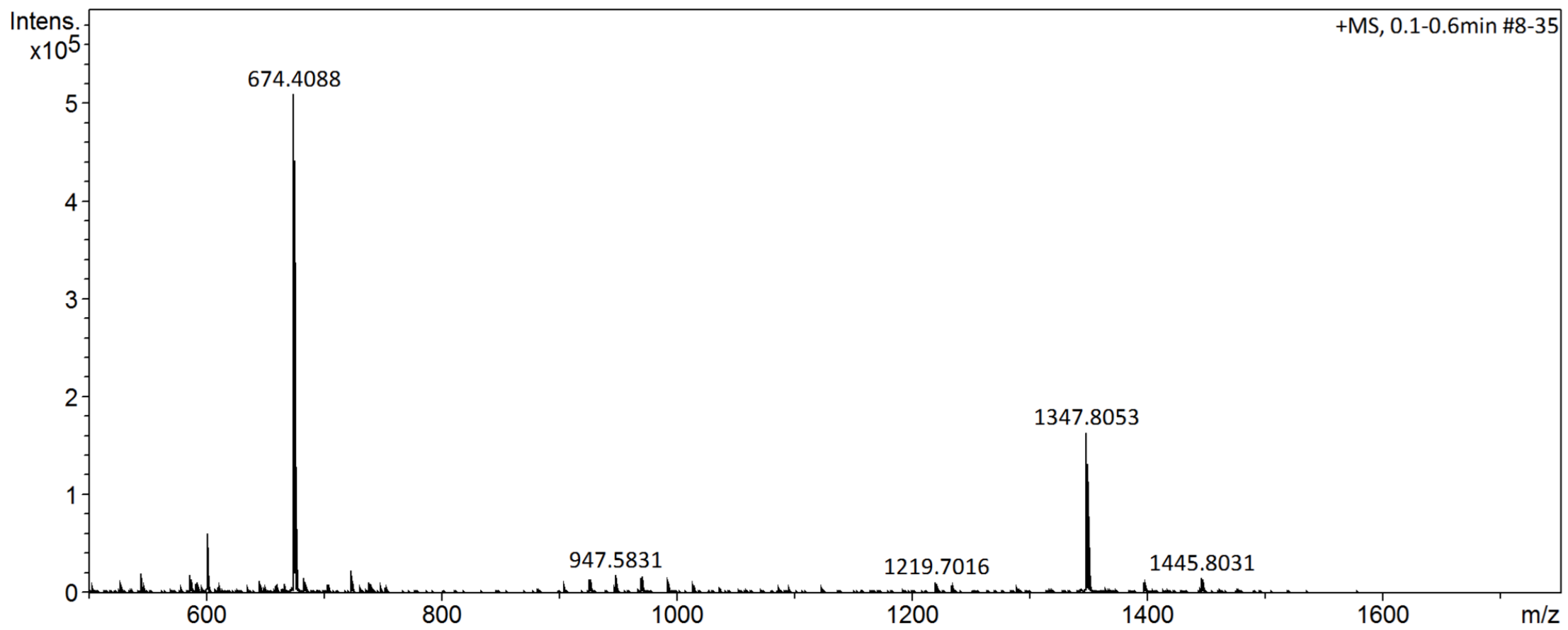

Figure S27. Analytical HPLC Profile and ESI-MS Spectrum of Substance P Synthesized by Conventional Fmoc-SPPS in DMF.

(a) Analytical HPLC profile of Substance P obtained Synthesized by Conventional Fmoc-SPPS in DMF; Elution was carried out over 40 min at a flow rate of 1 mL/min with a linear gradient from 90:10 to 50:50 mixture of 0.1% aqueous TFA and 0.1% TFA in acetonitrile. A dominant peak was observed at a retention time of 22.0 min with a calculated purity of 85.0 %; (b) ESI-MS (TOF) spectrum of the crude product. The observed mass was m/z 1347.8053 ($[M+H]^+$), which corresponds to $C_{63}H_{98}N_{19}O_{13}S$ (calculated: 1347.728).

S4.5. GLP-1 (1-37)

(a)

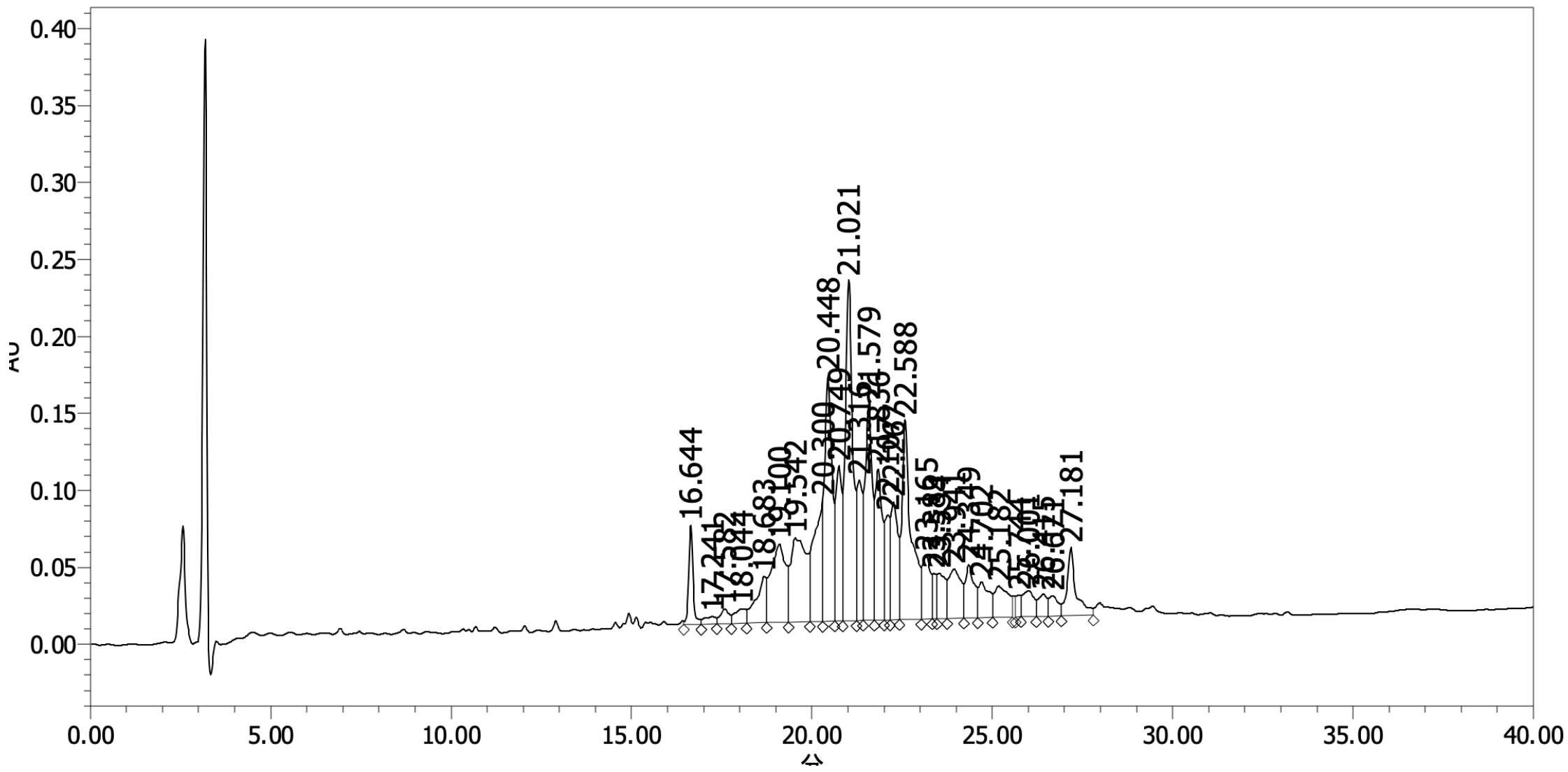

(b)

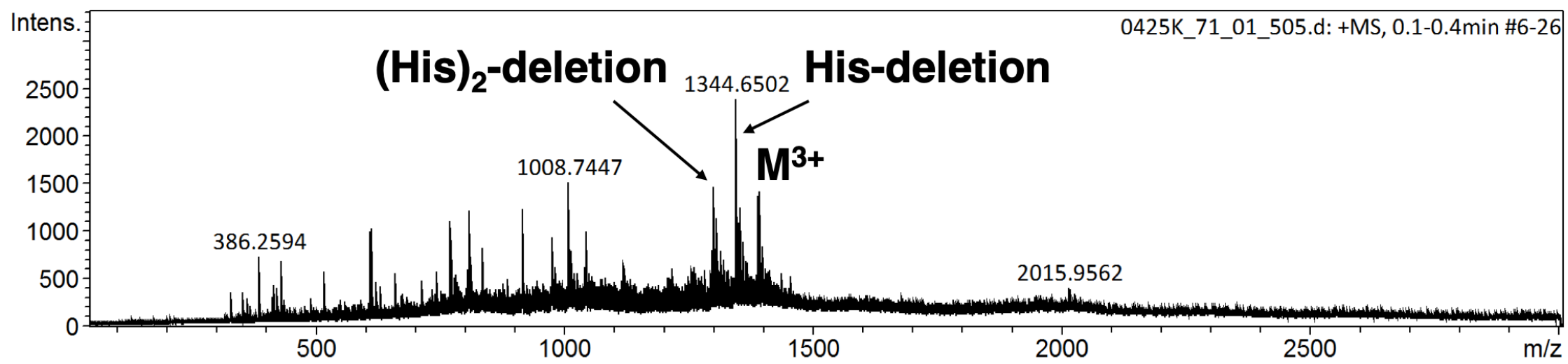

Figure S28. Analytical HPLC Profile and ESI-MS Spectrum of GLP-1 (1-37) Synthesized by Fully Automated Aqueous SPPS Using TBTU.

(a) analytical HPLC profile of GLP-1 (1–37) obtained by fully automated aqueous SPPS using TBTU and nanoassemblies of Fmoc-amino acids with DIEA under the programmed workflow shown in Scheme 2. Double coupling was applied at histidine positions.; Elution was carried out over 40 min at a flow rate of 1 mL/min with a linear gradient from 80:10 to 40:60 mixture of 0.1% aqueous TFA and 0.1% TFA in acetonitrile. The HPLC profile was highly complex, with multiple overlapping peaks, making the assignment of the target product difficult; (b) ESI-MS (TOF) spectrum of crude compound. Mass spectrometric analysis indicated that deletion species were predominant, although signals consistent with the intact peptide were also observed at low intensity. A triply charged species was detected at m/z 1390.6670 ($[M+3H]^{3+}$) which corresponds to $C1_{86}H_{276}N_{52}O_{58}$ (calculated monoisotopic mass: 4166.0246).

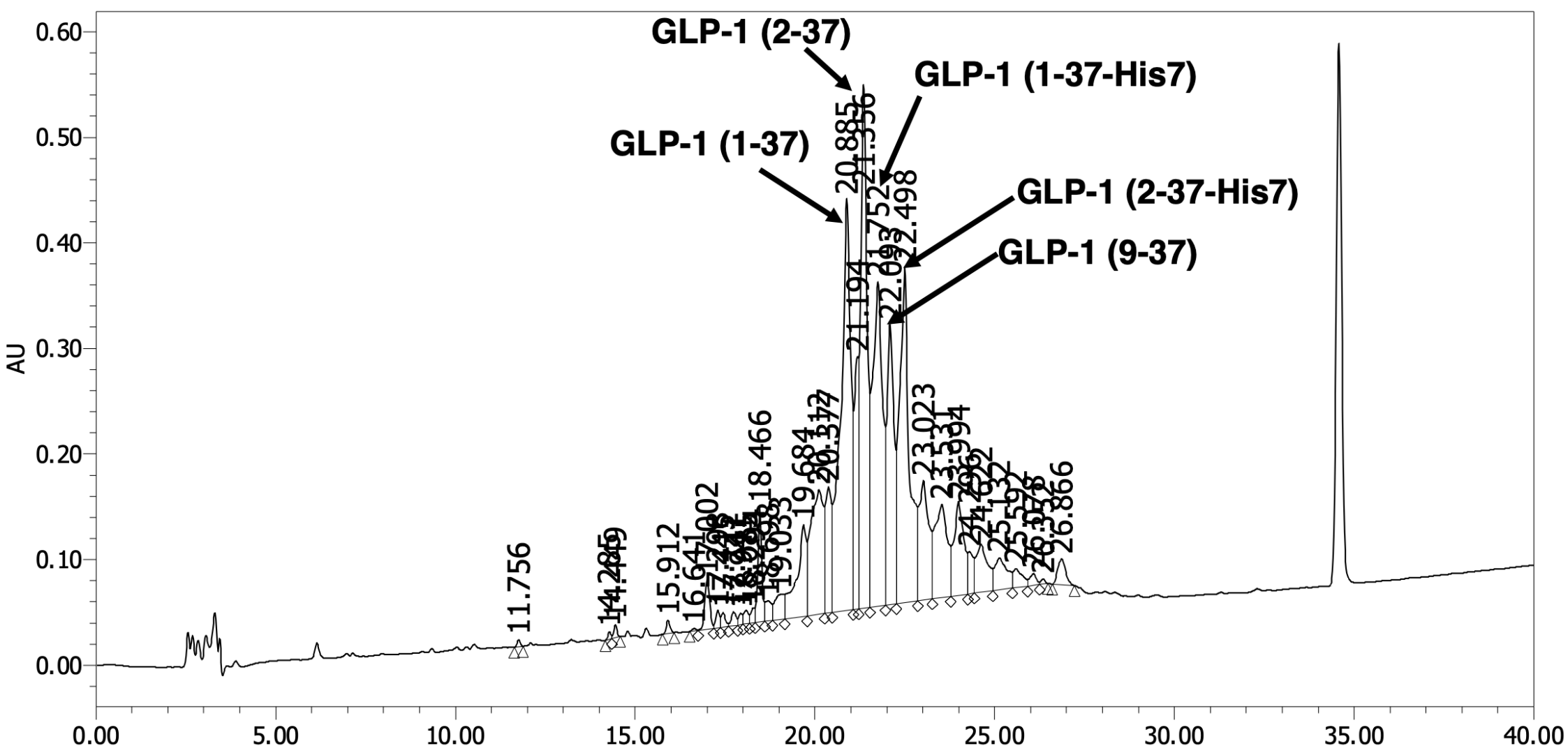

Figure S29. Co-Injection Analysis for Identification of His-Deletion Products in Crude GLP-1(1-37).

HPLC co-injection analysis of the crude GLP-1(1-37) reaction mixture (Fig. S28A) with independently synthesized standards, confirming the identity of the major deletion products present in the crude mixture: GLP(1-37) (target peptide), GLP(2-37) (loss of the N-terminal His), GLP(1-37-His7) (loss of the second His), GLP(2-37-His7) (loss of both His residues), and GLP(9-37) (loss of the *N*-terminal 8 residues). Peak assignments and retention times are indicated in the figure.; Elution was carried out over 40 min at a flow rate of 1 mL/min with a linear gradient from 80:10 to 40:60 mixture of 0.1% aqueous TFA and 0.1% TFA in acetonitrile.

(a)

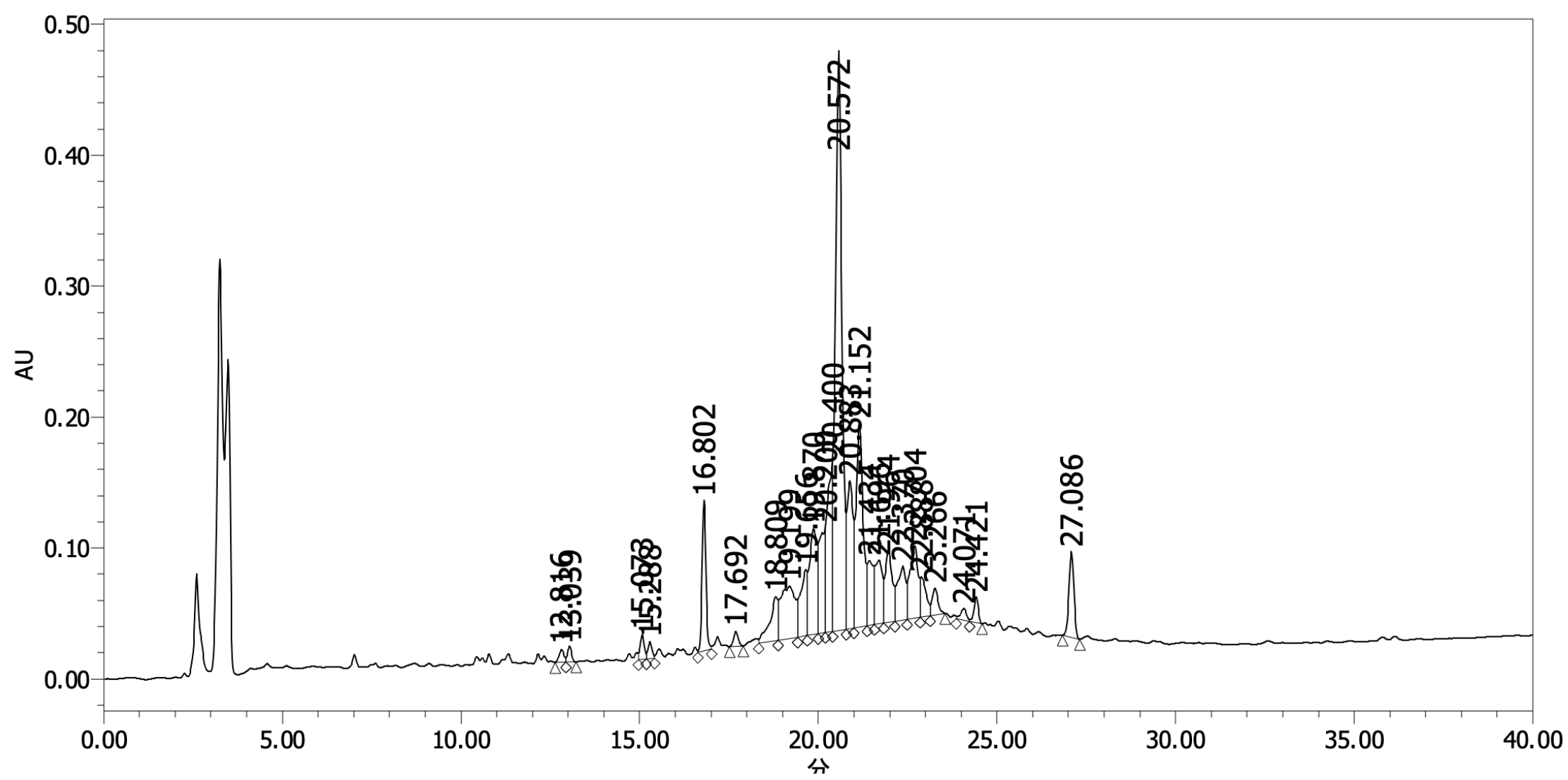

(b)

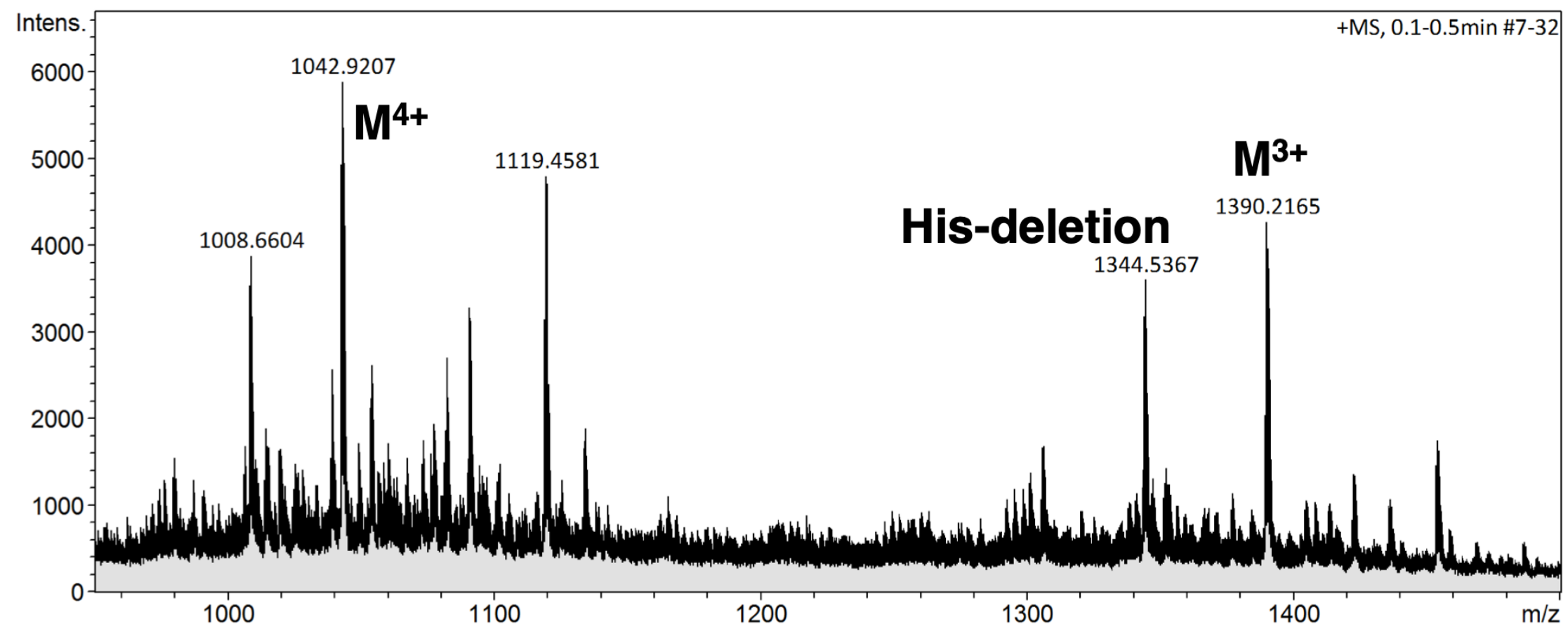

Figure S30. Analytical HPLC Profile and ESI-MS Spectrum of GLP-1 (1-37) Synthesized by Fully Automated Aqueous SPPS Using TBTU with DMT-MM at His position.

(a) Analytical HPLC profile of GLP-1 (1-37) obtained by fully automated aqueous SPPS using TBTU and nanoassemblies of Fmoc-amino acids with DIEA under the programmed workflow shown in Scheme 1 and the coupling schedule summarized in Table S4. DMT-MM was selectively used for histidine residues.; Elution was carried out over 40 min at a flow rate of 1 mL/min with a linear gradient from 80:20 to 40:60 mixture of 0.1% aqueous TFA and 0.1% TFA in acetonitrile. A dominant peak was observed at a retention time of 20.6 min with a calculated purity of 16.3 %; (b) ESI-MS (TOF) spectrum of crude compound. A triply charged species was detected at m/z 1390.2165 ($[M+3H]^{3+}$) which corresponds to $C_{186}H_{276}N_{52}O_{58}$ (calculated monoisotopic mass: 4166.0246).

(a)

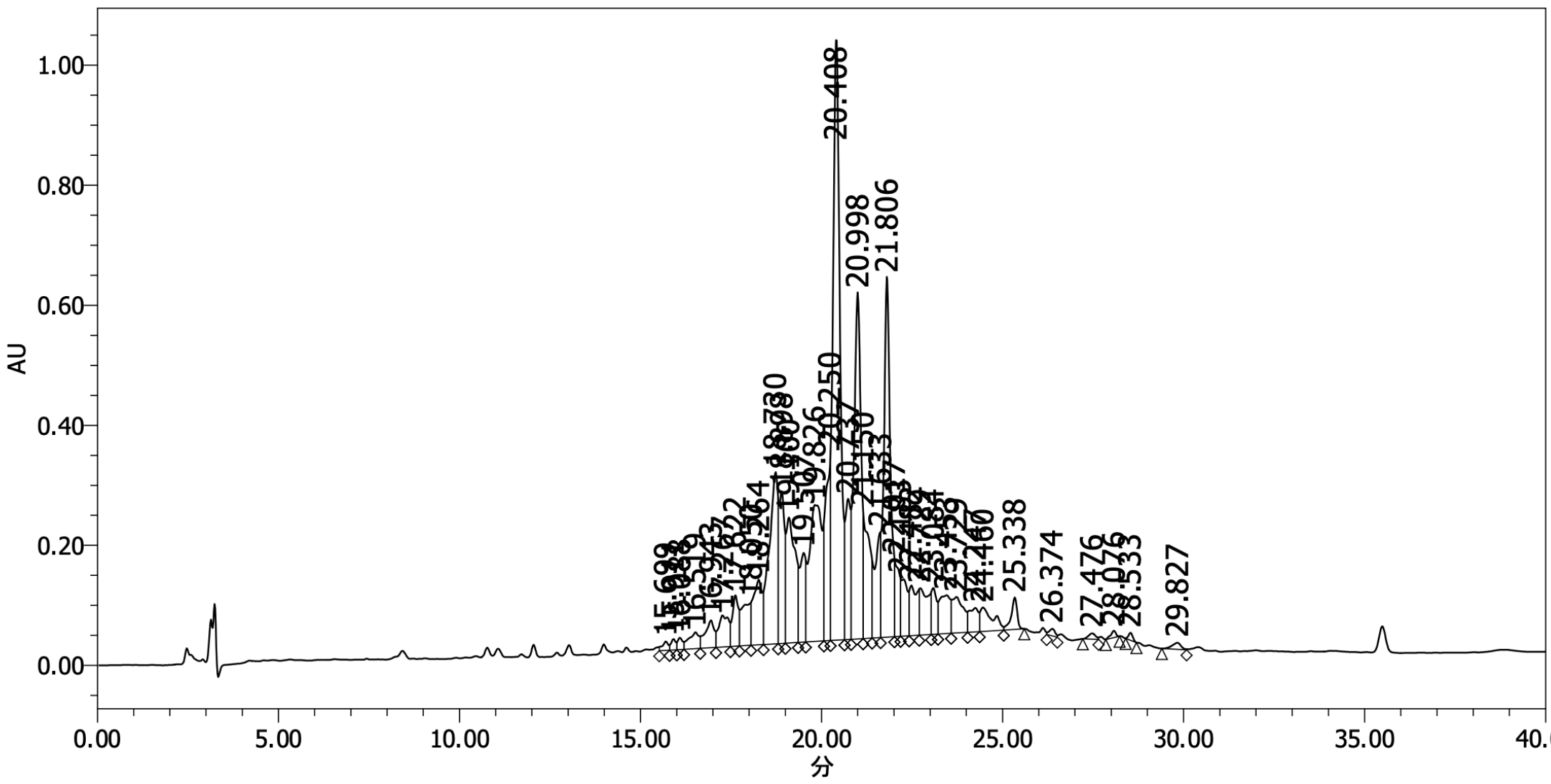

(b)

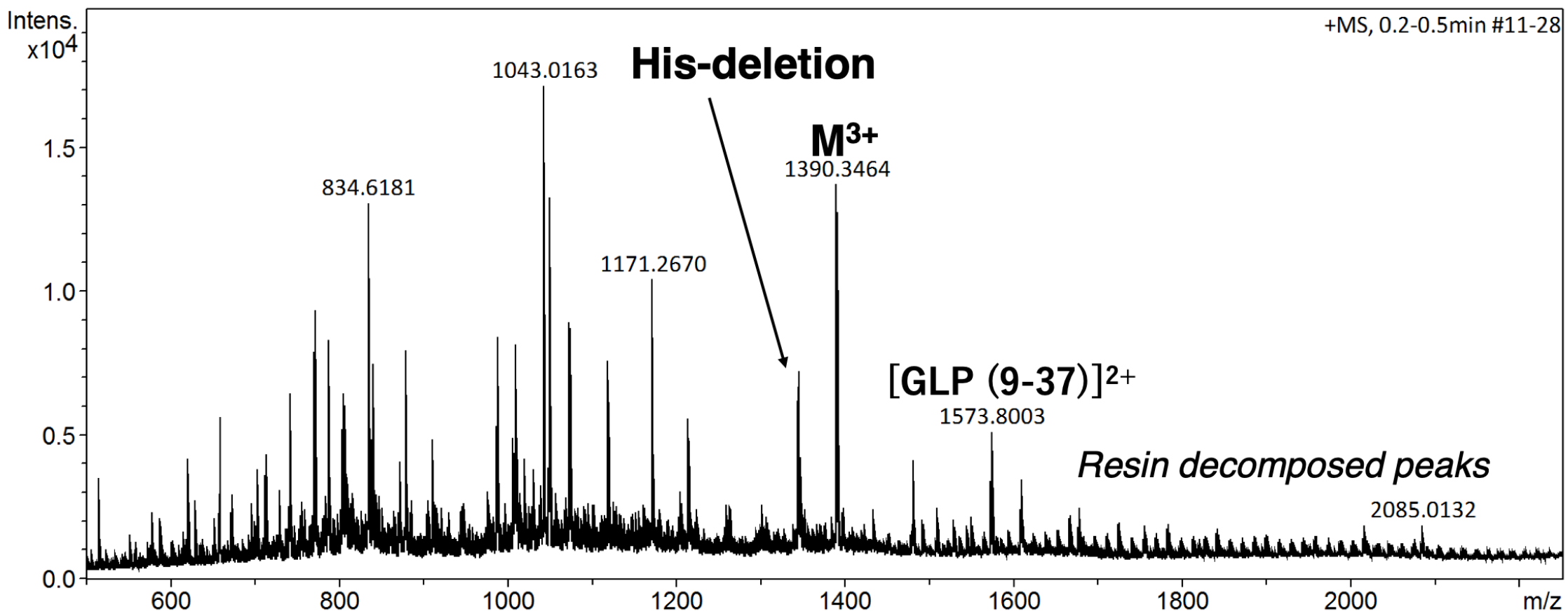

Figure S31. Analytical HPLC Profile and ESI-MS Spectrum of GLP-1 (1-37) Synthesized by Conventional Fmoc-SPPS in DMF.

(a) Analytical HPLC profile of crude GLP-1 (1-37) obtained by conventional SPPS in DMF employing Fmoc strategy. Elution was carried out over 40 min at a flow rate of 1 mL/min with a linear gradient from 80:20 to 40:60 mixture of 0.1% aqueous TFA and 0.1% TFA in acetonitrile. A dominant peak was observed at a retention time of 26.3 min with a calculated purity of 18%; (b) ESI-MS (TOF) spectrum of crude peptide. A triply charged species was detected at m/z 1390.3435 ($[M+3H]^{3+}$) which corresponds to $C_{186}H_{276}N_{52}O_{58}$ (calculated monoisotopic mass: 4166.0246).

S5. PMI

S5.1. Calculation of Process Mass Intensity (PMI)[10]

Process Mass Intensity (PMI), a standard green chemistry metric, was calculated as the total mass of all materials used in the synthesis (reagents, resin, and process solvents) divided by the mass of the isolated product:

PMI = (Total mass of materials used, mg) / (Mass of isolated product, mg)

Solvent volumes were converted to mass using standard literature density values (Water, 1.00 g/mL; AcOEt, 0.902 g/mL; 4-methylpiperidine, 0.849 g/mL; piperidine, 0.862 g/mL; DMF, 0.944 g/mL; NMM, 0.920 g/mL; TFA, 1.489 g/mL; triisopropylsilane (TIS), 0.822 g/mL; diethyl ether, 0.713 g/mL). Reagents used for Fmoc deprotection during the coupling cycles (4-methylpiperidine for the aqueous protocol; piperidine for the DMF-based protocol) were included in the calculation, as they differ between the two synthetic routes. The final global deprotection/resin cleavage step (TFA/TIS/H2O, 2 mL : 40 uL : 40 uL) and subsequent precipitation (diethyl ether, 20 mL), which were common to both routes, were also included in the calculation. Solvents used for HPLC purification were excluded from the calculation, consistent with the use of crude (pre-purification) product mass in the denominator.

Table S6. Materials Used for PMI Calculation of Leu-Enkephalin Amide Synthesis by ASPPS and Conventional Fmoc-SPPS in DMF

Material	MW (g/mol)	ASPPS (mg)	In-DMF synthesis (mg)
Fmoc-Leu-OH	353.4	35.34	35.34
Fmoc-Phe-OH	387.4	38.74	38.74
Fmoc-Gly-OH	297.3	59.46	29.73
Fmoc-Tyr(tBu)-OH	459.6	45.96	45.96
TBTU	321.0	32.10	32.10
DIEA	129.2	15.50	38.76
HOBt	135.1	0.00	13.51
Water	–	55,000.00	0.00
2-propanol	–	98,125.00	0.00
AcOEt	–	129,076.20	0.00
4-methylpiperidine	–	5,433.60	0.00
piperidine	–	0.00	10,292.28
DMF	–	0.00	243,334.88
NMM	–	0.00	552.00
TFA	–	2,978.00	2,978.00
TIS	–	32.88	32.88
Water (cleavage)	–	40.00	40.00

Material	MW (g/mol)	ASPPS (mg)	In-DMF synthesis (mg)
Diethyl ether	–	14,260.00	14,260.00
Resin	–	100.0	100.0
Total mass input		305,272.8	271,824.2
Isolated product yield		16.1	15.7
PMI		19,000	17,300

S5.2. PMI Comparison Between Aqueous SPPS (ASPPS) and Conventional Fmoc-SPPS in DMF

As summarized in Table S6, the PMI values calculated for the synthesis of Leu-Enkephalin amide, including the final global deprotection/cleavage and precipitation steps, were approximately 19,000 for the fully automated aqueous SPPS (ASPPS) protocol and approximately 17,300 for the conventional Fmoc-SPPS protocol in DMF. Despite the complete elimination of DMF in the aqueous process, the two PMI values were of comparable magnitude, reflecting the substantial volume of water required in the ASPPS protocol. Nevertheless, the ASPPS method offers a clear advantage from an environmental and safety standpoint, as it entirely replaces DMF, a solvent of significant toxicological and environmental concern, with water and lower-hazard organic solvents (2-propanol and ethyl acetate). These PMI values, on the order of 10^4, are consistent with the general trend observed in solid-phase peptide synthesis (SPPS), which typically exhibits substantially higher PMI than small-molecule synthesis owing to the use of excess reagents at each coupling step and the repeated resin-washing steps required throughout the synthetic cycle. It should also be noted that PMI is inherently scale-dependent: fixed operational minima, such as cleavage and washing volumes, do not scale proportionally with reaction scale. The present PMI values were calculated at a research scale of 0.025 mmol, using a calculation boundary (crude, pre-purification product mass; inclusion of the global deprotection/cleavage step) consistent with a recently reported PMI framework for peptide synthesis; the substantially larger PMI values obtained here relative to those reported at larger research scales (e.g., ≥0.64 mmol) in that framework are attributable to this scale difference.

S6. Coupling Reagents

S6.1. Structure of Coupling Reagents Used in This Study

DMT-MM: 4-(4,6-Dimethoxy-1,3,5-triazin-2-yl)-4-methylmorpholinium chloride[7,8]

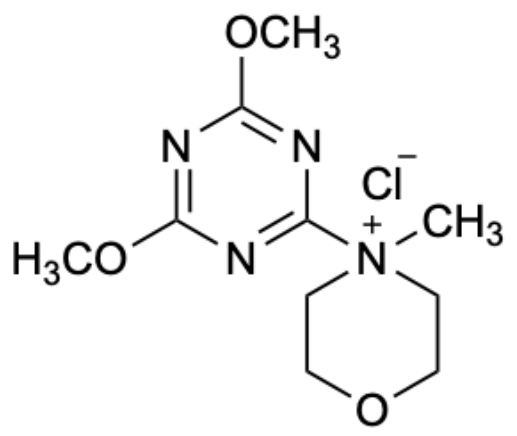

TBTU: 2-(1H-Benzotriazole-1-yl)-1,1,3,3-tetramethylaminium tetrafluoroborate[9]

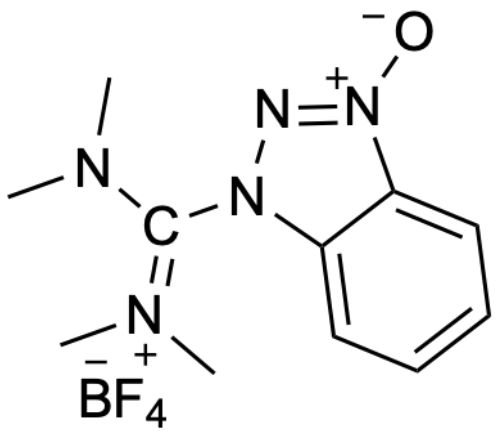

TATU: 1-[Bis(dimethylamino)methylene]-1H-1,2,3-triazolo[4,5-b]pyridinium 3-oxide[9] tetrafluoroborate

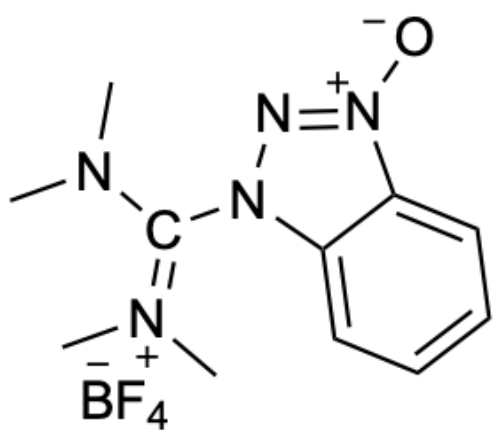

TCTU: 1-[Bis(dimethylamino)methylen]-5-chlorobenzotriazolium 3-oxide tetrafluoroborate[9]

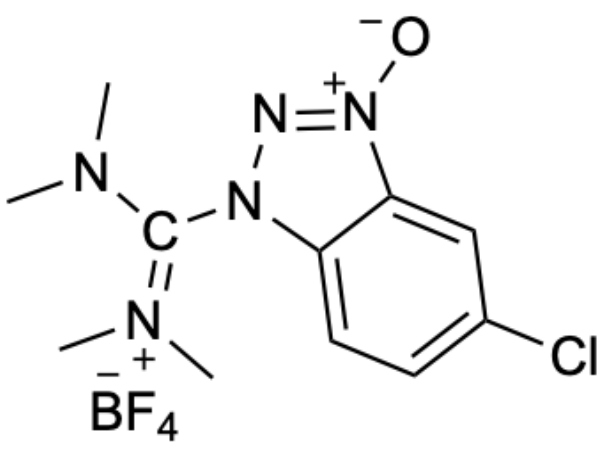

S7. References

1. Hojo, K.; Nonaka, A.; Manabe, Y.; Rentier, C.; Mehrotra, A.; Hioki, K.; Kunishima, M. *RSC Adv.* 2026, **16**, 18073–18082.
2. ISO 22412:2017, Particle size analysis — Dynamic light scattering (DLS), International Organization for Standardization, Geneva, 2017.
3. B. J. Frisken, *Appl. Opt.*, 2001, **40**, 4087–4091.
4. N. Farkas and J. A. Kramar, *J. Nanopart. Res.*, 2021, **23**, 120.
5. Malvern Panalytical, "What is the Polydispersity Index (PDI) in Dynamic Light Scattering (DLS)?", Knowledge Center, 2025.
6. NanoTemper Technologies, "Dynamic light scattering (DLS) — Cumulant Analysis vs. Size Distribution Analysis", Technical Note.
7. M. Kunishima, C. Kawachi, J. Morita, K. Terao, F. Iwasaki and S. Tani, *Tetrahedron*, 1999, **55**, 13159–13170.
8. M. Kunishima, C. Kawachi, K. Hioki, K. Terao and S. Tani, *Tetrahedron*, 2001, **57**, 1551–1558.
9. F. Albericio and A. El-Faham, *Org. Process Res. Dev.*, 2018, **22**, 760–772.
10. A. Sharma, A. Kumar, I. R. Singh, P. Kushwaha, B. G. de la Torre and F. Albericio, *Org. Process Res. Dev.*, 2026, **30**, 2346–2359.
